# Supplementary material for: Soil microbiomes show consistent and predictable responses to extreme events
Source: Nature. 2024 Nov 27;636(8043):690–6. doi: 10.1038/s41586-024-08185-3 (PMC11655354; doi:10.1038/s41586-024-08185-3)
Supplement: Supplementary file 1 — This file contains information on both the sampled sites (Supplementary Table 1) and individual soil properties, enzyme activities, gas fluxes, substrate-induced respiration and soil moisture content throughout the experiment (Supplementary Figs. 1–4). [file 41586_2024_8185_MOESM1_ESM.pdf]

nature portfolio

https://doi.org/10.1038/s41586-024-08185-3

Supplementary information

Soil microbiomes show consistent and predictable responses to extreme events

In the format provided by the authors and unedited

Supplementary Table and Figures

Supplementary Table 1. Site description for all sampling locations. A full set of metadata is available (see Data availability statement in main text).

Replicate site	*Soil class WRB*	*pH*	*Total soil carbon (%)*	*MAT*	*MAP*	*Latitude*	*Longitude*
GR1	Haplic Luvisols	7.57	3.2	13.4	482	40.62	23.07
GR2	Haplic Luvisols	7.71	1.8	13.4	482	40.62	23.06
GR3	Haplic Cambisols	7.04	2.5	13.4	482	40.62	23.08
ES1	Haplic Cambisols	7.43	3.4	14.5	416	40.15	-3.50
ES2	Petric Calcisols	7.89	9.0	14.5	416	40.13	-3.51
ES3	Haplic Cambisols	7.44	4.4	14.5	416	40.13	-3.53
EE1	Rendzic Leptosols	7.49	6.1	5.7	604	58.71	23.79
EE2	Haplic Cambisols	7.51	6.9	5.7	591	58.74	23.70
EE3	Haplic Gleysols	7.33	5.8	5.7	579	58.73	23.60
DE1	Haplic Cambisols	7.32	3.1	8.8	502	51.39	11.87
DE2	Haplic Cambisols	7.02	2.6	8.9	493	51.39	11.88
DE3	Haplic Cambisols	6.88	2.4	8.9	493	51.39	11.88
UKOx1	Haplic Cambisols	7.22	8.7	9.7	627	51.77	-1.33
UKOx2	Haplic Cambisols	6.85	8.1	9.7	627	51.77	-1.33
UKOx3	Haplic Cambisols	7.08	12.9	9.8	618	51.77	-1.33
AT1	Haplic Podzols	4.73	3.5	-1.3	1383	47.13	12.62
AT2	Haplic Podzols	5.52	6.9	-0.4	1324	47.13	12.63
AT3	Haplic Podzols	4.81	4.7	-1.3	1383	47.13	12.62
IS1	Haplic Cambisols	6.73	1.8	3.4	604	65.77	-18.23
IS2	Haplic Podzols	6.23	4.7	3.4	604	65.77	-18.25
IS3	Haplic Podzols	6.19	10.8	3.1	623	65.77	-18.25
UKLa1	Haplic Podzols	5.87	3.5	8.6	1147	54.01	-2.79
UKLa2	Haplic Cambisols	5.72	3.7	8.6	1147	54.01	-2.77
UKLa3	Haplic Cambisols	5.78	3.6	8.6	1147	54.01	-2.77
SE1	Haplic Cambisols	6.45	11.7	-2.0	476	68.29	19.17
SE2	Haplic Cambisols	5.29	7.5	-0.5	435	68.30	19.17
SE3	Haplic Cambisols	5.70	4.8	-0.5	435	68.30	19.17
RU1	Haplic Kastanozems	6.73	1.4	10.7	223	47.91	45.38
RU2	Haplic Kastanozems	6.78	1.9	10.7	223	47.91	45.38
RU3	Haplic Kastanozems	6.92	1.6	10.7	223	47.91	45.38

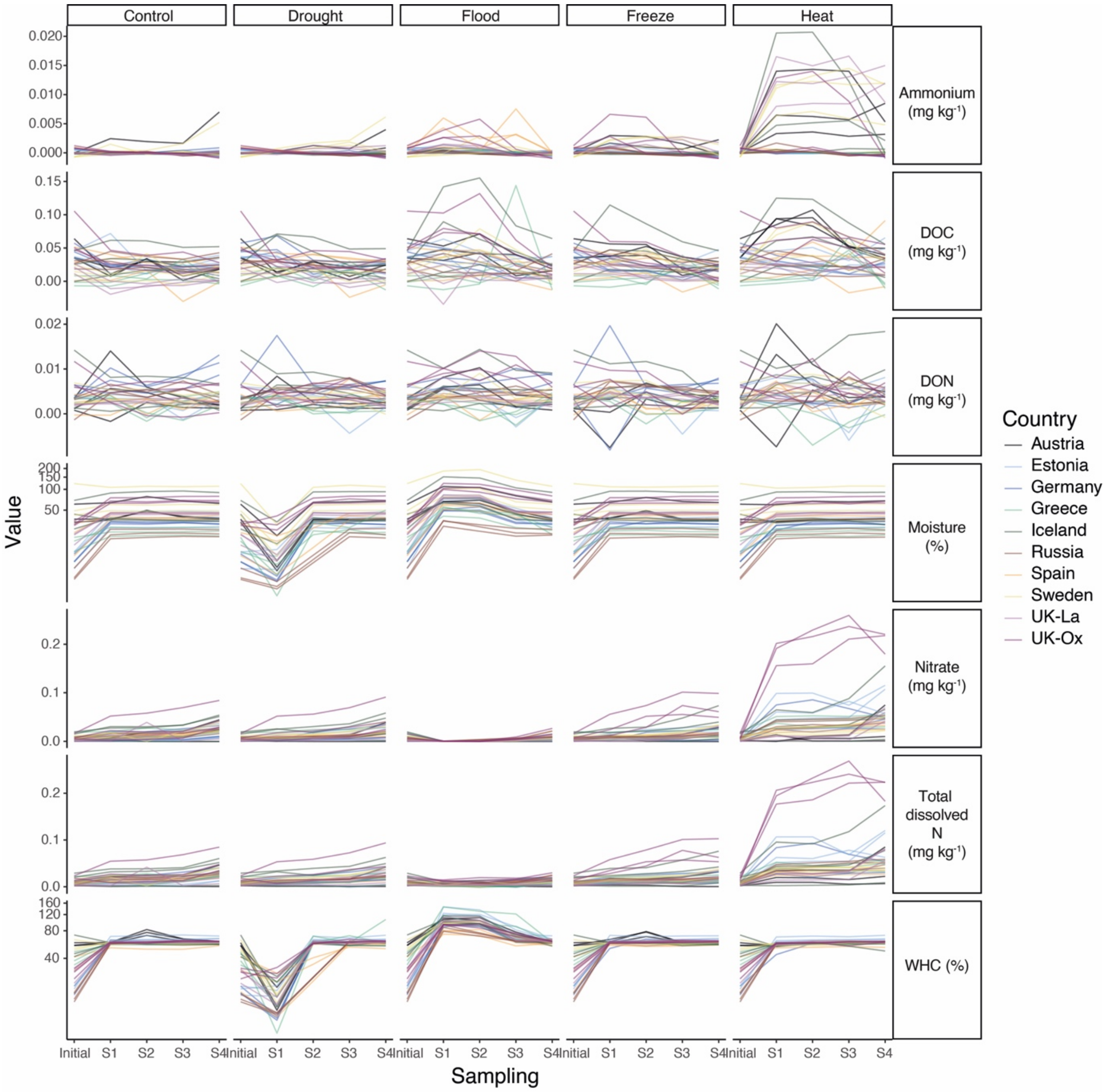

Supplementary Figure 1. Soil properties of soils from different sites in response to the different disturbances over time. Laboratory measurements of key soil properties in the initial samples and four subsequent time points in control and disturbed samples (*N* = 5,180 individual measurements, see Fig. 1 for timings). Lines link measurements of soils from the same sites, the colours of which indicate countries as in Fig. 1. Note the pseudo-logarithmic scale which includes zero, but is logarithmic at higher values. Further details of the underlying data are available at the Figshare site: https://doi.org/ngfr

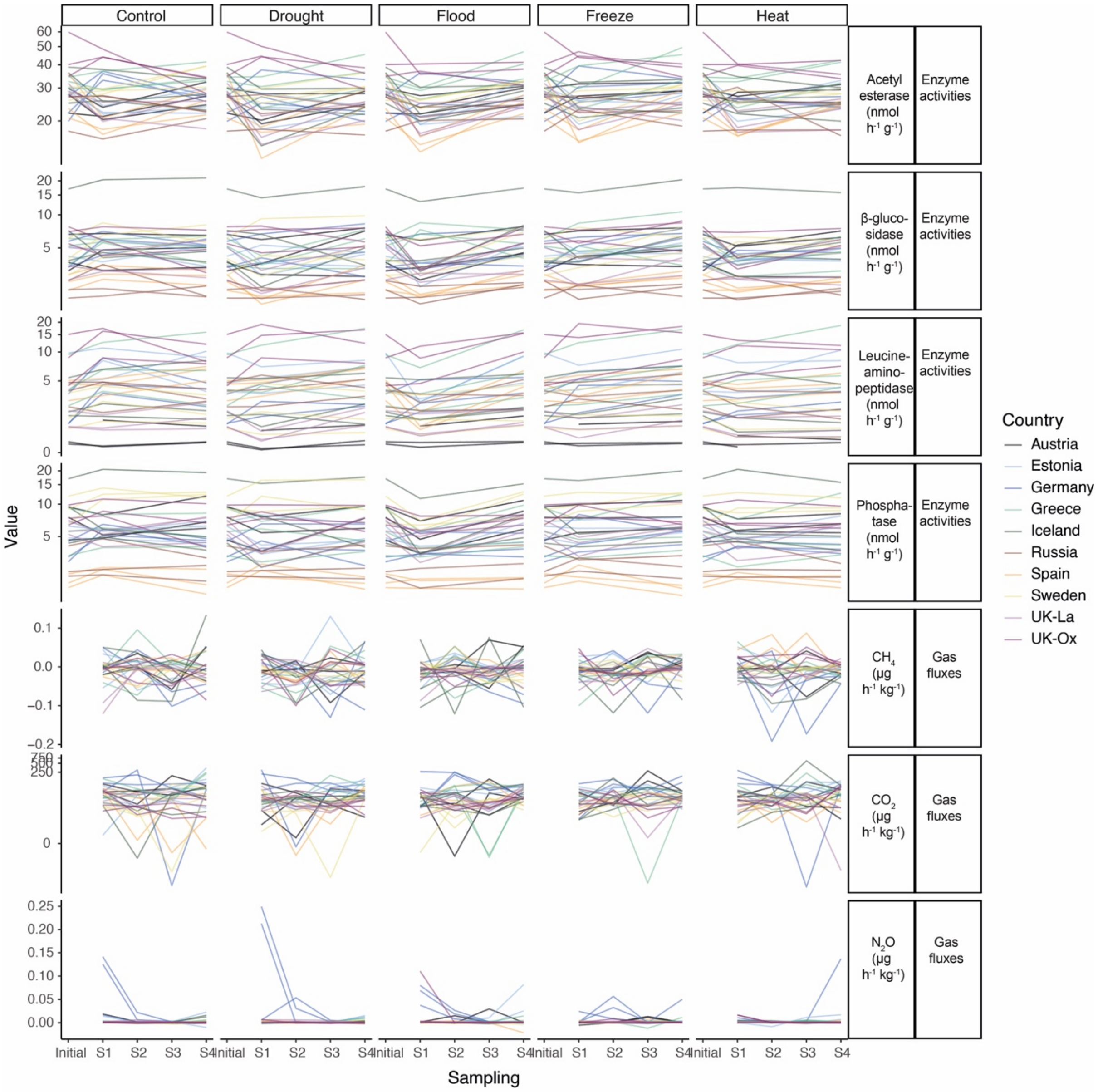

Supplementary Figure 2. Enzyme activities and gas fluxes of soils from different sites in response to the different disturbances over time. Laboratory measurements of enzymes and gas fluxes in the initial samples and up to four subsequent time points in control and disturbed samples (*N* = 3,404 individual measurements see Fig. 1 for timings). Lines link measurements of soils from the same sites, the colours of which indicate countries as in Fig. 1. Note the pseudo-logarithmic scale which includes zero, but is logarithmic at higher values. Further details of the underlying data are available at the Figshare site: https://doi.org/ngfr

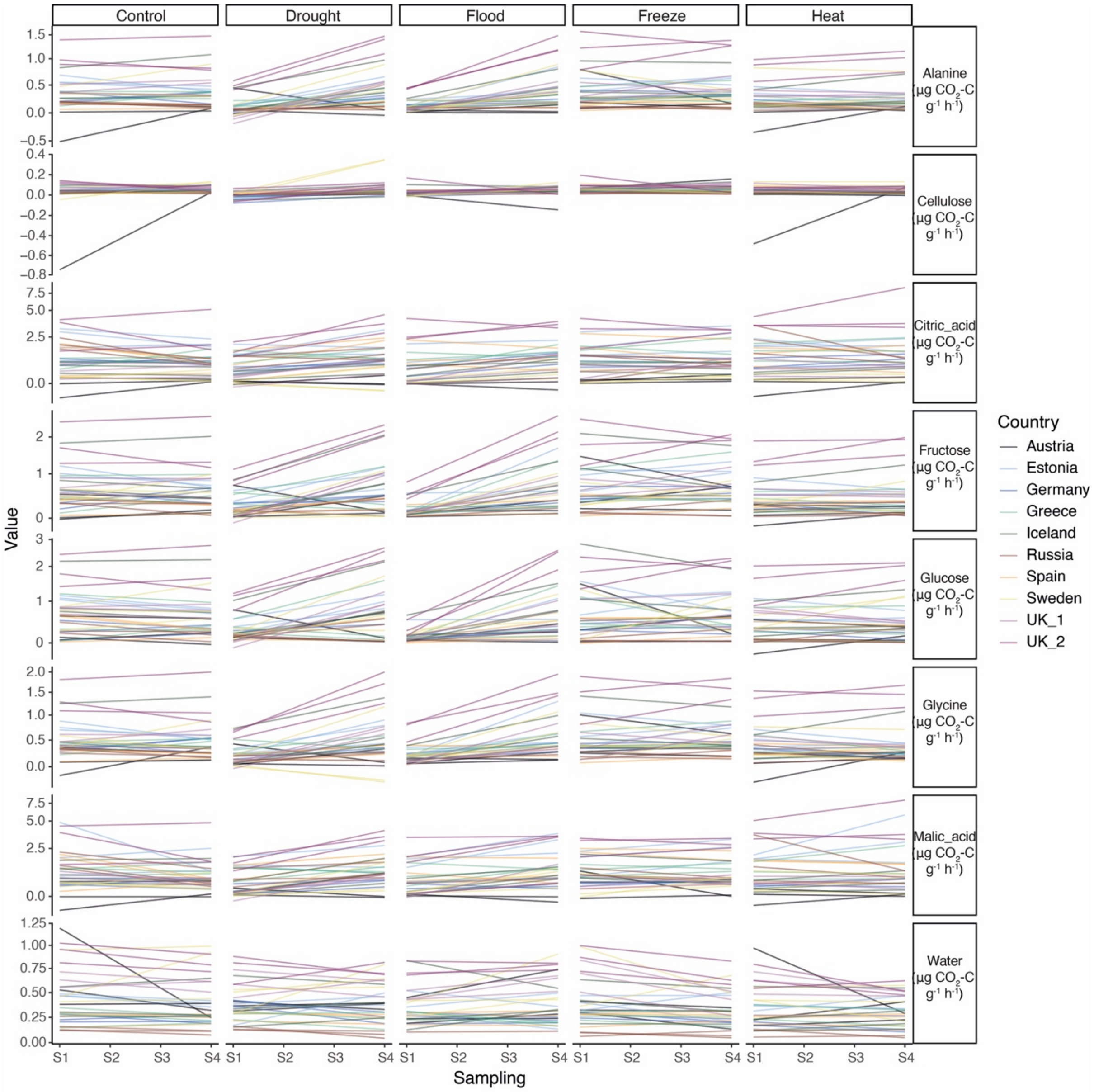

Supplementary Figure 3. Substrate-induced respiration of soils from different sites in response to the different disturbances over time. Laboratory MicroResp measurements at the S1 and S4 time points in control and disturbed samples (*N* = 2,373 individual measurements, see Fig. 1 for timings). Lines link measurements of soils from the same sites, the colours of which indicate countries as in Fig. 1. Note the pseudo-logarithmic scale which includes zero, but is logarithmic at higher values. Further details of the underlying data are available at the Figshare site: https://doi.org/ngfr

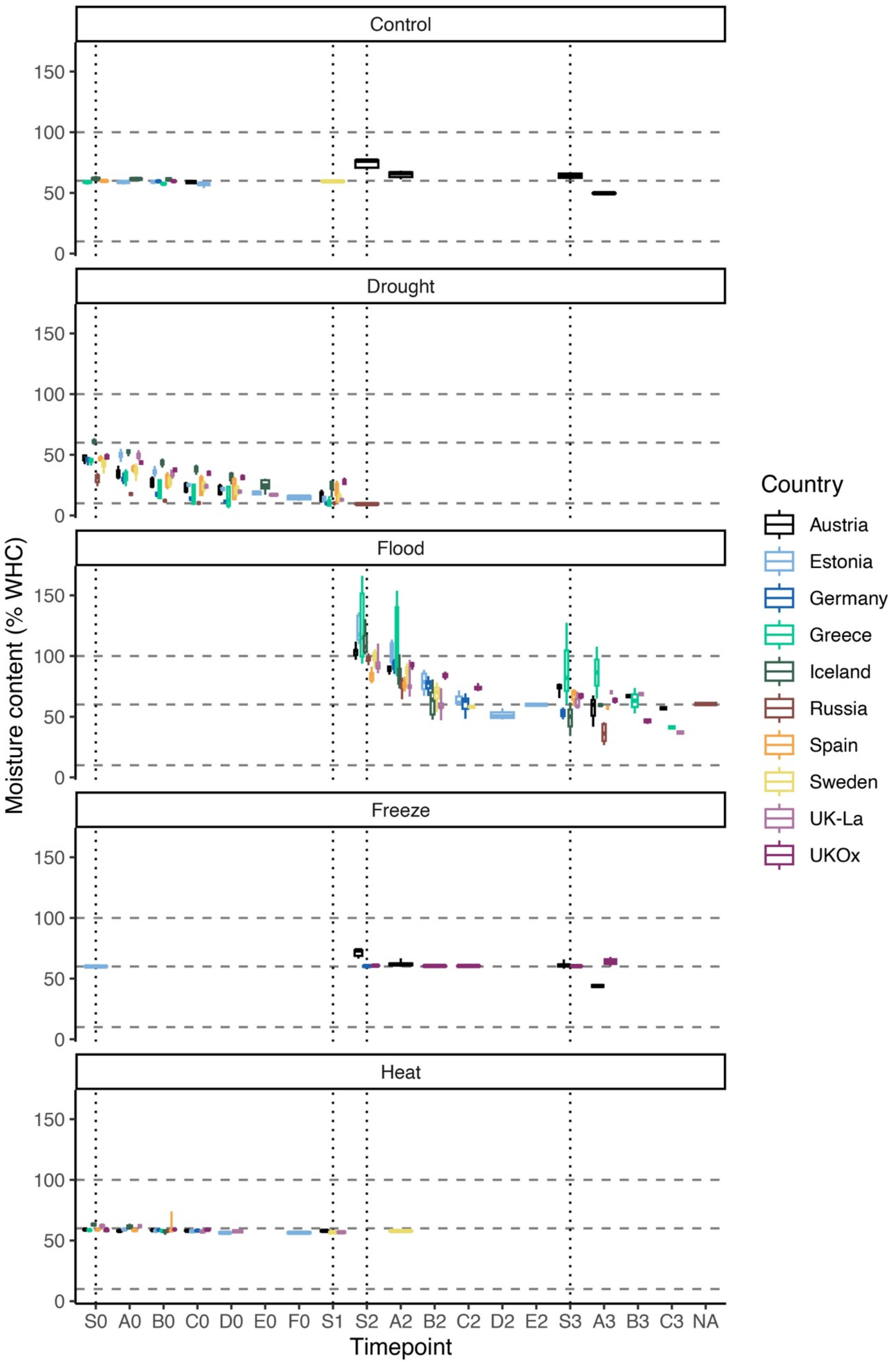

Supplementary Figure 4. Moisture dynamics. Soil moisture content as a percentage of the soil water holding capacity (WHC), through the experiment (*N* = 1,284 individual

measurements). 10%, 60% and 100% of WHC are indicated by horizontal dashes. Vertical dotted lines indicate the start of the perturbation (S0), the end of the perturbation (S1), one day after the end of the perturbation (S2) and one week after the end of the perturbation (S3). Other measurements were taken every 2-3 days for drought and flood treatments through the disturbance and recovery periods respectively (Fig. 1b) and are simply labelled sequentially A0-F0 for the period following S0 and similarly starting with A2 and A3 following S2 and S3 respectively. Colours represent countries as in Fig. 1. Box plots show the full range (whiskers) and the interquartile range (box) across sites within a treatment at a particular timepoint. This includes both different sites from the same country and different pots from the same site destined to be sampled at different time points (i.e. up to 4 pots of each soil in the period before S1, but only one pot following S3 when the S1, S2 and S3 pots had already been sampled). Note that for treatments/ periods during which weights were kept constant, e.g. during freezing and flood, and during recovery when 60% WHC was reached, typically weights were not recorded at each watering date.
