# Supplementary material for: Soil microbiomes show consistent and predictable responses to extreme events
Source: Nature. 2024 Nov 27;636(8043):690–6. doi: 10.1038/s41586-024-08185-3 (PMC11655354; doi:10.1038/s41586-024-08185-3)
Supplement: Supplementary file 2 — Reporting Summary [file 41586_2024_8185_MOESM2_ESM.pdf]

nature portfolio

Corresponding author(s): Franciska de Vries

Last updated by author(s): Sep 12, 2024

Reporting Summary

Nature Portfolio wishes to improve the reproducibility of the work that we publish. This form provides structure for consistency and transparency in reporting. For further information on Nature Portfolio policies, see our Editorial Policies and the Editorial Policy Checklist.

Statistics

For all statistical analyses, confirm that the following items are present in the figure legend, table legend, main text, or Methods section.

n/a	Confirmed	
☐	☒	The exact sample size (*n*) for each experimental group/condition, given as a discrete number and unit of measurement
☐	☒	A statement on whether measurements were taken from distinct samples or whether the same sample was measured repeatedly
☐	☒	The statistical test(s) used AND whether they are one- or two-sided<br>*Only common tests should be described solely by name; describe more complex techniques in the Methods section.*
☐	☒	A description of all covariates tested
☐	☒	A description of any assumptions or corrections, such as tests of normality and adjustment for multiple comparisons
☐	☒	A full description of the statistical parameters including central tendency (e.g. means) or other basic estimates (e.g. regression coefficient) AND variation (e.g. standard deviation) or associated estimates of uncertainty (e.g. confidence intervals)
☐	☒	For null hypothesis testing, the test statistic (e.g. *F*, *t*, *r*) with confidence intervals, effect sizes, degrees of freedom and *P* value noted<br>*Give P values as exact values whenever suitable.*
☒	☐	For Bayesian analysis, information on the choice of priors and Markov chain Monte Carlo settings
☐	☒	For hierarchical and complex designs, identification of the appropriate level for tests and full reporting of outcomes
☐	☒	Estimates of effect sizes (e.g. Cohen's *d*, Pearson's *r*), indicating how they were calculated

Our web collection on statistics for biologists contains articles on many of the points above.

Software and code

Policy information about availability of computer code

Data collection	Data collection not involving software
Data analysis	Most analysis was carried out with R version 4.2, using the following key packages:<br>broom.mixed 0.2, caper 1.0, car 3.1, cowplot 1.1, DADA2 1.24, DECIPHER 2.24, eulerr 7.0, fs 1.6, ggordiplots 0.4, ggplot 3.3, ggrepel 0.9, Hmisc 5.1, lme4 1.1, lmerTest 3.1, magrittr 2.0, MASS 7.3, multcomp 1.4, nlme 3.1, pacman 0.5, pals 1.9, pdp 0.8, pracma 2.4, randomForest 4.7, RasperGade16S 0.0, RColorBrewer 1.1, seqinr 4.2, tidyverse 2.0, vegan 2.6<br>In addition, the following stand-alone software was utilised<br>FastTree (2.1)<br>Diamond (0.8 and 2.0)<br>BLAST (2.13)

For manuscripts utilizing custom algorithms or software that are central to the research but not yet described in published literature, software must be made available to editors and reviewers. We strongly encourage code deposition in a community repository (e.g. GitHub). See the Nature Portfolio guidelines for submitting code & software for further information.

Data

Policy information about availability of data

All manuscripts must include a data availability statement. This statement should provide the following information, where applicable:

- Accession codes, unique identifiers, or web links for publicly available datasets
- A description of any restrictions on data availability
- For clinical datasets or third party data, please ensure that the statement adheres to our policy

All raw sequence files are deposited in the European Nucleotide Archive at project accession PRJEB52753, with paired fastq files for each sample under Run accessions ERR9712737-ERR9713356 (16S amplicons); ERR9713357-ERR9713976 (ITS amplicons), and ERR9924623-ERR9924930 (whole genome metagenomes). Derived data and all code are available at https://doi.org/ngfr with explicit accessions for the raw data at ENA. Climatic data are available from WorldClim.org

Human research participants

Policy information about studies involving human research participants and Sex and Gender in Research.

Reporting on sex and gender	NA
Population characteristics	NA
Recruitment	NA
Ethics oversight	NA

Note that full information on the approval of the study protocol must also be provided in the manuscript.

Field-specific reporting

Please select the one below that is the best fit for your research. If you are not sure, read the appropriate sections before making your selection.

☐ Life sciences ☐ Behavioural & social sciences ☒ Ecological, evolutionary & environmental sciences

For a reference copy of the document with all sections, see nature.com/documents/nr-reporting-summary-flat.pdf

Ecological, evolutionary & environmental sciences study design

All studies must disclose on these points even when the disclosure is negative.

Study description	The experiment was a factorial design between treatment (a factor with five levels) and sampling time (a factor with four levels, S1-S4, though some analyses only used S1 and S4 and there was also an Initial sample for each soil). A nested sampling approach was taken with three replicate sites sampled within each of 10 countries. A single pot of soil was utilised for each replicate site at each sampling time in each treatment.
Research sample	Samples from each replicate site comprised 4 pooled 3cm diameter x 15cm long soil cores
Sampling strategy	For a fixed number of samples to be analysed, the strategy was to maximise the diversity of soils sampled. This results in the design with a single sample for each soil/treatment/time combination.
Data collection	Laboratory data collection on soils carried out in Manchester by ON, CW and HL with sequencing data collected by CGR, Liverpool and the UK Centre for Ecology and Hydrology (RIG, TG and BJ)
Timing and spatial scale	Samples were collected when the average temperature of the location was closest to 18 °C, i.e. in spring for southern locations, and after the snow melt, in summer, for the northern locations. In May 2018, we collected soil from Russia, Greece, and Estonia and Spain, followed by Germany and Oxford in June, Austria and Iceland in July, and Lancaster and Sweden in August. Three spatial scales were used – European country-scale shown in Supplementary Fig. 1; replicate sites within countries, separated by 0.05 – 11.76 km; cores within sites, taken from four random points within seven 1 m x 1 m plots were arranged at least 5 m apart.
Data exclusions	Two replicate sites from Spain (sites 1 and 3; n = 42) were excluded from the analyses due to low DNA yield and poor recovery of reads particularly for the prokaryotic amplicons
Reproducibility	The clustering of replicate sites in Fig. 1 demonstrates reproducibility.
Randomization	Location of soil cores within plots were randomised; assignment of soil samples to treatments was done randomly; physical locations of microcosms on trays was randomised.

Blinding	On samples' arrival in Manchester all researchers were blinded to the identity of the samples by making their only identity the unique random numbers allocated to them during the randomisation.

Did the study involve field work? ☒ Yes ☐ No

Field work, collection and transport

Field conditions	Conditions at each site are given in the data file associated with the samples
Location	Locations, including latitude and longitude are given in the data file associated with the samples
Access & import/export	Soils were imported under DEFRA plant health licence 116619/374208/0 Valid from 01 January 2019 to 31 December 2019
Disturbance	Clearing of obstructions at the sites was limited to the 7 single square metre plots.

Reporting for specific materials, systems and methods

We require information from authors about some types of materials, experimental systems and methods used in many studies. Here, indicate whether each material, system or method listed is relevant to your study. If you are not sure if a list item applies to your research, read the appropriate section before selecting a response.

Materials & experimental systems

n/a	Involved in the study	
☒	☐	Antibodies
☒	☐	Eukaryotic cell lines
☒	☐	Palaeontology and archaeology
☒	☐	Animals and other organisms
☒	☐	Clinical data
☒	☐	Dual use research of concern

Methods

n/a	Involved in the study	
☒	☐	ChIP-seq
☒	☐	Flow cytometry
☒	☐	MRI-based neuroimaging
