# Supplementary material for: Soil microbiomes show consistent and predictable responses to extreme events
Source: Nature. 2024 Nov 27;636(8043):690–6. doi: 10.1038/s41586-024-08185-3 (PMC11655354; doi:10.1038/s41586-024-08185-3)
Supplement: Supplementary file 4 — This multipage PDF file contains the functional impacts of our simulated extreme events at all functional levels. One plot is given for each of the highest functional levels (Fig. 3). Plots are as in Fig. 3a, except that models reported are fitted for the finest scale of function (individual proteins, n = 280 metagenomic samples), each shown by a row of four points. Only proteins that show significant variation among treatments (P ≤ 0.05, two-tailed test, following false discovery rate correction for multiple models) are plotted. The number of such significantly varying proteins and the total number of proteins are given following the name of each higher-level function on the right; if that higher-level function as a whole varies significantly (P ≤ 0.05, two-tailed test, following false discovery rate correction for multiple models), that name is shown in bold. Each significant change in a protein (P ≤ 0.05, two-tailed Dunnett’s test, for a particular disturbance versus control accounting for multiple testing within a mixed-effects model) has a filled point, as in Fig. 3, and contributes to the background colour corresponding to its higher-level function in a particular treatment; therefore, more intense background colours indicate more proteins that have significantly changed and purple shading indicates some proteins within that function significantly increasing relative to control, with some significantly decreasing. [file 41586_2024_8185_MOESM4_ESM.pdf]

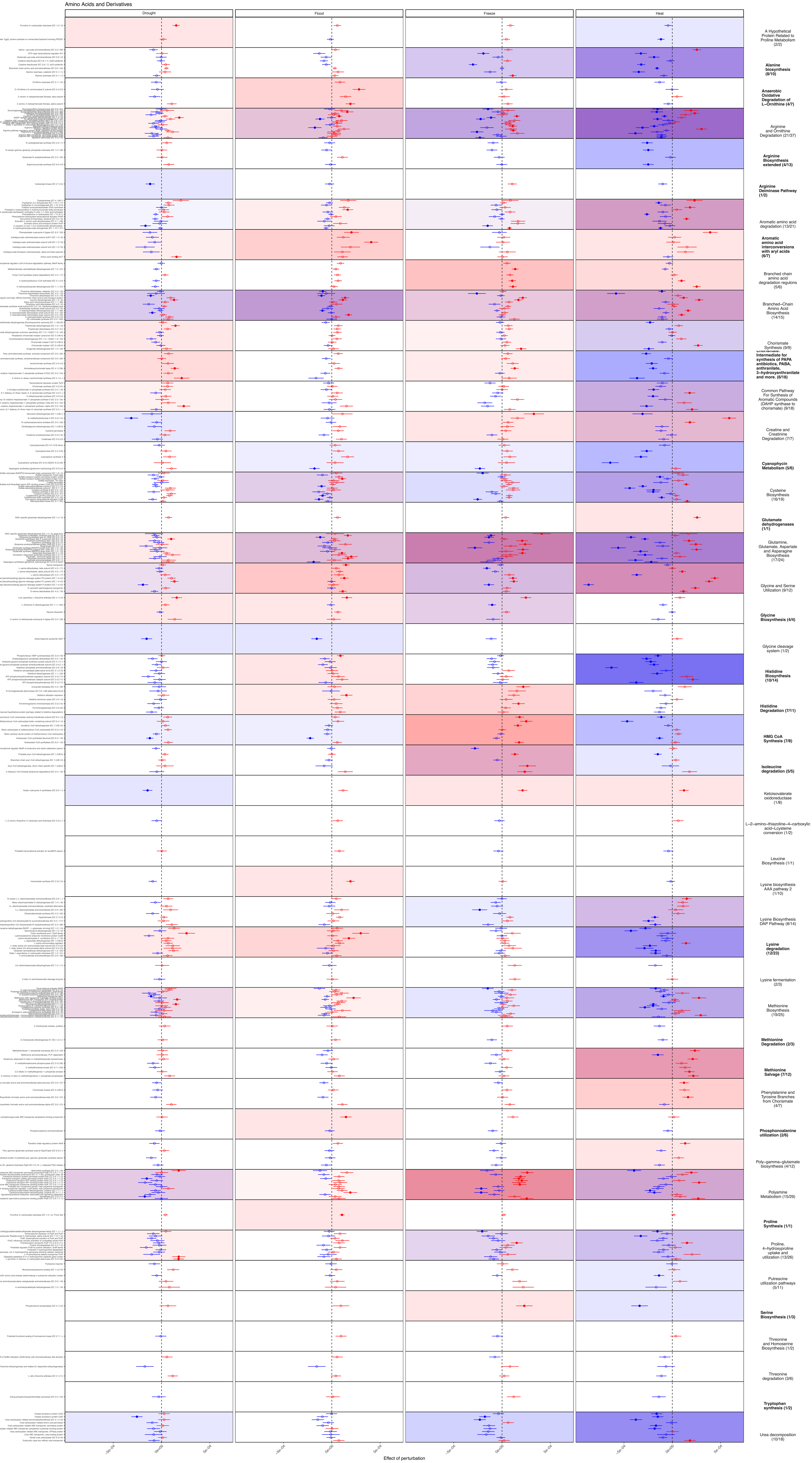
Amino Acids and Derivatives
Drought
Flood
Freeze
Heat
A Hypothetical Protein Related to Proline Metabolism (2/2)
Alanine biosynthesis (8/10)
Anaerobic Oxidative Degradation of L−Ornithine (4/7)
Arginine and Ornithine Degradation (21/37)
Arginine Biosynthesis extended (4/13)
Arginine Deiminase Pathway (1/2)
Aromatic amino acid degradation (13/21)
Aromatic amino acid interconversions with aryl acids (6/7)
Branched chain amino acid degradation regulons (5/6)
Branched−Chain Amino Acid Biosynthesis (14/15)
Chorismate Synthesis (9/9)
Chorismate: Intermediate for synthesis of PAPA antibiotics, PABA, anthranilate, 3−hydroxyanthranilate and more. (6/18)
Common Pathway For Synthesis of Aromatic Compounds (DAHP synthase to chorismate) (9/18)
Creatine and Creatinine Degradation (7/7)
Cyanophycin Metabolism (5/6)
Cysteine Biosynthesis (16/19)
Glutamate dehydrogenases (1/1)
Glutamine, Glutamate, Aspartate and Asparagine Biosynthesis (17/24)
Glycine and Serine Utilization (9/12)
Glycine Biosynthesis (4/4)
Glycine cleavage system (1/2)
Histidine Biosynthesis (10/14)
Histidine Degradation (7/11)
HMG CoA Synthesis (7/8)
Isoleucine degradation (5/5)
Ketoisovalerate oxidoreductase (1/8)
L−2−amino−thiazoline−4−carboxylic acid−Lcysteine conversion (1/2)
Leucine Biosynthesis (1/1)
Lysine biosynthesis AAA pathway 2 (1/10)
Lysine Biosynthesis DAP Pathway (8/14)
Lysine degradation (12/23)
Lysine fermentation (2/3)
Methionine Biosynthesis (19/25)
Methionine Degradation (2/3)
Methionine Salvage (7/12)
Phenylalanine and Tyrosine Branches from Chorismate (4/7)
Phosphonoalanine utilization (2/6)
Poly−gamma−glutamate biosynthesis (4/12)
Polyamine Metabolism (15/29)
Proline Synthesis (1/1)
Proline, 4−hydroxyproline uptake and utilization (13/28)
Putrescine utilization pathways (5/11)
Serine Biosynthesis (1/3)
Threonine and Homoserine Biosynthesis (1/2)
Threonine degradation (3/6)
Tryptophan synthesis (1/2)
Urea decomposition (10/18)
−5e−04
0e+00
5e−04
Effect of perturbation

Carbohydrates

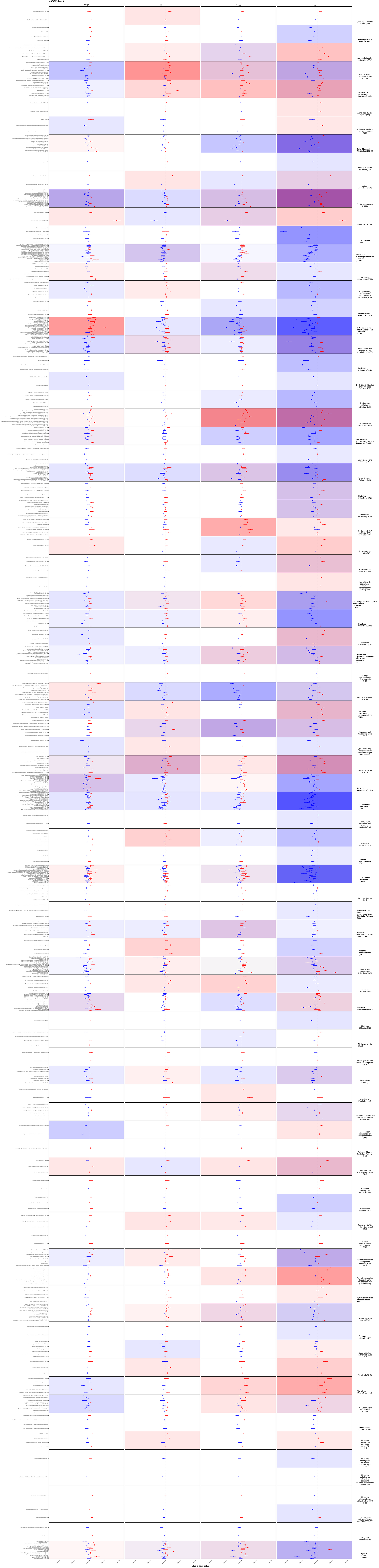

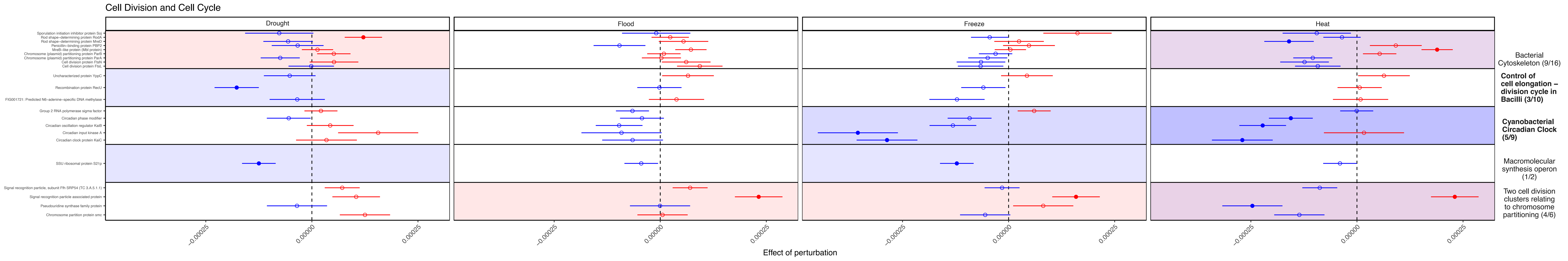

Cell Division and Cell Cycle
Drought
Flood
Freeze
Heat
Sporulation initiation inhibitor protein Soj
Rod shape–determining protein RodA
Rod shape–determining protein MreD
Penicillin–binding protein PBP2
MreB–like protein (Mbl protein)
Chromosome (plasmid) partitioning protein ParB
Chromosome (plasmid) partitioning protein ParA
Cell division protein FtsN
Cell division protein FtsL
Uncharacterized protein YppC
Recombination protein RecU
FIG001721: Predicted N6–adenine–specific DNA methylase
Group 2 RNA polymerase sigma factor
Circadian phase modifier
Circadian oscillation regulator KaiB
Circadian input kinase A
Circadian clock protein KaiC
SSU ribosomal protein S21p
Signal recognition particle, subunit Ffh SRP54 (TC 3.A.5.1.1)
Signal recognition particle associated protein
Pseudouridine synthase family protein
Chromosome partition protein smc
Bacterial Cytoskeleton (9/16)
Control of cell elongation – division cycle in Bacilli (3/10)
Cyanobacterial Circadian Clock (5/9)
Macromolecular synthesis operon (1/2)
Two cell division clusters relating to chromosome partitioning (4/6)
-0.00025
0.00000
0.00025
Effect of perturbation

Cell Wall and Capsule

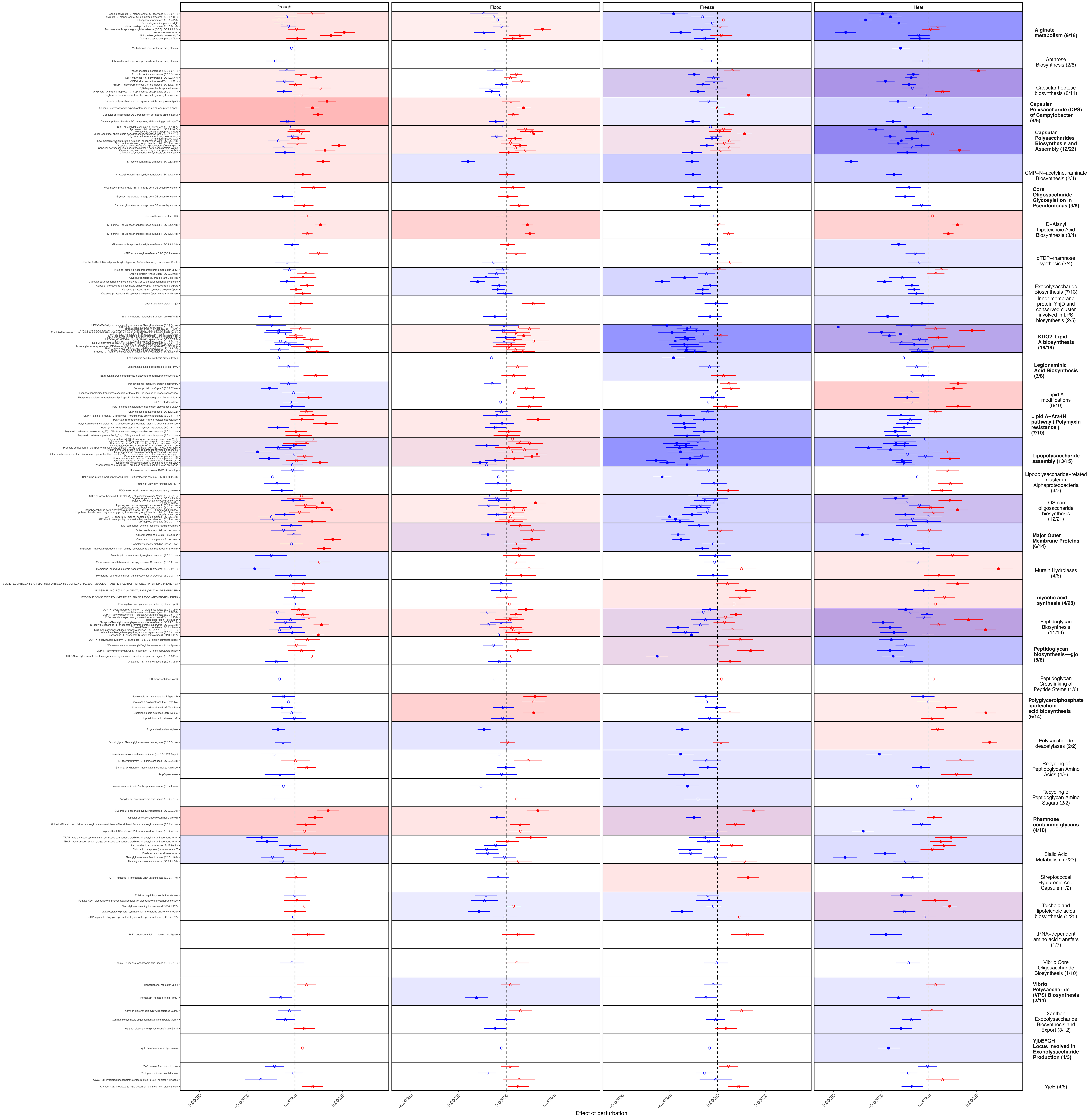

Clustering-based subsystems

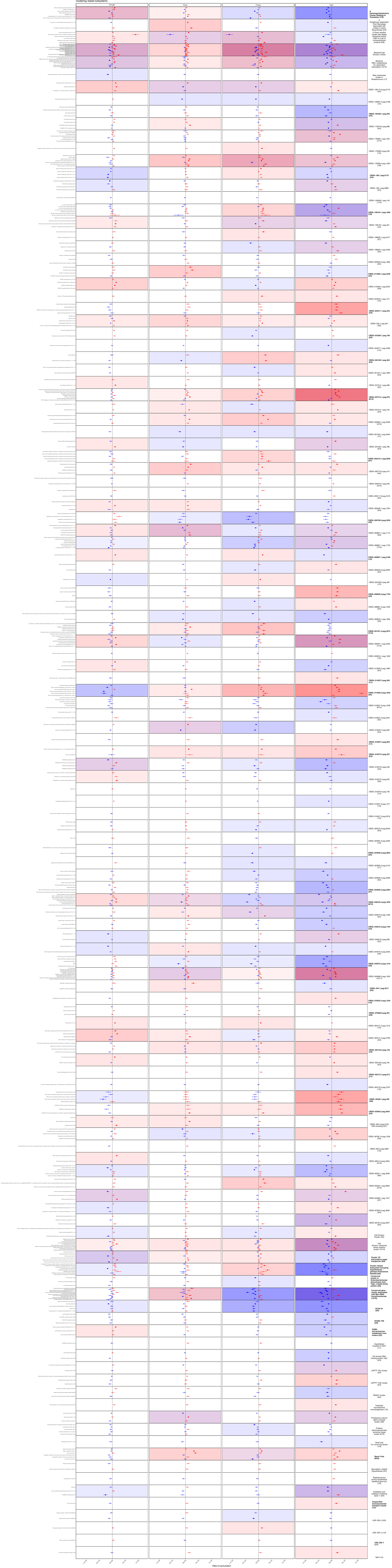

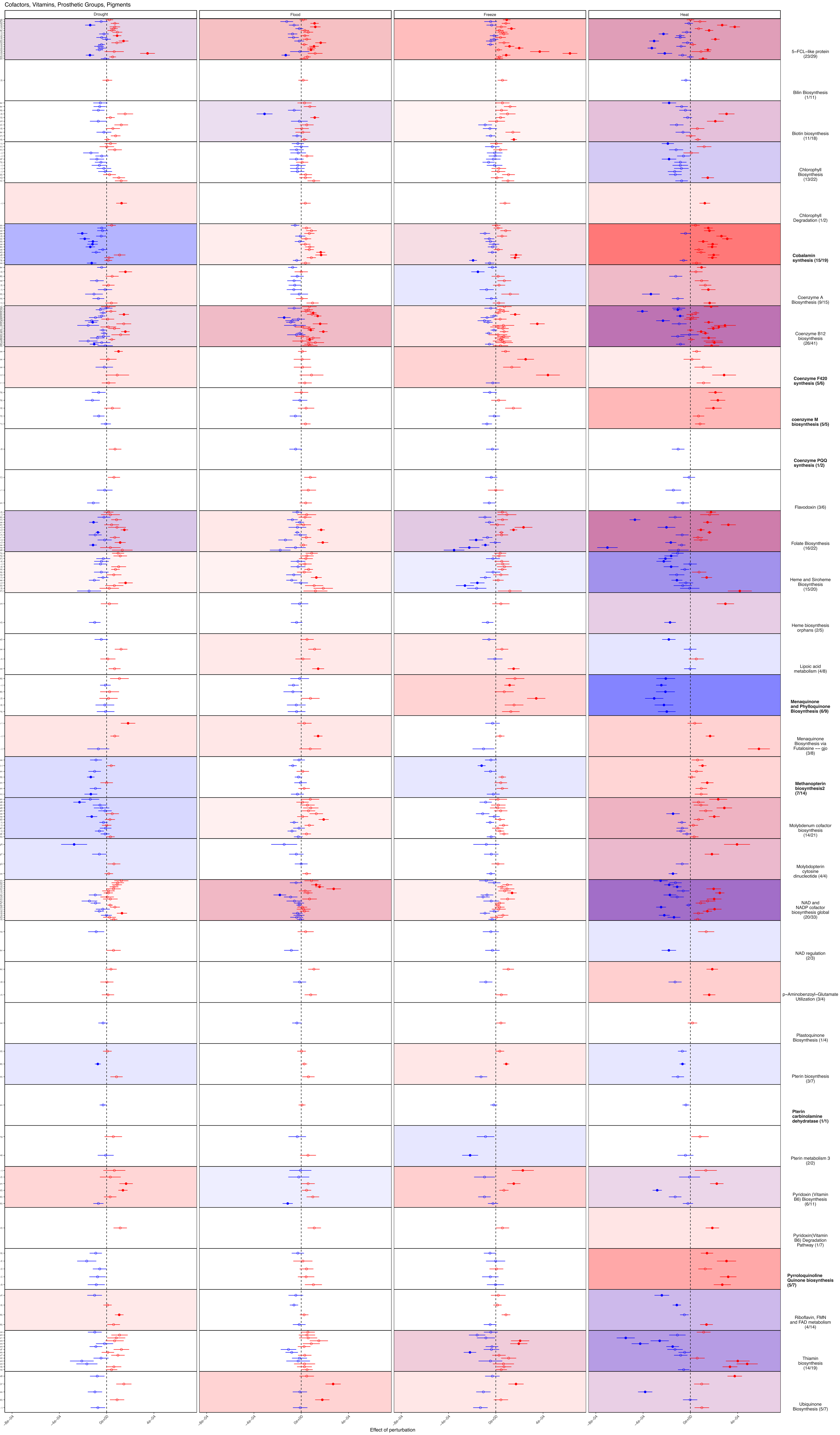

Cofactors, Vitamins, Prosthetic Groups, Pigments
Drought
Flood
Freeze
Heat
5−FCL−like protein (23/29)
Bilin Biosynthesis (1/11)
Biotin biosynthesis (11/18)
Chlorophyll Biosynthesis (13/22)
Chlorophyll Degradation (1/2)
Cobalamin synthesis (15/19)
Coenzyme A Biosynthesis (9/15)
Coenzyme B12 biosynthesis (26/41)
Coenzyme F420 synthesis (5/6)
coenzyme M biosynthesis (5/5)
Coenzyme PQQ synthesis (1/2)
Flavodoxin (3/6)
Folate Biosynthesis (16/22)
Heme and Siroheme Biosynthesis (15/20)
Heme biosynthesis orphans (2/5)
Lipoic acid metabolism (4/8)
Menaquinone and Phylloquinone Biosynthesis (6/9)
Menaquinone Biosynthesis via Futalosine -- gjo (3/8)
Methanopterin biosynthesis2 (7/14)
Molybdenum cofactor biosynthesis (14/21)
Molybdopterin cytosine dinucleotide (4/4)
NAD and NADP cofactor biosynthesis global (20/33)
NAD regulation (2/3)
p−Aminobenzoyl−Glutamate Utilization (3/4)
Plastoquinone Biosynthesis (1/4)
Pterin biosynthesis (3/7)
Pterin carbinolamine dehydratase (1/1)
Pterin metabolism 3 (2/2)
Pyridoxin (Vitamin B6) Biosynthesis (6/11)
Pyridoxin(Vitamin B6) Degradation Pathway (1/7)
Pyrroloquinoline Quinone biosynthesis (5/7)
Riboflavin, FMN and FAD metabolism (4/14)
Thiamin biosynthesis (14/19)
Ubiquinone Biosynthesis (5/7)
−8e−04
−4e−04
0e+00
4e−04
Effect of perturbation

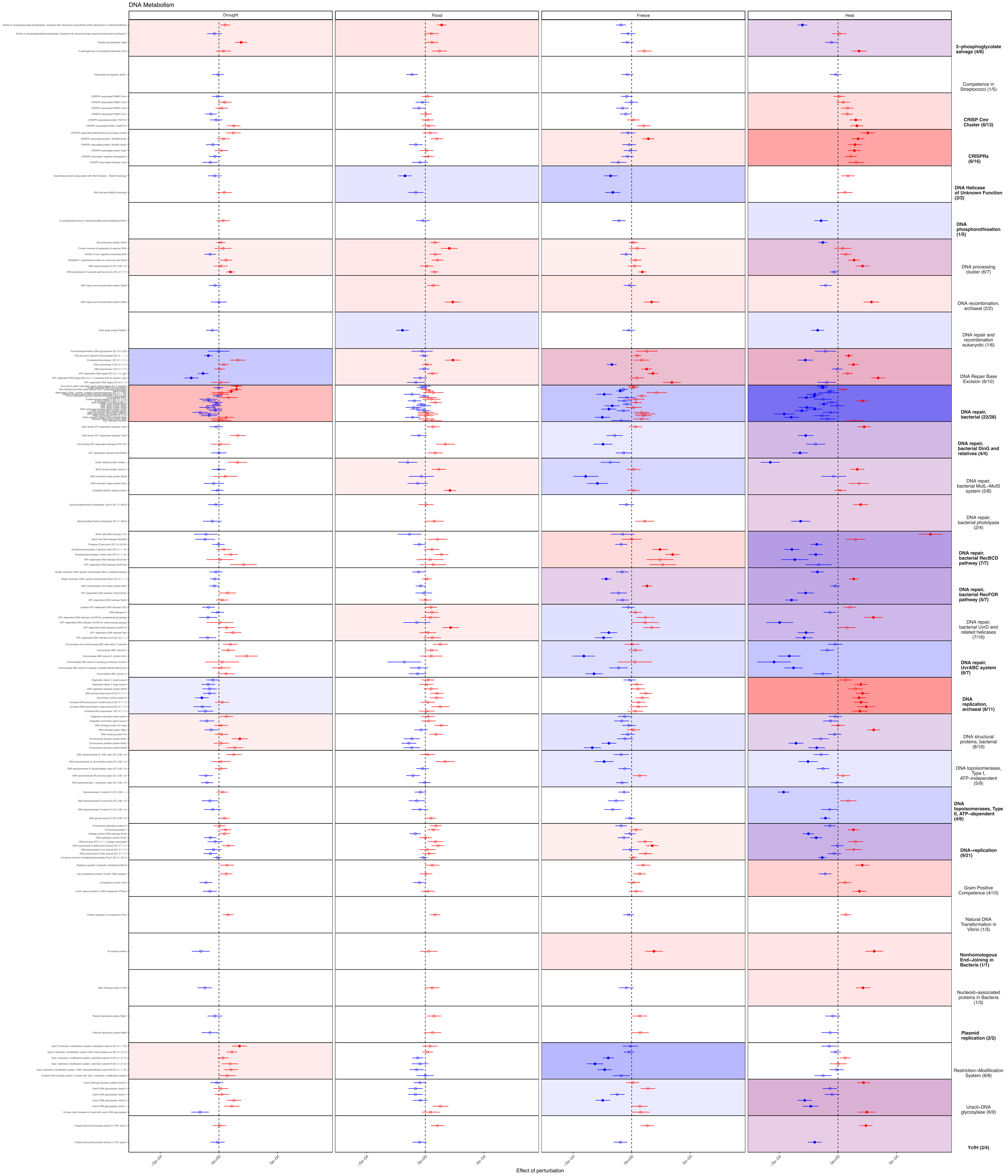

DNA Metabolism
Drought
Flood
Freeze
Heat
2−phosphoglycolate salvage (4/6)
Competence in Streptococci (1/5)
CRISP Cmr Cluster (6/13)
CRISPRs (6/16)
DNA Helicase of Unknown Function (2/2)
DNA phosphorothioation (1/5)
DNA processing cluster (6/7)
DNA recombination, archaeal (2/2)
DNA repair and recombination eukaryotic (1/6)
DNA Repair Base Excision (8/10)
DNA repair, bacterial (22/28)
DNA repair, bacterial DinG and relatives (4/4)
DNA repair, bacterial MutL−MutS system (5/8)
DNA repair, bacterial photolyase (2/4)
DNA repair, bacterial RecBCD pathway (7/7)
DNA repair, bacterial RecFOR pathway (5/7)
DNA repair, bacterial UvrD and related helicases (7/16)
DNA repair, UvrABC system (6/7)
DNA replication, archaeal (8/11)
DNA structural proteins, bacterial (8/10)
DNA topoisomerases, Type I, ATP−independent (5/9)
DNA topoisomerases, Type II, ATP−dependent (4/6)
DNA−replication (9/21)
Gram Positive Competence (4/10)
Natural DNA Transformation in Vibrio (1/3)
Nonhomologous End−Joining in Bacteria (1/1)
Nucleoid−associated proteins in Bacteria (1/3)
Plasmid replication (2/2)
Restriction−Modification System (6/8)
Uracil−DNA glycosylase (6/9)
YcfH (2/4)
−5e−04
0e+00
5e−04
Effect of perturbation

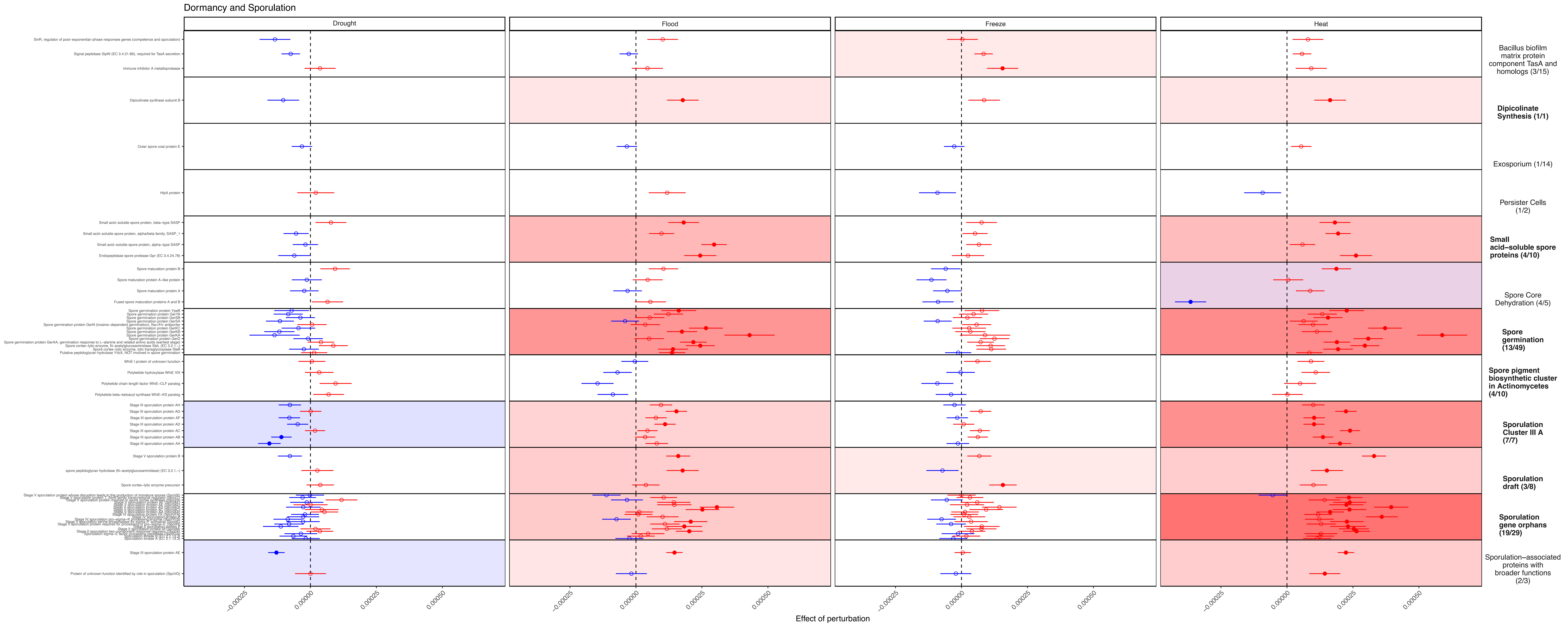
Dormancy and Sporulation
Drought
Flood
Freeze
Heat
SinR, regulator of post−exponential−phase responses genes (competence and sporulation)
Signal peptidase SipW (EC 3.4.21.89), required for TasA secretion
Immune inhibitor A metalloprotease
Dipicolinate synthase subunit B
Outer spore coat protein E
HipA protein
Small acid−soluble spore protein, beta−type SASP
Small acid−soluble spore protein, alpha/beta family, SASP_1
Small acid−soluble spore protein, alpha−type SASP
Endopeptidase spore protease Gpr (EC 3.4.24.78)
Spore maturation protein B
Spore maturation protein A−like protein
Spore maturation protein A
Fused spore maturation proteins A and B
WhiE I protein of unknown function
Polyketide hydroxylase WhiE VIII
Polyketide chain length factor WhiE−CLF paralog
Polyketide beta−ketoacyl synthase WhiE−KS paralog
Stage III sporulation protein AH
Stage III sporulation protein AG
Stage III sporulation protein AF
Stage III sporulation protein AD
Stage III sporulation protein AC
Stage III sporulation protein AB
Stage III sporulation protein AA
Stage V sporulation protein B
spore peptidoglycan hydrolase (N−acetylglucosaminidase) (EC 3.2.1.−)
Spore cortex−lytic enzyme precursor
Stage III sporulation protein AE
Protein of unknown function identified by role in sporulation (SpoVG)
Bacillus biofilm matrix protein component TasA and homologs (3/15)
Dipicolinate Synthesis (1/1)
Exosporium (1/14)
Persister Cells (1/2)
Small acid−soluble spore proteins (4/10)
Spore Core Dehydration (4/5)
Spore germination (13/49)
Spore pigment biosynthetic cluster in Actinomycetes (4/10)
Sporulation Cluster III A (7/7)
Sporulation draft (3/8)
Sporulation gene orphans (19/29)
Sporulation−associated proteins with broader functions (2/3)
−0.00025
0.00000
0.00025
0.00050
Effect of perturbation

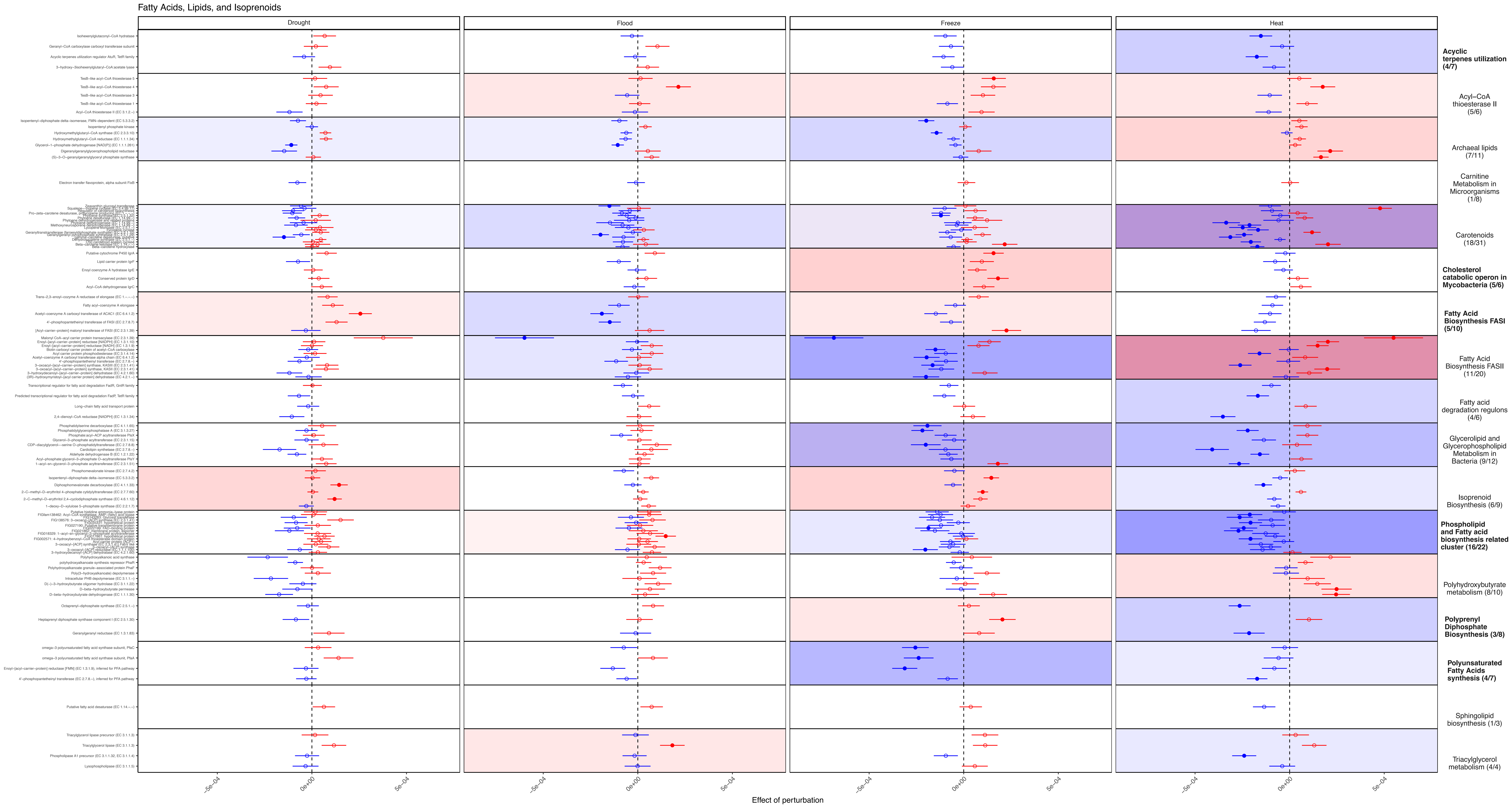
Fatty Acids, Lipids, and Isoprenoids
Drought
Flood
Freeze
Heat
Acyclic terpenes utilization (4/7)
Acyl–CoA thioesterase II (5/6)
Archaeal lipids (7/11)
Carnitine Metabolism in Microorganisms (1/8)
Carotenoids (18/31)
Cholesterol catabolic operon in Mycobacteria (5/6)
Fatty Acid Biosynthesis FASI (5/10)
Fatty Acid Biosynthesis FASII (11/20)
Fatty acid degradation regulons (4/6)
Glycerolipid and Glycerophospholipid Metabolism in Bacteria (9/12)
Isoprenoid Biosynthesis (6/9)
Phospholipid and Fatty acid biosynthesis related cluster (16/22)
Polyhydroxybutyrate metabolism (8/10)
Polyprenyl Diphosphate Biosynthesis (3/8)
Polyunsaturated Fatty Acids synthesis (4/7)
Sphingolipid biosynthesis (1/3)
Triacylglycerol metabolism (4/4)
−5e−04
0e+00
5e−04
Effect of perturbation

Iron acquisition and metabolism

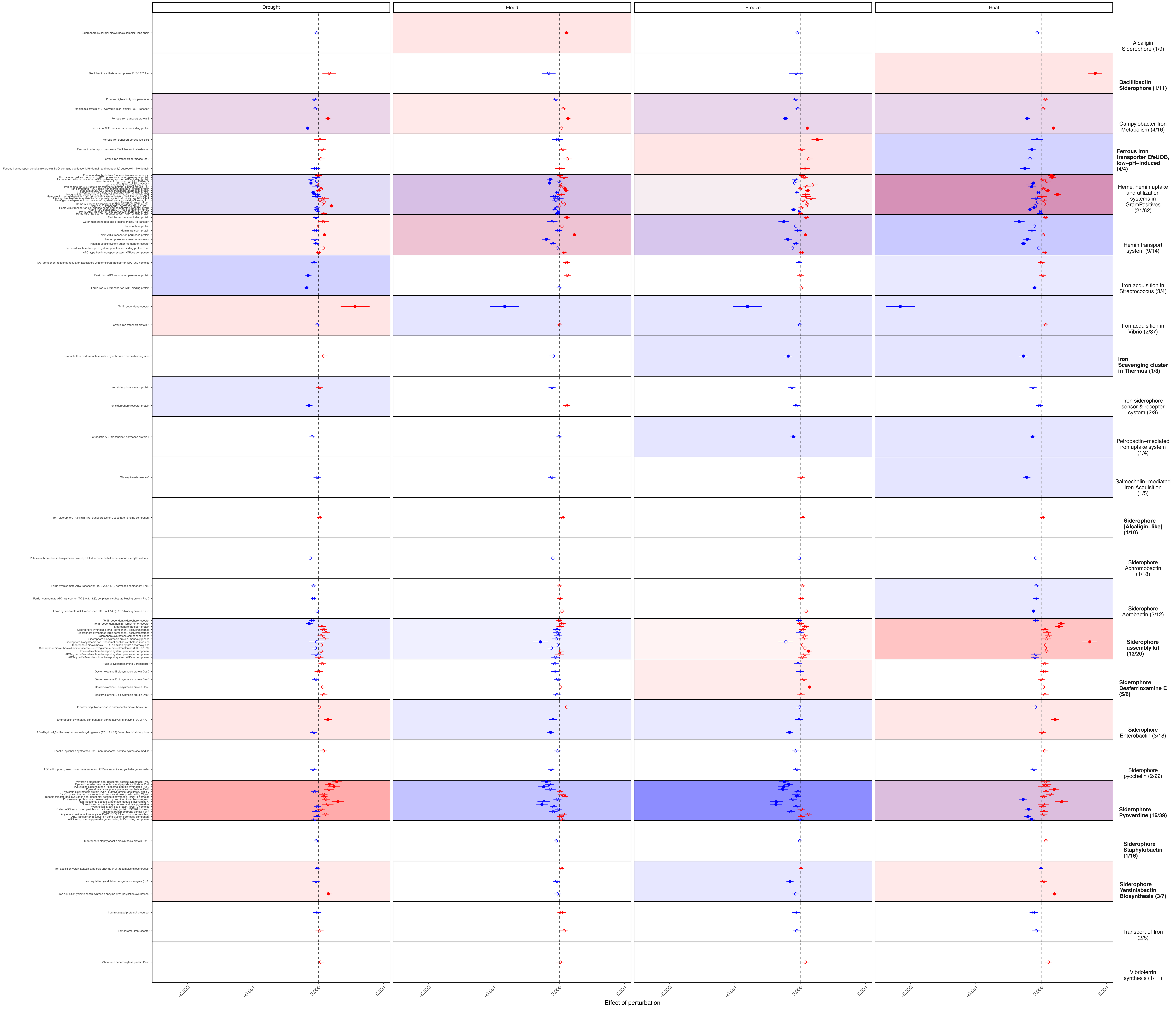

Membrane Transport

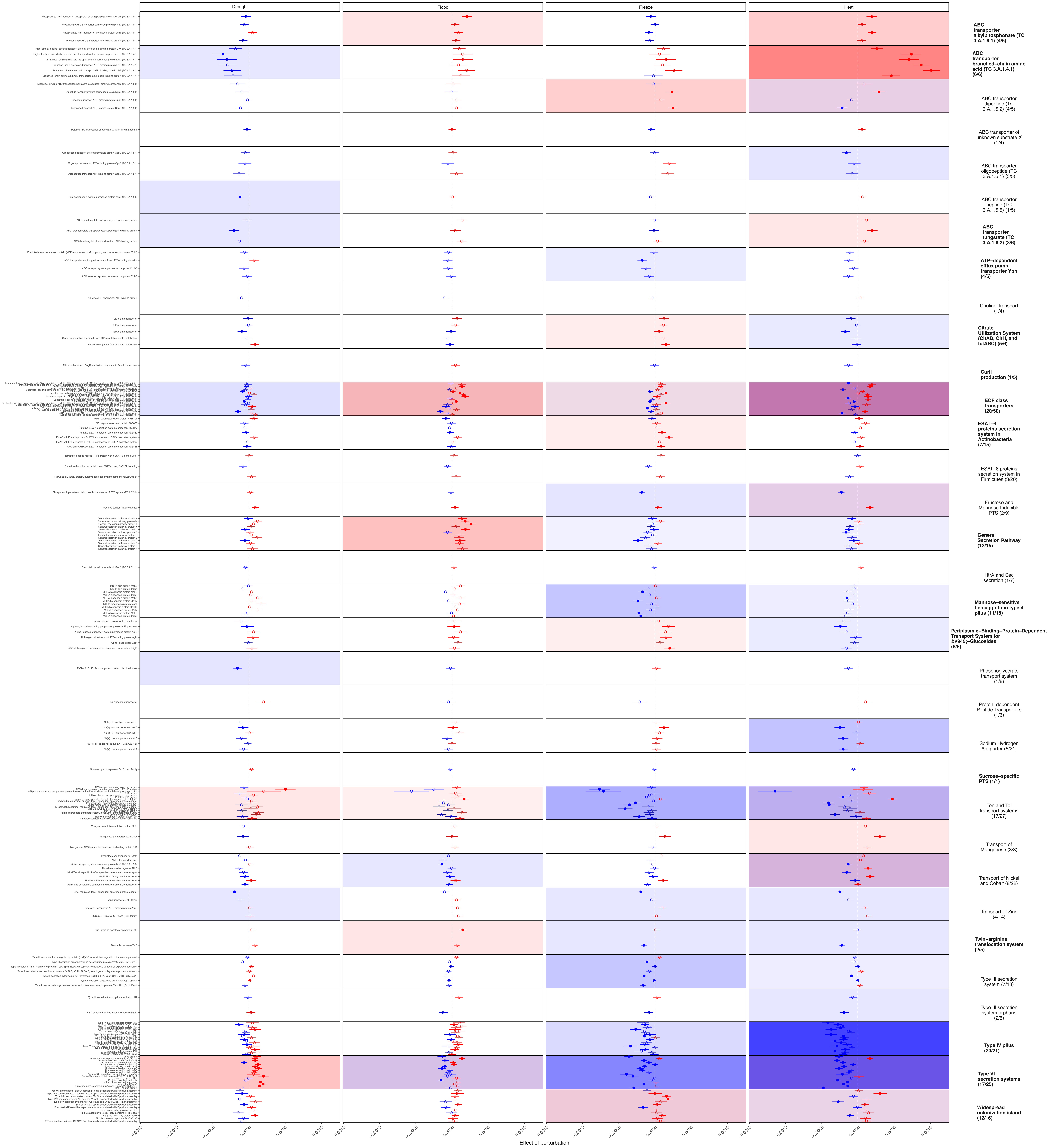

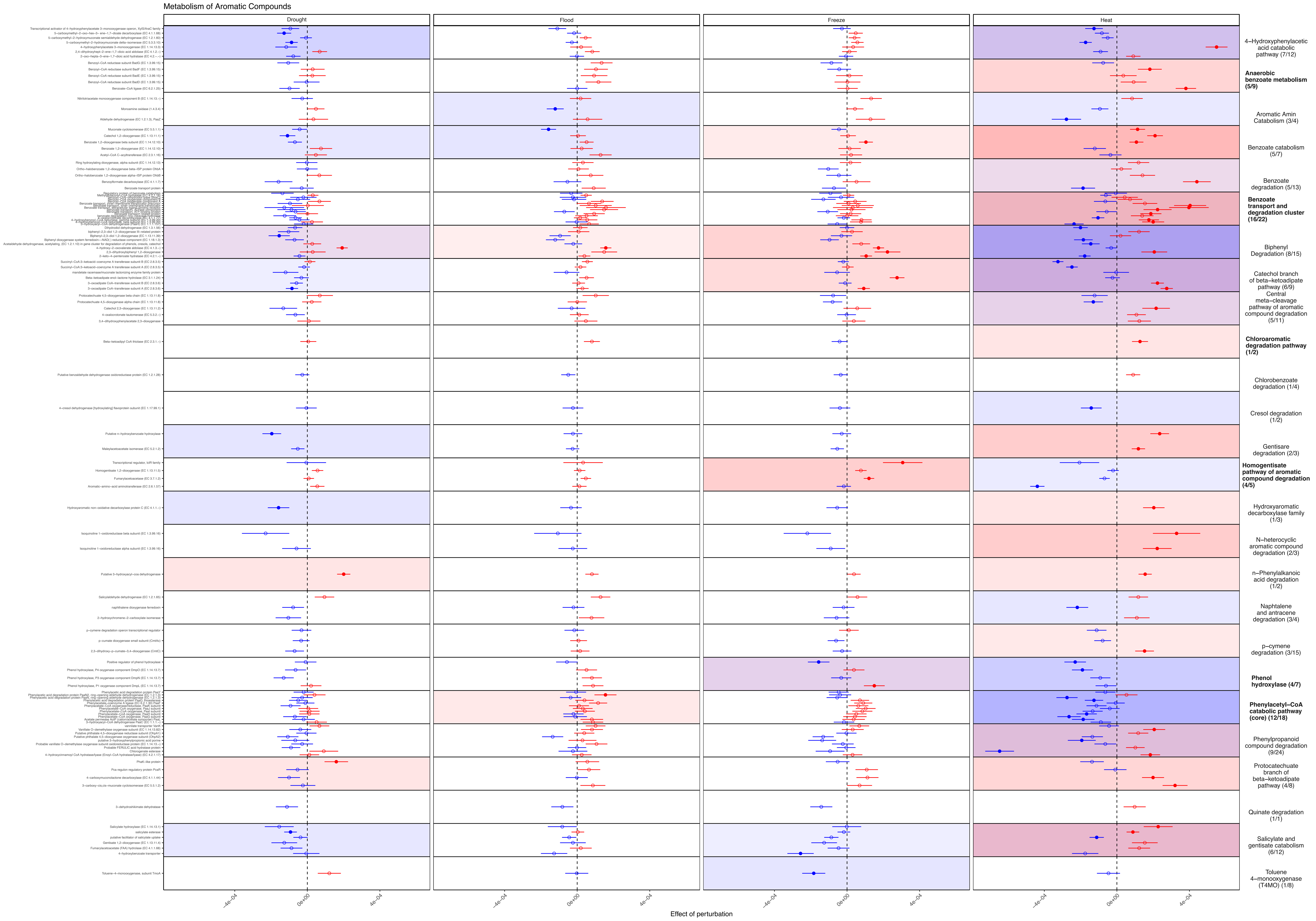
Metabolism of Aromatic Compounds
Drought
Flood
Freeze
Heat
4−Hydroxyphenylacetic acid catabolic pathway (7/12)
Anaerobic benzoate metabolism (5/9)
Aromatic Amin Catabolism (3/4)
Benzoate catabolism (5/7)
Benzoate degradation (5/13)
Benzoate transport and degradation cluster (16/22)
Biphenyl Degradation (8/15)
Catechol branch of beta−ketoadipate pathway (6/9)
Central meta−cleavage pathway of aromatic compound degradation (5/11)
Chloroaromatic degradation pathway (1/2)
Chlorobenzoate degradation (1/4)
Cresol degradation (1/2)
Gentisare degradation (2/3)
Homogentisate pathway of aromatic compound degradation (4/5)
Hydroxyaromatic decarboxylase family (1/3)
N−heterocyclic aromatic compound degradation (2/3)
n−Phenylalkanoic acid degradation (1/2)
Naphtalene and antracene degradation (3/4)
p−cymene degradation (3/15)
Phenol hydroxylase (4/7)
Phenylacetyl−CoA catabolic pathway (core) (12/18)
Phenylpropanoid compound degradation (9/24)
Protocatechuate branch of beta−ketoadipate pathway (4/8)
Quinate degradation (1/1)
Salicylate and gentisate catabolism (6/12)
Toluene 4−monooxygenase (T4MO) (1/8)
−4e−04
0e+00
4e−04
Effect of perturbation

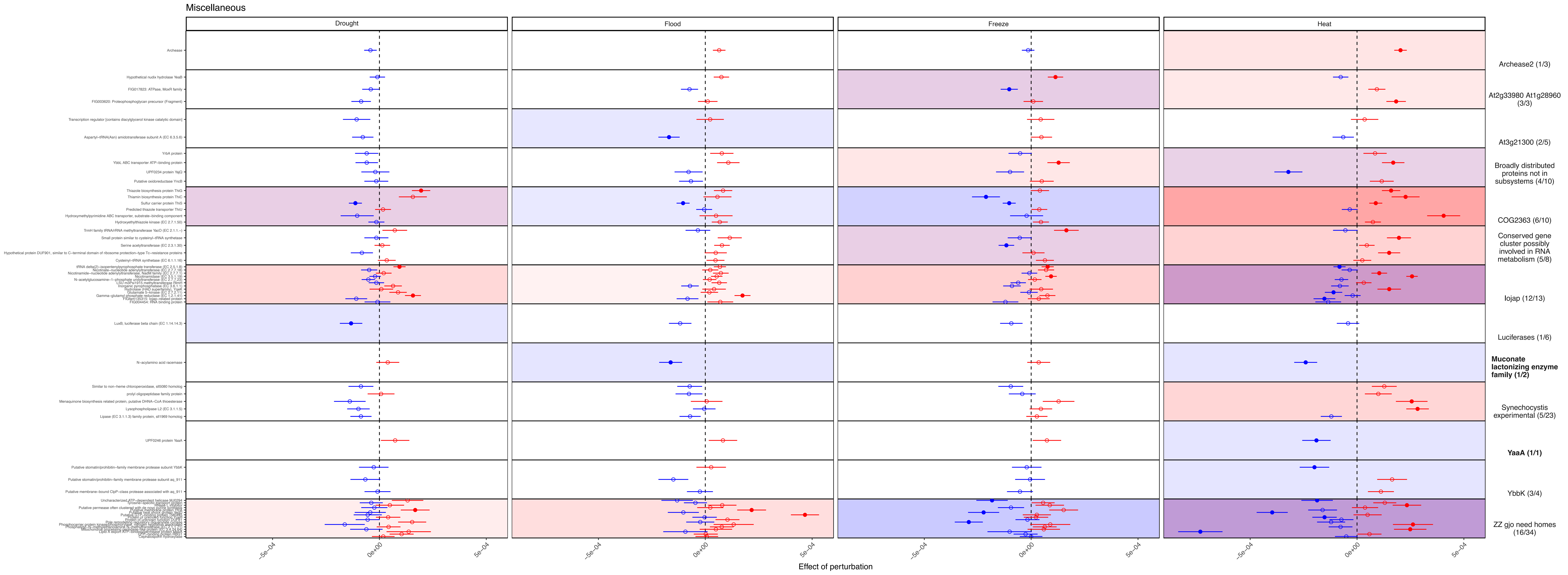
Miscellaneous
Drought
Flood
Freeze
Heat
Archease
Hypothetical nudix hydrolase YeaB
FIG017823: ATPase, MoxR family
FIG003620: Proteophosphoglycan precursor (Fragment)
Transcription regulator [contains diacylglycerol kinase catalytic domain]
Aspartyl–tRNA(Asn) amidotransferase subunit A (EC 6.3.5.6)
YrbA protein
YbbL ABC transporter ATP–binding protein
UPF0234 protein YajQ
Putative oxidoreductase YncB
Thiazole biosynthesis protein ThiG
Thiamin biosynthesis protein ThiC
Sulfur carrier protein ThiS
Predicted thiazole transporter ThiU
Hydroxymethylpyrimidine ABC transporter, substrate–binding component
Hydroxyethylthiazole kinase (EC 2.7.1.50)
TrmH family tRNA/rRNA methyltransferase YacO (EC 2.1.1.–)
Small protein similar to cysteinyl–tRNA synthetase
Serine acetyltransferase (EC 2.3.1.30)
Hypothetical protein DUF901, similar to C–terminal domain of ribosome protection–type Tc–resistance proteins
Cysteinyl–tRNA synthetase (EC 6.1.1.16)
tRNA delta(2)–isopentenylpyrophosphate transferase (EC 2.5.1.8)
Nicotinate–nucleotide adenylyltransferase (EC 2.7.7.18)
Nicotinamide–nucleotide adenylyltransferase, NadM family (EC 2.7.7.1)
Nicotinamidase (EC 3.5.1.19)
N–acetylglucosamine–1–phosphate uridyltransferase (EC 2.7.7.23)
LSU m3Psi1915 methyltransferase RlmH
Inorganic pyrophosphatase (EC 3.6.1.1)
Hydrolase (HAD superfamily), YqeK
Glutamate 5–kinase (EC 2.7.2.11)
Gamma–glutamyl phosphate reductase (EC 1.2.1.41)
FIGfam135315: Iojap–related protein
FIG004454: RNA binding protein
LuxB, luciferase beta chain (EC 1.14.14.3)
N–acylamino acid racemase
Similar to non–heme chloroperoxidase, sll5080 homolog
prolyl oligopeptidase family protein
Menaquinone biosynthesis related protein, putative DHNA–CoA thioesterase
Lysophospholipase L2 (EC 3.1.1.5)
Lipase (EC 3.1.1.3) family protein, sll1969 homolog
UPF0246 protein YaaA
Putative stomatin/prohibitin–family membrane protease subunit YbbK
Putative stomatin/prohibitin–family membrane protease subunit aq_911
Putative membrane–bound ClpP–class protease associated with aq_911
Uncharacterized ATP–dependent helicase MJ0294
Tyrosine–specific transport protein
RNase L inhibitor
Putative permease often clustered with de novo purine synthesis
Putative membrane protein YfcA
Putative heat shock protein YegD
Putative GTP–binding protien TM0445
Protein of unknown function YceH
Protein of unknown function DUF81
Pole remodelling regulatory diguanylate cyclase
Phosphocarrier protein kinase/phosphorylase, nitrogen regulation associated
Phosphatidyl–N–methylethanolamine N–methyltransferase (EC 2.1.1.71)
Mitochondrial processing peptidase–like protein (EC 3.4.24.64)
Lipid A export ATP–binding/permease protein MsbA
GTP–binding protein RBG1
Cephalosporin hydroxylase
Archease2 (1/3)
At2g33980 At1g28960 (3/3)
At3g21300 (2/5)
Broadly distributed proteins not in subsystems (4/10)
COG2363 (6/10)
Conserved gene cluster possibly involved in RNA metabolism (5/8)
Iojap (12/13)
Luciferases (1/6)
Muconate lactonizing enzyme family (1/2)
Synechocystis experimental (5/23)
YaaA (1/1)
YbbK (3/4)
ZZ gjo need homes (16/34)
−5e−04
0e+00
5e−04
Effect of perturbation

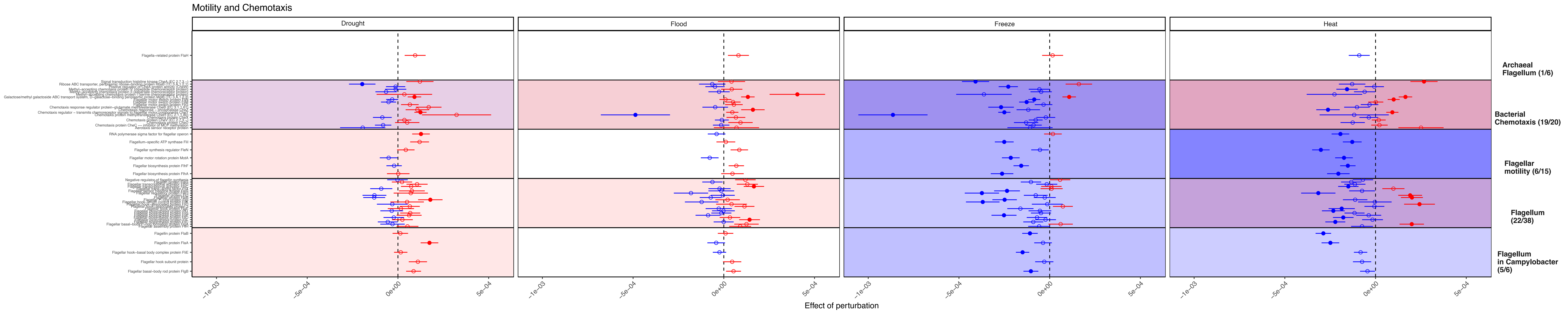

Motility and Chemotaxis
Drought
Flood
Freeze
Heat
Flagella–related protein FlaH
RNA polymerase sigma factor for flagellar operon
Flagellum–specific ATP synthase FliI
Flagellar synthesis regulator FleN
Flagellar motor rotation protein MotA
Flagellar biosynthesis protein FlhF
Flagellar biosynthesis protein FlhA
Flagellin protein FlaB
Flagellin protein FlaA
Flagellar hook–basal body complex protein FliE
Flagellar hook subunit protein
Flagellar basal–body rod protein FlgB
Archaeal Flagellum (1/6)
Bacterial Chemotaxis (19/20)
Flagellar motility (6/15)
Flagellum (22/38)
Flagellum in Campylobacter (5/6)
−1e−03
−5e−04
0e+00
5e−04
Effect of perturbation

Nitrogen Metabolism

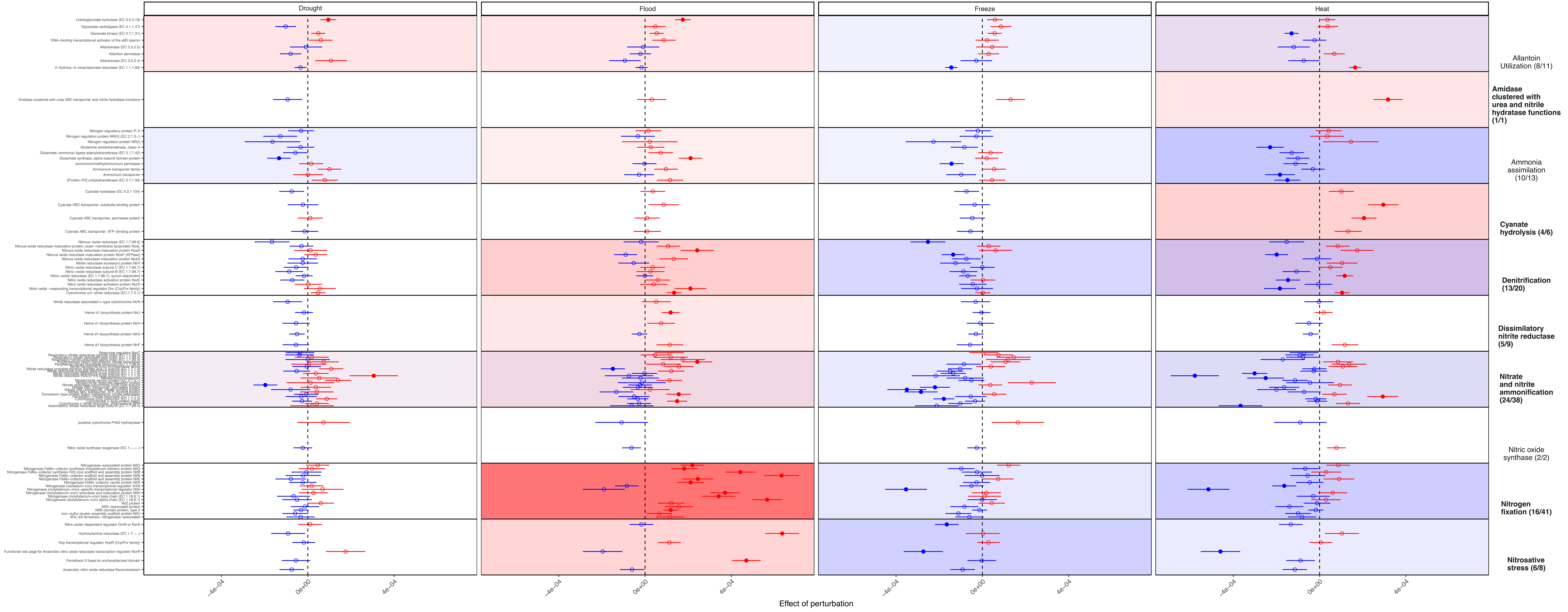

Nucleosides and Nucleotides

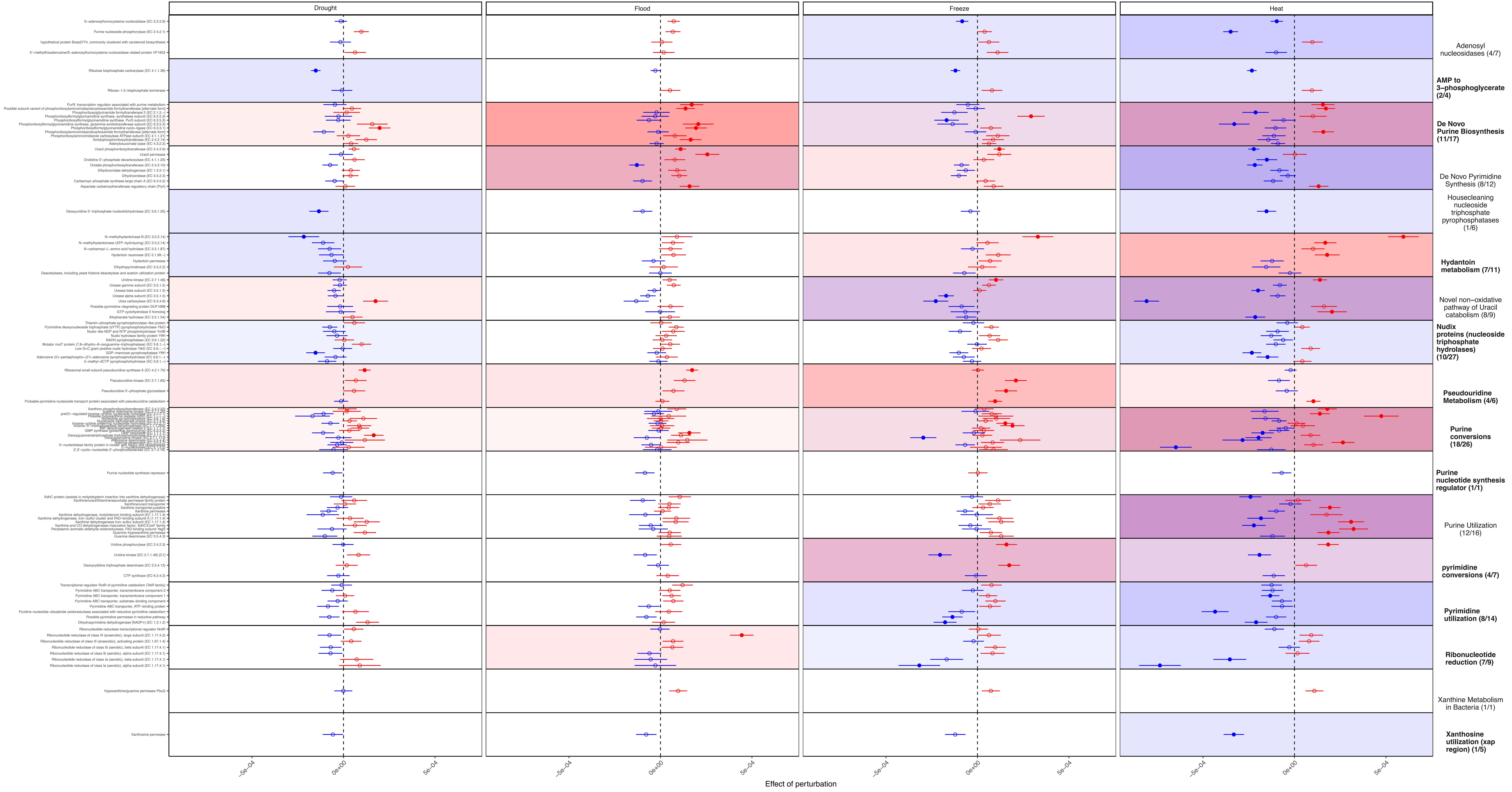

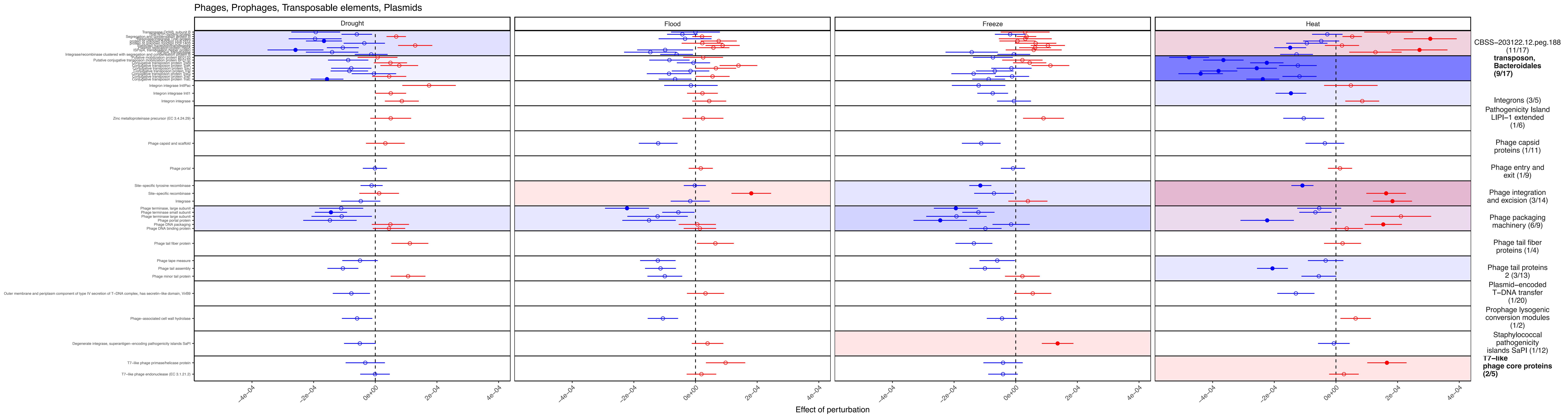

Phages, Prophages, Transposable elements, Plasmids
Drought
Flood
Freeze
Heat
Transposase OrfAB, subunit B
ThiB NTP−binding protein
Segregation and condensation protein B
Resolvase/Integrase TinR protein
protein of unknown function DUF1612
protein of unknown function DUF1403
Predicted nucleotidyltransferases
Plasmid replication protein RepA
ISPsy4, transposition helper protein
ISPsy4, transposase
Integrase/recombinase clustered with segregation and condensation protein B
Putative mobilization protein BF0133
Putative conjugative transposon mobilization protein BF0132
Conjugative transposon protein TraN
Conjugative transposon protein TraK
Conjugative transposon protein TraJ
Conjugative transposon protein TraI
Conjugative transposon protein TraG
Conjugative transposon protein TraF
Conjugative transposon protein TraE
Integron integrase IntIPac
Integron integrase IntI1
Integron integrase
Zinc metalloproteinase precursor (EC 3.4.24.29)
Phage capsid and scaffold
Phage portal
Site−specific tyrosine recombinase
Site−specific recombinase
Integrase
Phage terminase, large subunit
Phage terminase small subunit
Phage terminase large subunit
Phage portal protein
Phage DNA packaging
Phage DNA binding protein
Phage tail fiber protein
Phage tape measure
Phage tail assembly
Phage minor tail protein
Outer membrane and periplasm component of type IV secretion of T−DNA complex, has secretin−like domain, VirB9
Phage−associated cell wall hydrolase
Degenerate integrase, superantigen−encoding pathogenicity islands SaPI
T7−like phage primase/helicase protein
T7−like phage endonuclease (EC 3.1.21.2)
−4e−04
−2e−04
0e+00
2e−04
4e−04
Effect of perturbation
CBSS−203122.12.peg.188 (11/17)
transposon, Bacteroidales (9/17)
Integrons (3/5)
Pathogenicity Island LIPI−1 extended (1/6)
Phage capsid proteins (1/11)
Phage entry and exit (1/9)
Phage integration and excision (3/14)
Phage packaging machinery (6/9)
Phage tail fiber proteins (1/4)
Phage tail proteins 2 (3/13)
Plasmid−encoded T−DNA transfer (1/20)
Prophage lysogenic conversion modules (1/2)
Staphylococcal pathogenicity islands SaPI (1/12)
T7−like phage core proteins (2/5)

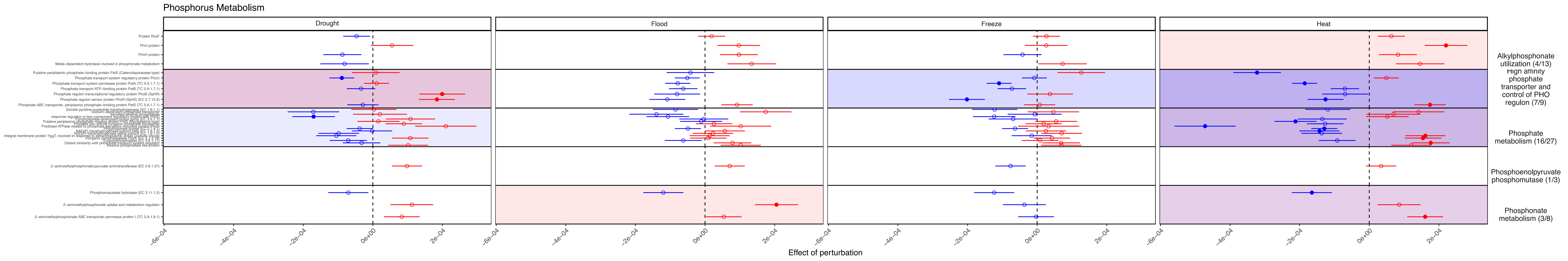
Phosphorus Metabolism
Drought
Flood
Freeze
Heat
Protein RcsF
PhnI protein
PhnH protein
Metal–dependent hydrolase involved in phosphonate metabolism
Putative periplasmic phosphate–binding protein PstS (Catenulisporaceae type)
Phosphate transport system regulatory protein PhoU
Phosphate transport system permease protein PstA (TC 3.A.1.7.1)
Phosphate transport ATP–binding protein PstB (TC 3.A.1.7.1)
Phosphate regulon transcriptional regulatory protein PhoB (SphR)
Phosphate regulon sensor protein PhoR (SphS) (EC 2.7.13.3)
Phosphate ABC transporter, periplasmic phosphate–binding protein PstS (TC 3.A.1.7.1)
Soluble pyridine nucleotide transhydrogenase (EC 1.6.1.1)
Sodium–dependent phosphate transporter
secreted alkaline phosphatase
response regulator in two–component regulatory system with PhoQ
Pyrophosphate–energized proton pump (EC 3.6.1.1)
Putative periplasmic phosphate–binding protein PstS (Mycoplasma type)
Probable low–affinity inorganic phosphate transporter
Predicted ATPase related to phosphate starvation–inducible protein PhoH
Polyphosphate kinase (EC 2.7.4.1)
NAD(P) transhydrogenase subunit beta (EC 1.6.1.2)
NAD(P) transhydrogenase alpha subunit (EC 1.6.1.2)
Integral membrane protein YggT, involved in response to extracytoplasmic stress (osmotic shock)
Inorganic pyrophospatase PpaX (EC 3.1.3.18)
Exopolyphosphatase (EC 3.6.1.11)
Distant similarity with phosphate transport system regulator
Alkaline phosphatase like protein
2–aminoethylphosphonate:pyruvate aminotransferase (EC 2.6.1.37)
Phosphonoacetate hydrolase (EC 3.11.1.2)
2–aminoethylphosphonate uptake and metabolism regulator
2–aminoethylphosphonate ABC transporter permease protein I (TC 3.A.1.9.1)
Alkylphosphonate utilization (4/13)
High affinity phosphate transporter and control of PHO regulon (7/9)
Phosphate metabolism (16/27)
Phosphoenolpyruvate phosphomutase (1/3)
Phosphonate metabolism (3/8)
–6e–04
–4e–04
–2e–04
0e+00
2e–04
Effect of perturbation

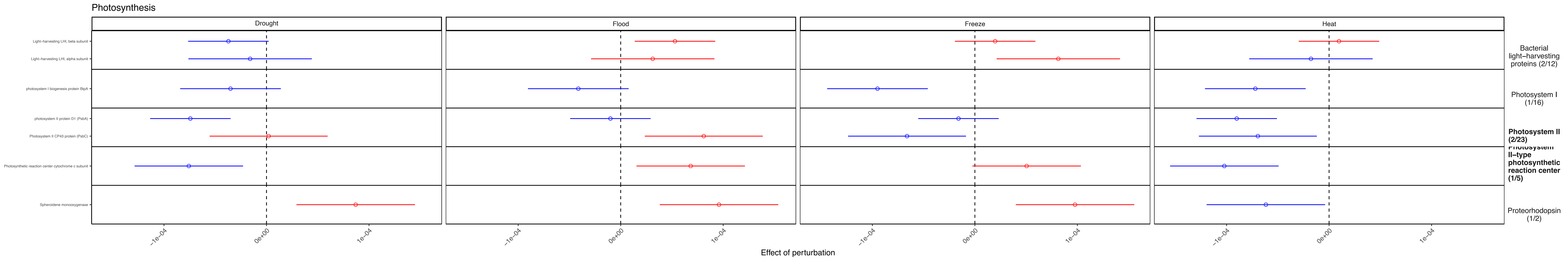
Photosynthesis
Drought
Flood
Freeze
Heat
Light–harvesting LHI, beta subunit
Light–harvesting LHI, alpha subunit
photosystem I biogenesis protein BtpA
photosystem II protein D1 (PsbA)
Photosystem II CP43 protein (PsbC)
Photosynthetic reaction center cytochrome c subunit
Spheroidene monooxygenase
Bacterial light–harvesting proteins (2/12)
Photosystem I (1/16)
Photosystem II (2/23)
Photosystem II–type photosynthetic reaction center (1/5)
Proteorhodopsin (1/2)
−1e−04
0e+00
1e−04
Effect of perturbation

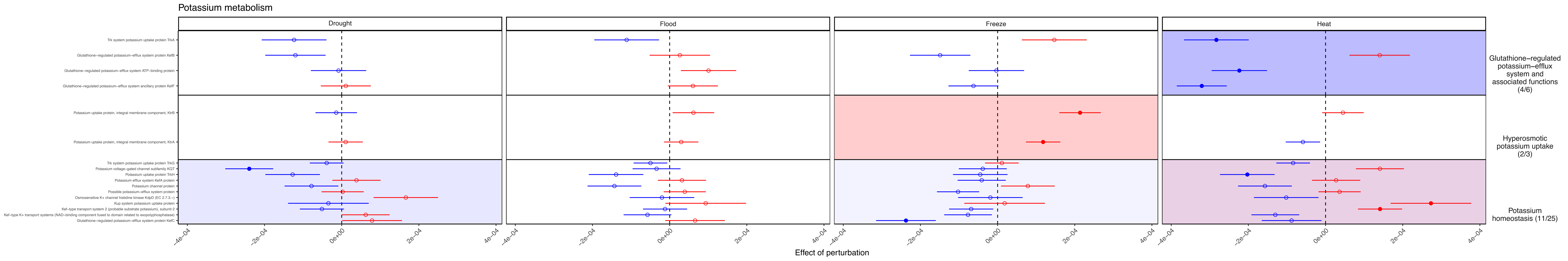

Potassium metabolism
Drought
Flood
Freeze
Heat
Trk system potassium uptake protein TrkA
Glutathione–regulated potassium–efflux system protein KefB
Glutathione–regulated potassium–efflux system ATP–binding protein
Glutathione–regulated potassium–efflux system ancillary protein KefF
Potassium uptake protein, integral membrane component, KtrB
Potassium uptake protein, integral membrane component, KtrA
Trk system potassium uptake protein TrkG
Potassium voltage–gated channel subfamily KQT
Potassium uptake protein TrkH
Potassium efflux system KefA protein
Potassium channel protein
Possible potassium–efflux system protein
Osmosensitive K+ channel histidine kinase KdpD (EC 2.7.3.–)
Kup system potassium uptake protein
Kef–type transport system 2 (probable substrate potassium), subunit 2
Kef–type K+ transport systems (NAD–binding component fused to domain related to exopolyphosphatase)
Glutathione–regulated potassium–efflux system protein KefC
Glutathione–regulated potassium–efflux system and associated functions (4/6)
Hyperosmotic potassium uptake (2/3)
Potassium homeostasis (11/25)
−4e−04
−2e−04
0e+00
2e−04
4e−04
Effect of perturbation

Protein Metabolism

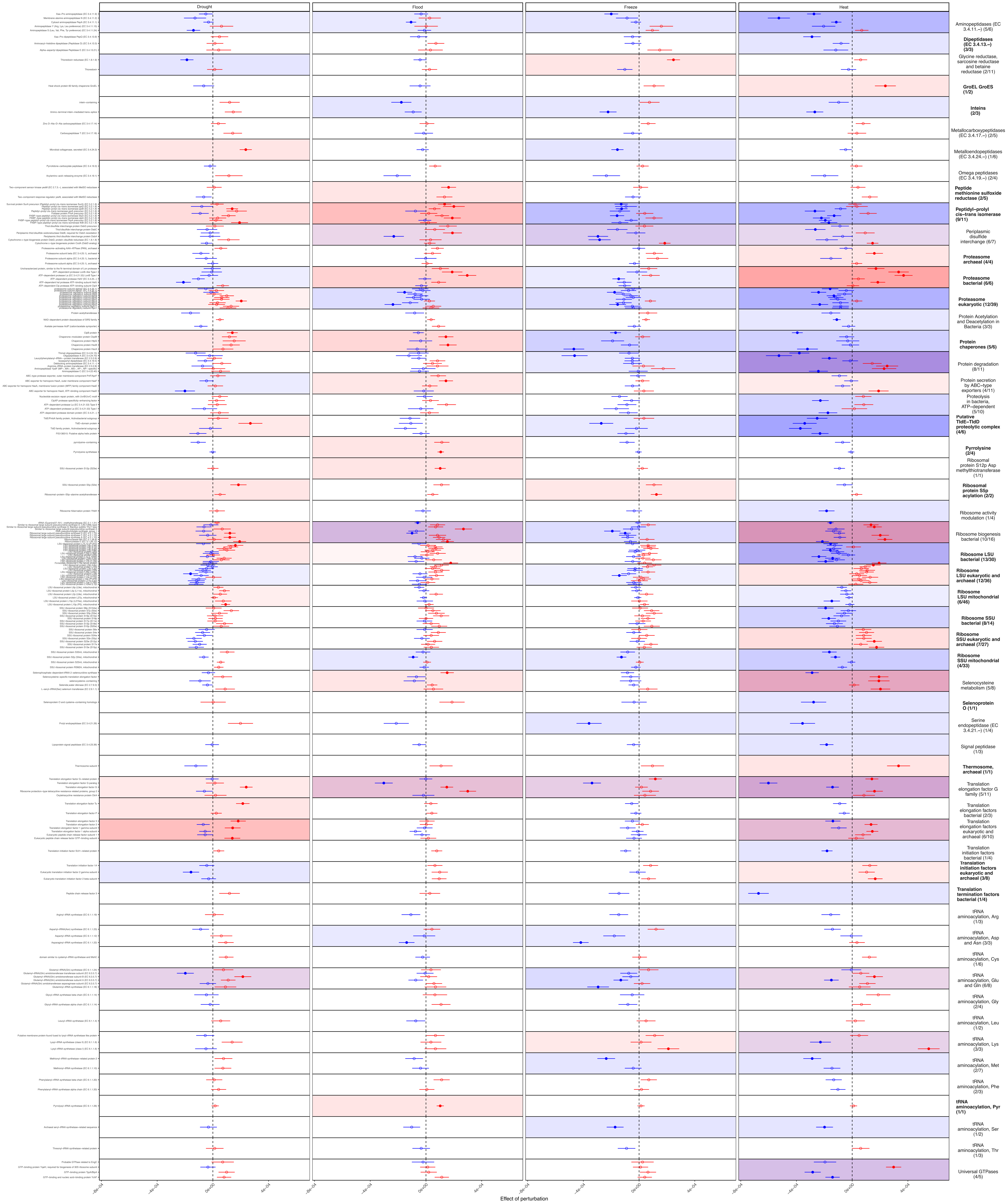

Regulation and Cell signaling

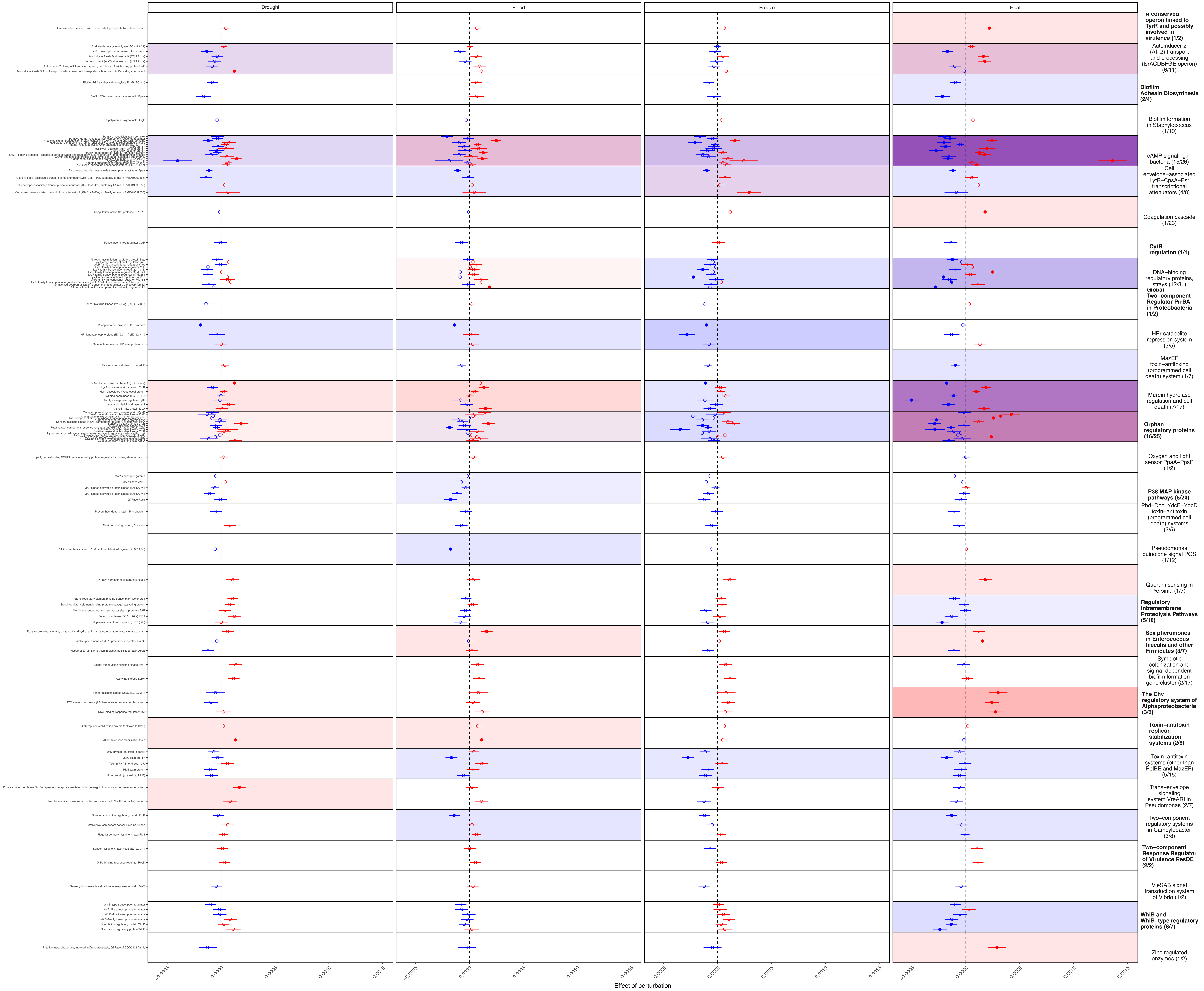

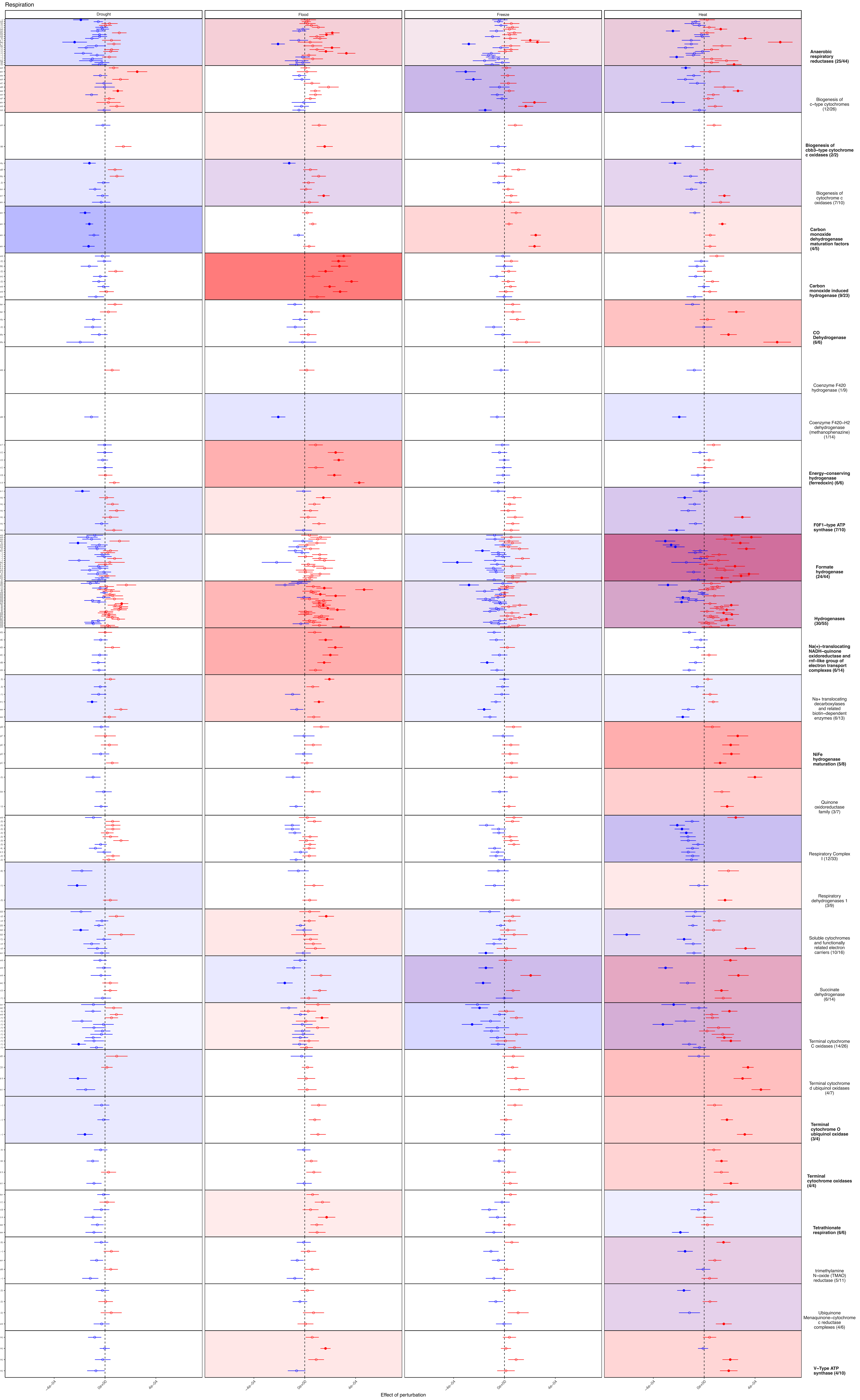

Respiration
Drought
Flood
Freeze
Heat
Anaerobic respiratory reductases (25/44)
Biogenesis of c−type cytochromes (12/26)
Biogenesis of cbb3−type cytochrome c oxidases (2/2)
Biogenesis of cytochrome c oxidases (7/10)
Carbon monoxide dehydrogenase maturation factors (4/5)
Carbon monoxide induced hydrogenase (9/23)
CO Dehydrogenase (6/6)
Coenzyme F420 hydrogenase (1/9)
Coenzyme F420−H2 dehydrogenase (methanophenazine) (1/14)
Energy−conserving hydrogenase (ferredoxin) (6/6)
F0F1−type ATP synthase (7/10)
Formate hydrogenase (24/44)
Hydrogenases (30/55)
Na(+)−translocating NADH−quinone oxidoreductase and rnf−like group of electron transport complexes (6/14)
Na+ translocating decarboxylases and related biotin−dependent enzymes (6/13)
NiFe hydrogenase maturation (5/8)
Quinone oxidoreductase family (3/7)
Respiratory Complex I (12/33)
Respiratory dehydrogenases 1 (3/9)
Soluble cytochromes and functionally related electron carriers (10/16)
Succinate dehydrogenase (6/14)
Terminal cytochrome C oxidases (14/26)
Terminal cytochrome d ubiquinol oxidases (4/7)
Terminal cytochrome O ubiquinol oxidase (3/4)
Terminal cytochrome oxidases (4/4)
Tetrathionate respiration (6/6)
trimethylamine N−oxide (TMAO) reductase (5/11)
Ubiquinone Menaquinone−cytochrome c reductase complexes (4/6)
V−Type ATP synthase (4/10)
−4e−04
0e+00
4e−04
Effect of perturbation

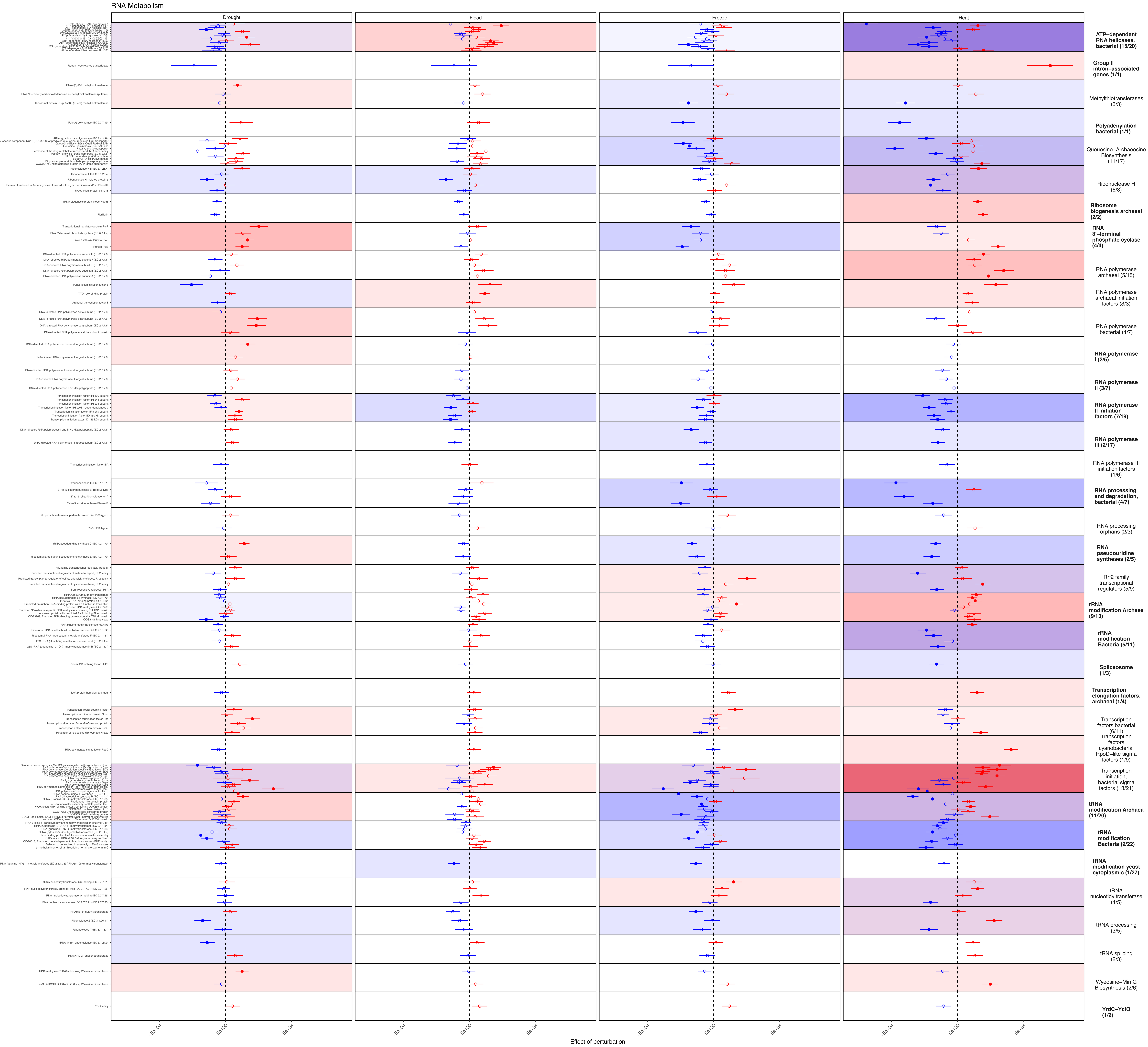
RNA Metabolism
Drought
Flood
Freeze
Heat
ATP–dependent RNA helicases, bacterial (15/20)
Group II intron–associated genes (1/1)
Methylthiotransferases (3/3)
Polyadenylation bacterial (1/1)
Queuosine–Archaeosine Biosynthesis (11/17)
Ribonuclease H (5/8)
Ribosome biogenesis archaeal (2/2)
RNA 3'–terminal phosphate cyclase (4/4)
RNA polymerase archaeal (5/15)
RNA polymerase archaeal initiation factors (3/3)
RNA polymerase bacterial (4/7)
RNA polymerase I (2/5)
RNA polymerase II (3/7)
RNA polymerase II initiation factors (7/19)
RNA polymerase III (2/17)
RNA polymerase III initiation factors (1/6)
RNA processing and degradation, bacterial (4/7)
RNA processing orphans (2/3)
RNA pseudouridine syntheses (2/5)
Rrf2 family transcriptional regulators (5/9)
rRNA modification Archaea (9/13)
rRNA modification Bacteria (5/11)
Spliceosome (1/3)
Transcription elongation factors, archaeal (1/4)
Transcription factors bacterial (6/11)
Transcription factors cyanobacterial RpoD–like sigma factors (1/9)
Transcription initiation, bacterial sigma factors (13/21)
tRNA modification Archaea (11/20)
tRNA modification Bacteria (9/22)
tRNA modification yeast cytoplasmic (1/27)
tRNA nucleotidyltransferase (4/5)
tRNA processing (3/5)
tRNA splicing (2/3)
Wyeosine–MimG Biosynthesis (2/6)
YrdC–YciO (1/2)
−5e−04
0e+00
5e−04
Effect of perturbation

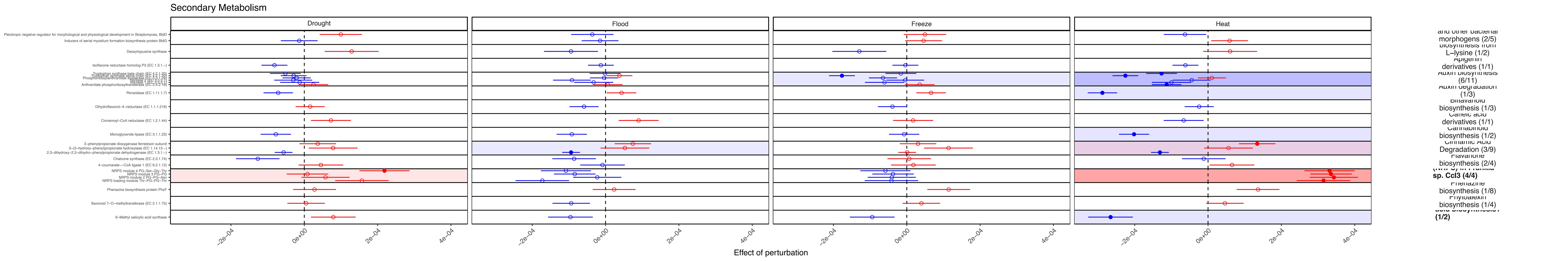
Secondary Metabolism
Drought
Flood
Freeze
Heat
Pleiotropic negative regulator for morphological and physiological development in Streptomyces, BldD
Inducers of aerial mycelium formation biosynthesis protein BldG
Deoxyhypusine synthase
Isoflavone reductase homolog P3 (EC 1.3.1.–)
Tryptophan synthase beta chain (EC 4.2.1.20)
Tryptophan synthase alpha chain (EC 4.2.1.20)
Phosphoribosylanthranilate isomerase (EC 5.3.1.24)
Nitrilase 2 (EC 3.5.5.1)
Nitrilase 1 (EC 3.5.5.1)
Anthranilate phosphoribosyltransferase (EC 2.4.2.18)
Peroxidase (EC 1.11.1.7)
Dihydroflavonol–4–reductase (EC 1.1.1.219)
Cinnamoyl–CoA reductase (EC 1.2.1.44)
Monoglyceride lipase (EC 3.1.1.23)
3–phenylpropionate dioxygenase ferredoxin subunit
3–(3–hydroxy–phenyl)propionate hydroxylase (EC 1.14.13.–)
2,3–dihydroxy–2,3–dihydro–phenylpropionate dehydrogenase (EC 1.3.1.–)
Chalcone synthase (EC 2.3.1.74)
4–coumarate––CoA ligase 1 (EC 6.2.1.12)
NRPS module 4 PG–Ser–Gly–Thr
NRPS module 3 PG–PG
NRPS module 2 PG–PG–Asn
NRPS loading module Thr–PG–PG–Thr
Phenazine biosynthesis protein PhzF
flavonoid 7–O–methyltransferase (EC 2.1.1.75)
6–Methyl salicylic acid synthase
morphogens (2/5)
L–lysine (1/2)
derivatives (1/1)
(6/11)
(1/3)
biosynthesis (1/3)
derivatives (1/1)
biosynthesis (1/2)
Degradation (3/9)
biosynthesis (2/4)
sp. Ccl3 (4/4)
biosynthesis (1/8)
biosynthesis (1/4)
(1/2)
−2e−04
0e+00
2e−04
4e−04
Effect of perturbation

Stress Response

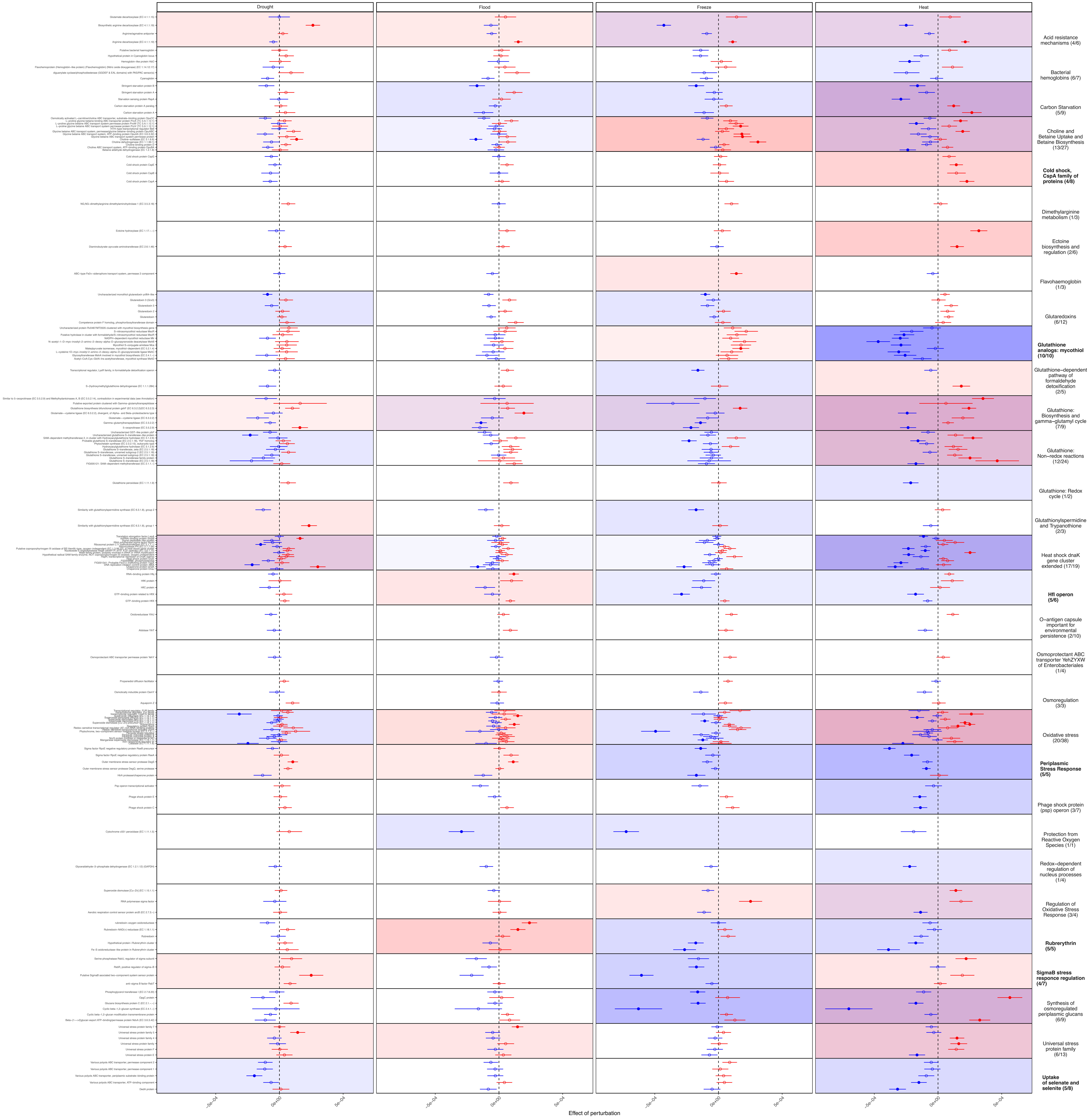

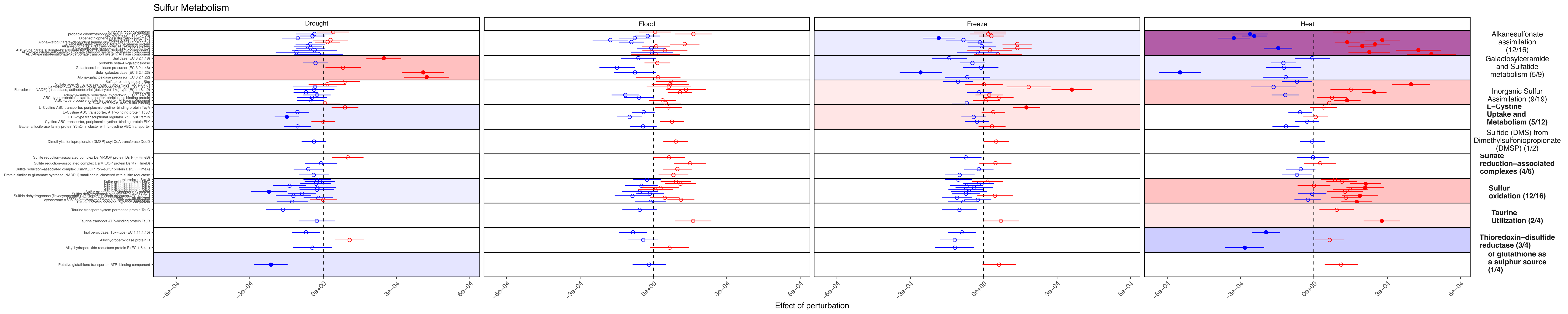

Sulfur Metabolism
Drought
Flood
Freeze
Heat
Alkanesulfonate assimilation (12/16)
Galactosylceramide and Sulfatide metabolism (5/9)
Inorganic Sulfur Assimilation (9/19)
L-Cystine Uptake and Metabolism (5/12)
Sulfide (DMS) from Dimethylsulfoniopropionate (DMSP) (1/2)
Sulfate reduction-associated complexes (4/6)
Sulfur oxidation (12/16)
Taurine Utilization (2/4)
Thioredoxin-disulfide reductase (3/4)
of glutathione as a sulphur source (1/4)
Effect of perturbation

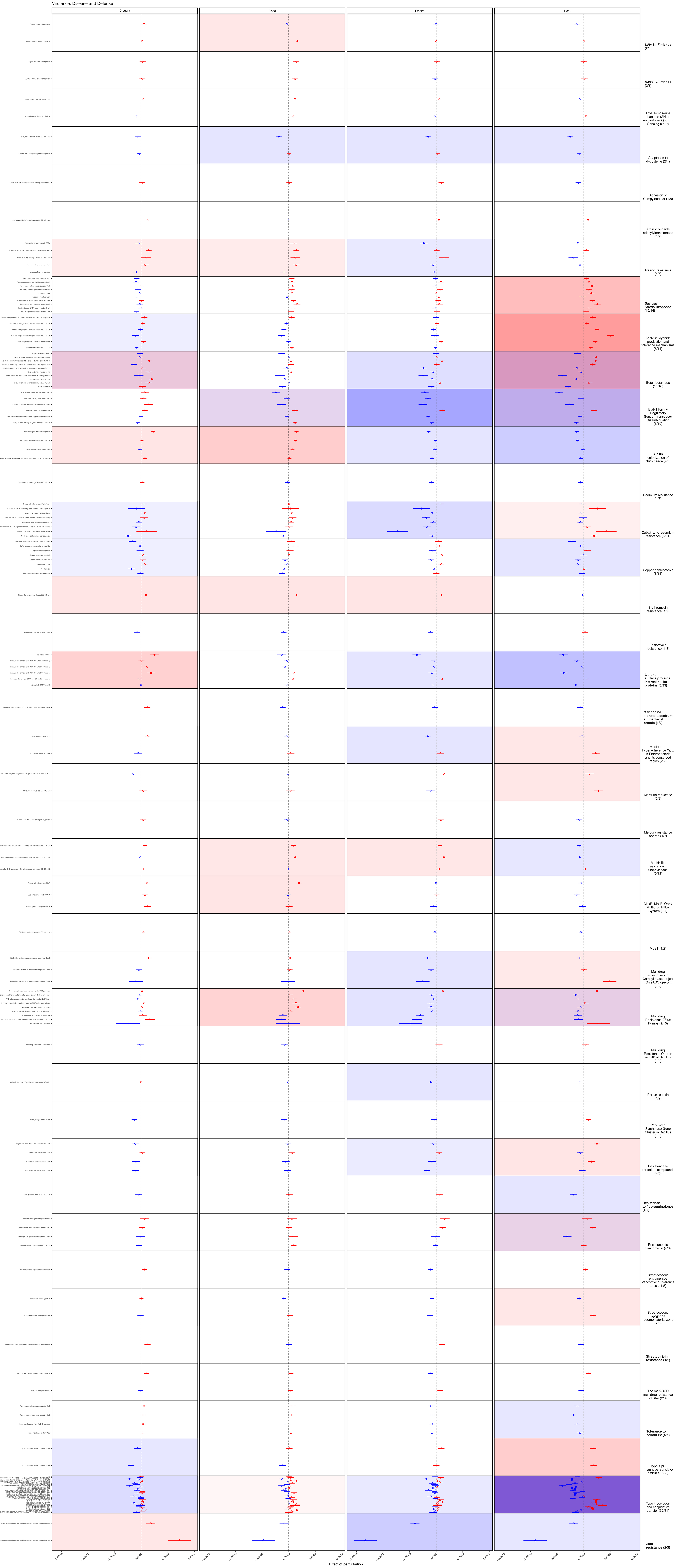

Virulence, Disease and Defense
Drought
Flood
Freeze
Heat
β–Fimbriae (2/3)
σ–Fimbriae (2/5)
Acyl Homoserine Lactone (AHL) Autoinducer Quorum Sensing (2/10)
Adaptation to d–cysteine (2/4)
Adhesion of Campylobacter (1/8)
Aminoglycoside adenylyltransferases (1/2)
Arsenic resistance (5/6)
Bacitracin Stress Response (10/14)
Bacterial cyanide production and tolerance mechanisms (6/14)
Beta–lactamase (10/16)
BlaR1 Family Regulatory Sensor–transducer Disambiguation (6/10)
C jejuni colonization of chick caeca (4/8)
Cadmium resistance (1/3)
Cobalt–zinc–cadmium resistance (8/21)
Copper homeostasis (8/14)
Erythromycin resistance (1/2)
Fosfomycin resistance (1/3)
Listeria surface proteins: Internalin–like proteins (6/33)
Marinocine, a broad–spectrum antibacterial protein (1/2)
Mediator of hyperadherence YidE in Enterobacteria and its conserved region (2/7)
Mercuric reductase (2/2)
Mercury resistance operon (1/7)
Methicillin resistance in Staphylococci (3/12)
MexE–MexF–OprN Multidrug Efflux System (3/4)
MLST (1/2)
Multidrug efflux pump in Campylobacter jejuni (CmeABC operon) (3/4)
Multidrug Resistance Efflux Pumps (9/15)
Multidrug Resistance Operon mdtRP of Bacillus (1/2)
Pertussis toxin (1/2)
Polymyxin Synthetase Gene Cluster in Bacillus (1/4)
Resistance to chromium compounds (4/5)
Resistance to fluoroquinolones (1/2)
Resistance to Vancomycin (4/8)
Streptococcus pneumoniae Vancomycin Tolerance Locus (1/5)
Streptococcus pyogenes recombinatorial zone (2/6)
Streptothricin resistance (1/1)
The mdtABCD multidrug resistance cluster (2/6)
Tolerance to colicin E2 (4/5)
Type 1 pili (mannose–sensitive fimbriae) (2/8)
Type 4 secretion and conjugative transfer (32/61)
Zinc resistance (2/3)
-0.0015
-0.0010
-0.0005
0.0000
0.0005
0.0010
Effect of perturbation
