# Supplementary material for: Soil microbiomes show consistent and predictable responses to extreme events
Source: Nature. 2024 Nov 27;636(8043):690–6. doi: 10.1038/s41586-024-08185-3 (PMC11655354; doi:10.1038/s41586-024-08185-3)
Supplement: Supplementary file 5 — This multipage PDF file contains the functional resilience of our simulated extreme events at all functional levels. These plots bear the same relationship to Extended Data Fig. 4 as Supplementary Fig. 5 to Fig. 3a—that is, they report the resilience effects for models fitted at the individual protein level, with bolding for higher-level function where that changes significantly over time. Colours are as in Extended Data Fig. 4, with red indicating resilience and blue indicating change over time in the same direction as the initial impact. Thus, a positive value coloured red indicates a function that was initially reduced and subsequently increased, whereas a positive value coloured blue indicates an increase over time following an initial positive impact of perturbation relative to control. As in Supplementary Fig. 5, background colours are contributed to by all significantly changed proteins within the higher-level function indicated on the right. [file 41586_2024_8185_MOESM5_ESM.pdf]

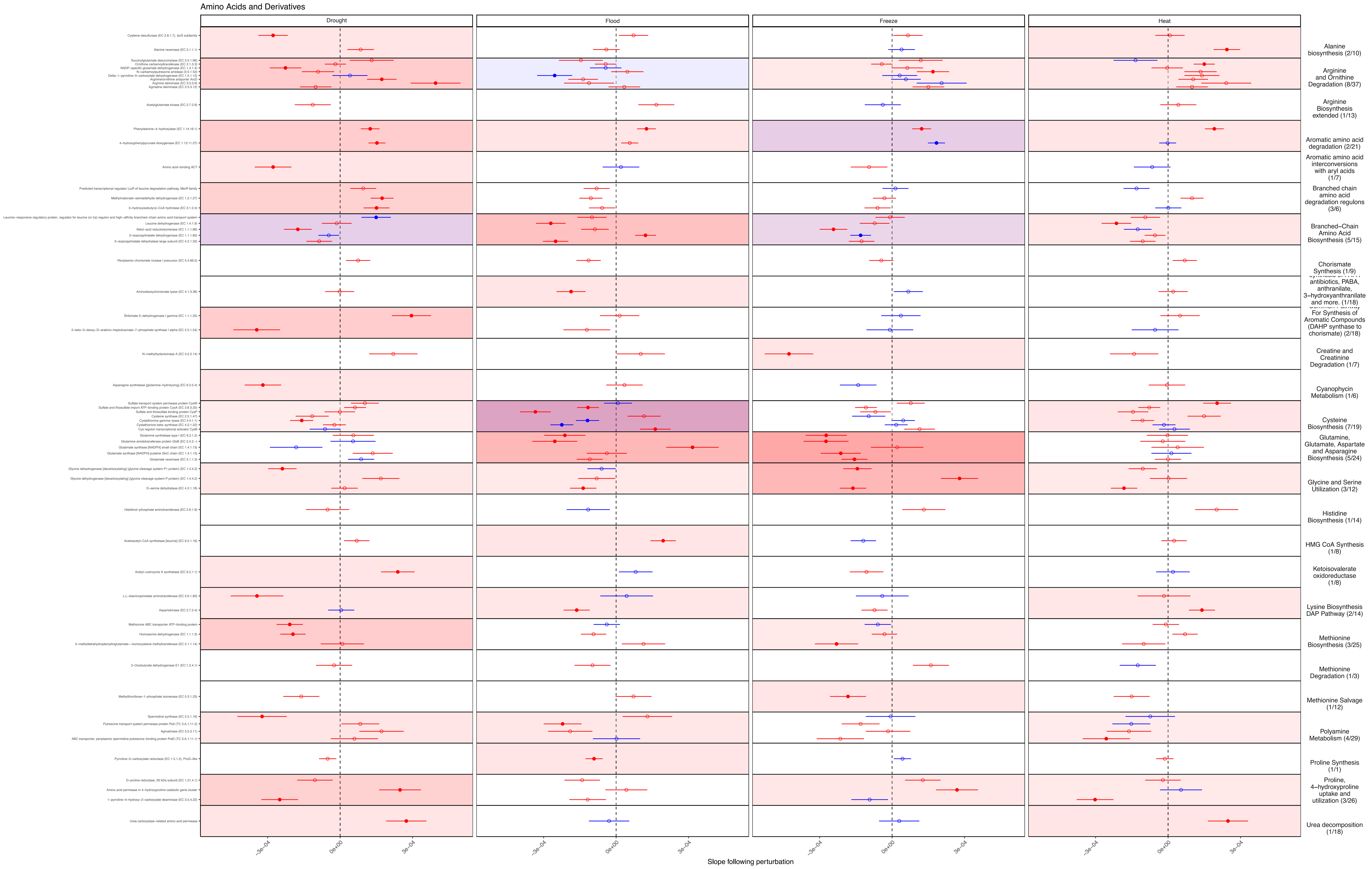

Amino Acids and Derivatives
Drought
Flood
Freeze
Heat
Cysteine desulfurase (EC 2.8.1.7), IscS subfamily
Alanine racemase (EC 5.1.1.1)
Succinylglutamate desuccinylase (EC 3.5.1.96)
Ornithine carbamoyltransferase (EC 2.1.3.3)
NADP−specific glutamate dehydrogenase (EC 1.4.1.4)
N−carbamoylputrescine amidase (3.5.1.53)
Delta−1−pyrroline−5−carboxylate dehydrogenase (EC 1.5.1.12)
Arginine/ornithine antiporter ArcD
Arginine deiminase (EC 3.5.3.6)
Agmatine deiminase (EC 3.5.3.12)
Acetylglutamate kinase (EC 2.7.2.8)
Phenylalanine−4−hydroxylase (EC 1.14.16.1)
4−hydroxyphenylpyruvate dioxygenase (EC 1.13.11.27)
Amino acid−binding ACT
Predicted transcriptional regulator LiuR of leucine degradation pathway, MerR family
Methylmalonate−semialdehyde dehydrogenase (EC 1.2.1.27)
3−hydroxyisobutyryl−CoA hydrolase (EC 3.1.2.4)
Leucine−responsive regulatory protein, regulator for leucine (or lrp) regulon and high−affinity branched−chain amino acid transport system
Leucine dehydrogenase (EC 1.4.1.9)
Ketol−acid reductoisomerase (EC 1.1.1.86)
3−isopropylmalate dehydrogenase (EC 1.1.1.85)
3−isopropylmalate dehydratase large subunit (EC 4.2.1.33)
Periplasmic chorismate mutase I precursor (EC 5.4.99.5)
Aminodeoxychorismate lyase (EC 4.1.3.38)
Shikimate 5−dehydrogenase I gamma (EC 1.1.1.25)
2−keto−3−deoxy−D−arabino−heptulosonate−7−phosphate synthase I alpha (EC 2.5.1.54)
N−methylhydantoinase A (EC 3.5.2.14)
Asparagine synthetase [glutamine−hydrolyzing] (EC 6.3.5.4)
Sulfate transport system permease protein CysW
Sulfate and thiosulfate import ATP−binding protein CysA (EC 3.6.3.25)
Sulfate and thiosulfate binding protein CysP
Cysteine synthase (EC 2.5.1.47)
Cystathionine gamma−lyase (EC 4.4.1.1)
Cystathionine beta−synthase (EC 4.2.1.22)
Cys regulon transcriptional activator CysB
Glutamine synthetase type I (EC 6.3.1.2)
Glutamine amidotransferase protein GlxB (EC 2.4.2.−)
Glutamate synthase [NADPH] small chain (EC 1.4.1.13)
Glutamate synthase [NADPH] putative GlxC chain (EC 1.4.1.13)
Glutamate racemase (EC 5.1.1.3)
Glycine dehydrogenase [decarboxylating] (glycine cleavage system P1 protein) (EC 1.4.4.2)
Glycine dehydrogenase [decarboxylating] (glycine cleavage system P protein) (EC 1.4.4.2)
D−serine dehydratase (EC 4.3.1.18)
Histidinol−phosphate aminotransferase (EC 2.6.1.9)
Acetoacetyl−CoA synthetase [leucine] (EC 6.2.1.16)
Acetyl−coenzyme A synthetase (EC 6.2.1.1)
L,L−diaminopimelate aminotransferase (EC 2.6.1.83)
Aspartokinase (EC 2.7.2.4)
Methionine ABC transporter ATP−binding protein
Homoserine dehydrogenase (EC 1.1.1.3)
5−methyltetrahydropteroyltriglutamate−−homocysteine methyltransferase (EC 2.1.1.14)
2−Oxobutyrate dehydrogenase E1 (EC 1.2.4.1)
Methylthioribose−1−phosphate isomerase (EC 5.3.1.23)
Spermidine synthase (EC 2.5.1.16)
Putrescine transport system permease protein PotI (TC 3.A.1.11.2)
Agmatinase (EC 3.5.3.11)
ABC transporter, periplasmic spermidine putrescine−binding protein PotD (TC 3.A.1.11.1)
Pyrroline−5−carboxylate reductase (EC 1.5.1.2), ProG−like
D−proline reductase, 26 kDa subunit (EC 1.21.4.1)
Amino acid permease in 4−hydroxyproline catabolic gene cluster
1−pyrroline−4−hydroxy−2−carboxylate deaminase (EC 3.5.4.22)
Urea carboxylase−related amino acid permease
Alanine biosynthesis (2/10)
Arginine and Ornithine Degradation (8/37)
Arginine Biosynthesis extended (1/13)
Aromatic amino acid degradation (2/21)
Aromatic amino acid interconversions with aryl acids (1/7)
Branched chain amino acid degradation regulons (3/6)
Branched−Chain Amino Acid Biosynthesis (5/15)
Chorismate Synthesis (1/9)
antibiotics, PABA, anthranilate, 3−hydroxyanthranilate and more. (1/18)
For Synthesis of Aromatic Compounds (DAHP synthase to chorismate) (2/18)
Creatine and Creatinine Degradation (1/7)
Cyanophycin Metabolism (1/6)
Cysteine Biosynthesis (7/19)
Glutamine, Glutamate, Aspartate and Asparagine Biosynthesis (5/24)
Glycine and Serine Utilization (3/12)
Histidine Biosynthesis (1/14)
HMG CoA Synthesis (1/8)
Ketoisovalerate oxidoreductase (1/8)
Lysine Biosynthesis DAP Pathway (2/14)
Methionine Biosynthesis (3/25)
Methionine Degradation (1/3)
Methionine Salvage (1/12)
Polyamine Metabolism (4/29)
Proline Synthesis (1/1)
Proline, 4−hydroxyproline uptake and utilization (3/26)
Urea decomposition (1/18)
−3e−04
0e+00
3e−04
Slope following perturbation

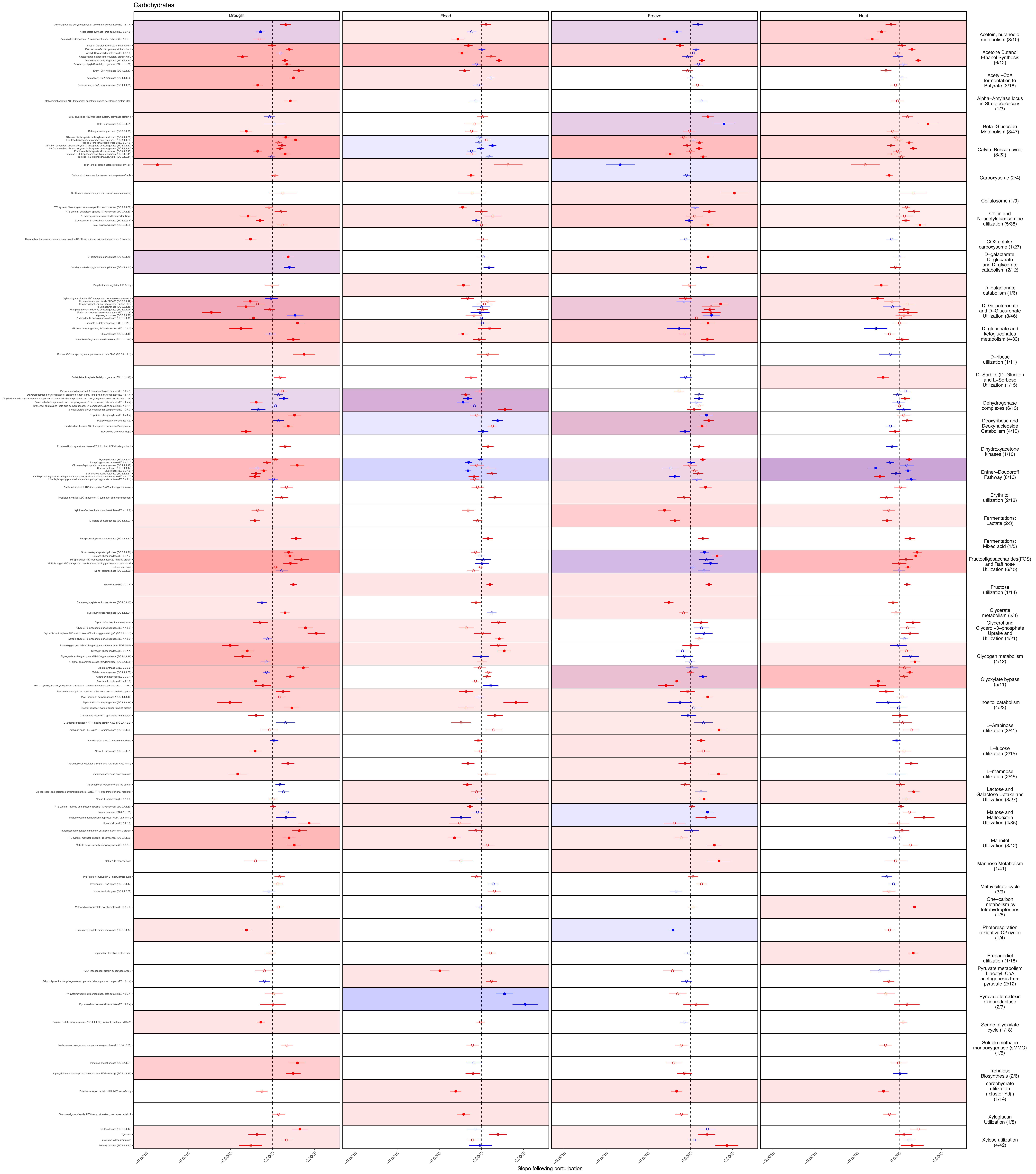

Carbohydrates
Drought
Flood
Freeze
Heat
Acetoin, butanediol metabolism (3/10)
Acetone Butanol Ethanol Synthesis (6/12)
Acetyl−CoA fermentation to Butyrate (3/16)
Alpha−Amylase locus in Streptocococcus (1/3)
Beta−Glucoside Metabolism (3/47)
Calvin−Benson cycle (8/22)
Carboxysome (2/4)
Cellulosome (1/9)
Chitin and N−acetylglucosamine utilization (5/38)
CO2 uptake, carboxysome (1/27)
D−galactarate, D−glucarate and D−glycerate catabolism (2/12)
D−galactonate catabolism (1/6)
D−Galacturonate and D−Glucuronate Utilization (8/46)
D−gluconate and ketogluconates metabolism (4/33)
D−ribose utilization (1/11)
D−Sorbitol(D−Glucitol) and L−Sorbose Utilization (1/15)
Dehydrogenase complexes (6/13)
Deoxyribose and Deoxynucleoside Catabolism (4/15)
Dihydroxyacetone kinases (1/10)
Entner−Doudoroff Pathway (8/16)
Erythritol utilization (2/13)
Fermentations: Lactate (2/3)
Fermentations: Mixed acid (1/5)
Fructooligosaccharides(FOS) and Raffinose Utilization (6/15)
Fructose utilization (1/14)
Glycerate metabolism (2/4)
Glycerol and Glycerol−3−phosphate Uptake and Utilization (4/21)
Glycogen metabolism (4/12)
Glyoxylate bypass (5/11)
Inositol catabolism (4/23)
L−Arabinose utilization (3/41)
L−fucose utilization (2/15)
L−rhamnose utilization (2/46)
Lactose and Galactose Uptake and Utilization (3/27)
Maltose and Maltodextrin Utilization (4/35)
Mannitol Utilization (3/12)
Mannose Metabolism (1/41)
Methylcitrate cycle (3/9)
One−carbon metabolism by tetrahydropterines (1/5)
Photorespiration (oxidative C2 cycle) (1/4)
Propanediol utilization (1/18)
Pyruvate metabolism II: acetyl−CoA, acetogenesis from pyruvate (2/12)
Pyruvate:ferredoxin oxidoreductase (2/7)
Serine−glyoxylate cycle (1/18)
Soluble methane monooxygenase (sMMO) (1/5)
Trehalose Biosynthesis (2/6)
carbohydrate utilization (cluster Ydj) (1/14)
Xyloglucan Utilization (1/8)
Xylose utilization (4/42)
−0.0015
−0.0010
−0.0005
0.0000
0.0005
Slope following perturbation

Cell Division and Cell Cycle

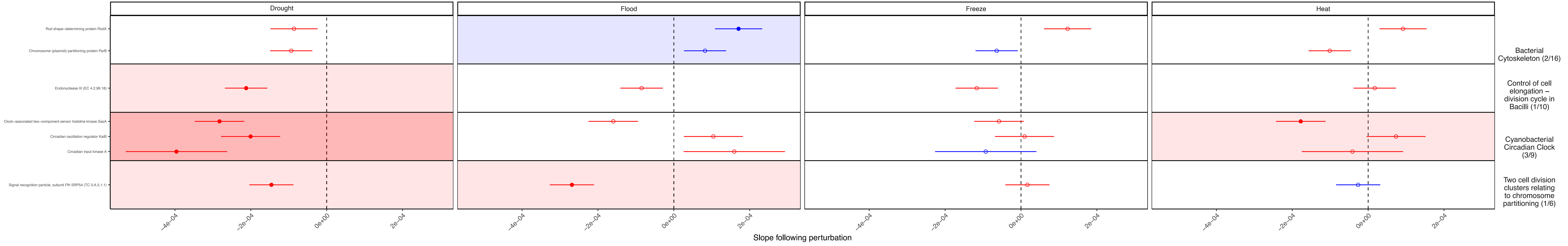

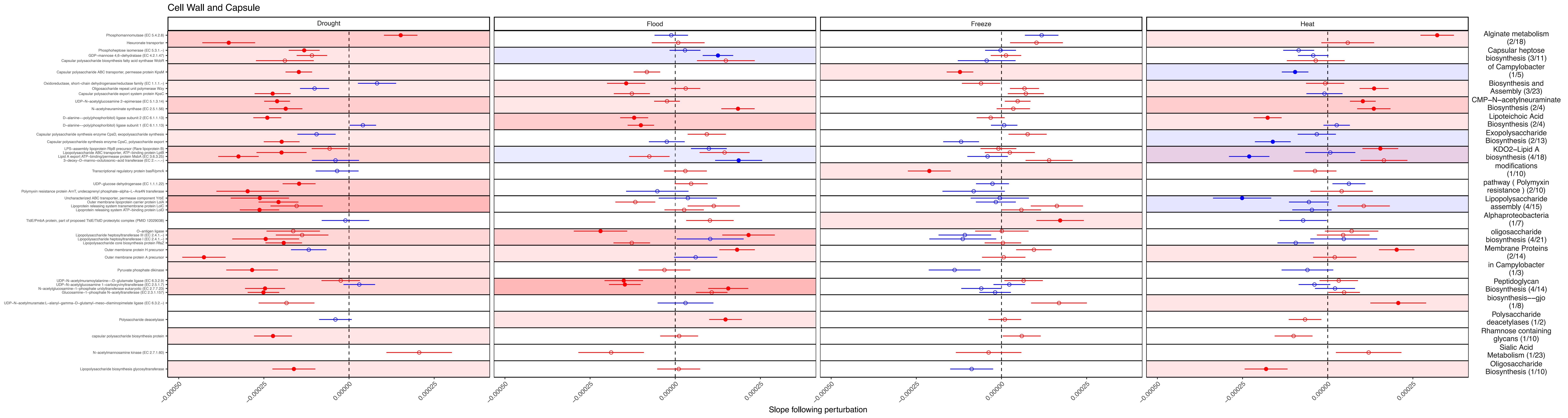

Cell Wall and Capsule
Drought
Flood
Freeze
Heat
Phosphomannomutase (EC 5.4.2.8)
Hexuronate transporter
Phosphoheptose isomerase (EC 5.3.1.–)
GDP–mannose 4,6–dehydratase (EC 4.2.1.47)
Capsular polysaccharide biosynthesis fatty acid synthase WcbR
Capsular polysaccharide ABC transporter, permease protein KpsM
Oxidoreductase, short–chain dehydrogenase/reductase family (EC 1.1.1.–)
Oligosaccharide repeat unit polymerase Wzy
Capsular polysaccharide export system protein KpsC
UDP–N–acetylglucosamine 2–epimerase (EC 5.1.3.14)
N–acetylneuraminate synthase (EC 2.5.1.56)
D–alanine––poly(phosphoribitol) ligase subunit 2 (EC 6.1.1.13)
D–alanine––poly(phosphoribitol) ligase subunit 1 (EC 6.1.1.13)
Capsular polysaccharide synthesis enzyme CpsD, exopolysaccharide synthesis
Capsular polysaccharide synthesis enzyme CpsC, polysaccharide export
LPS–assembly lipoprotein RlpB precursor (Rare lipoprotein B)
Lipopolysaccharide ABC transporter, ATP–binding protein LptB
Lipid A export ATP–binding/permease protein MsbA (EC 3.6.3.25)
3–deoxy–D–manno–octulosonic–acid transferase (EC 2.–.–.–)
Transcriptional regulatory protein basR/pmrA
UDP–glucose dehydrogenase (EC 1.1.1.22)
Polymyxin resistance protein ArnT, undecaprenyl phosphate–alpha–L–Ara4N transferase
Uncharacterized ABC transporter, permease component YrbE
Outer membrane lipoprotein carrier protein LolA
Lipoprotein releasing system transmembrane protein LolC
Lipoprotein releasing system ATP–binding protein LolD
TldE/PmbA protein, part of proposed TldE/TldD proteolytic complex (PMID 12029038)
O–antigen ligase
Lipopolysaccharide heptosyltransferase III (EC 2.4.1.–)
Lipopolysaccharide heptosyltransferase I (EC 2.4.1.–)
Lipopolysaccharide core biosynthesis protein RfaZ
Outer membrane protein H precursor
Outer membrane protein A precursor
Pyruvate phosphate dikinase
UDP–N–acetylmuramoylalanine––D–glutamate ligase (EC 6.3.2.9)
UDP–N–acetylglucosamine 1–carboxyvinyltransferase (EC 2.5.1.7)
N–acetylglucosamine–1–phosphate uridyltransferase eukaryotic (EC 2.7.7.23)
Glucosamine–1–phosphate N–acetyltransferase (EC 2.3.1.157)
UDP–N–acetylmuramate:L–alanyl–gamma–D–glutamyl–meso–diaminopimelate ligase (EC 6.3.2.–)
Polysaccharide deacetylase
capsular polysaccharide biosynthesis protein
N–acetylmannosamine kinase (EC 2.7.1.60)
Lipopolysaccharide biosynthesis glycosyltransferase
Alginate metabolism (2/18)
Capsular heptose biosynthesis (3/11)
of Campylobacter (1/5)
Biosynthesis and Assembly (3/23)
CMP–N–acetylneuraminate Biosynthesis (2/4)
Lipoteichoic Acid Biosynthesis (2/4)
Exopolysaccharide Biosynthesis (2/13)
KDO2–Lipid A biosynthesis (4/18)
modifications (1/10)
pathway (Polymyxin resistance) (2/10)
Lipopolysaccharide assembly (4/15)
Alphaproteobacteria (1/7)
oligosaccharide biosynthesis (4/21)
Membrane Proteins (2/14)
in Campylobacter (1/3)
Peptidoglycan Biosynthesis (4/14)
biosynthesis––gjo (1/8)
Polysaccharide deacetylases (1/2)
Rhamnose containing glycans (1/10)
Sialic Acid Metabolism (1/23)
Oligosaccharide Biosynthesis (1/10)
−0.00050
−0.00025
0.00000
0.00025
Slope following perturbation

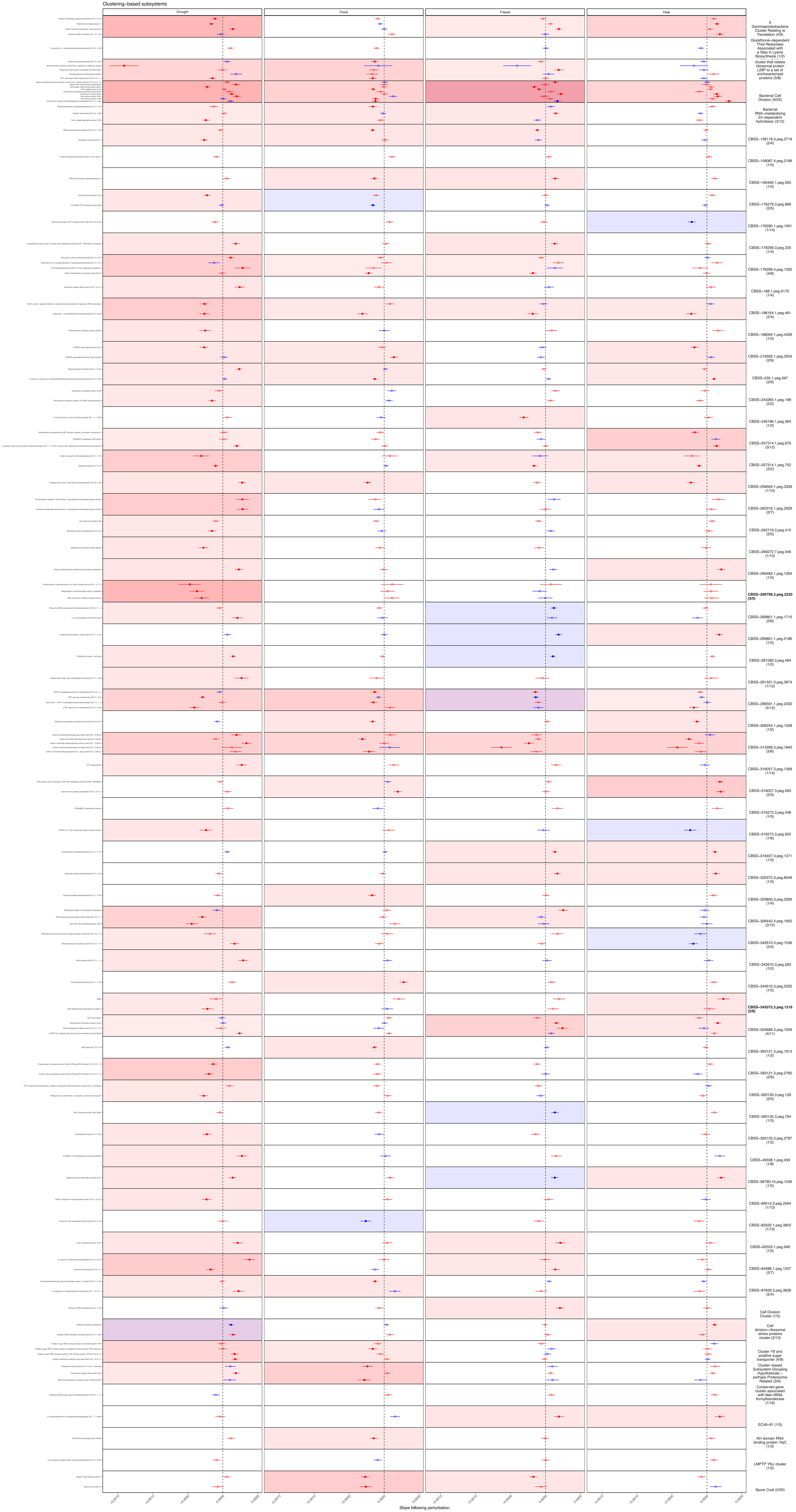
Clustering-based subsystems
Drought
Flood
Freeze
Heat
A Gammaproteobacteria Cluster Relating to Translation (4/9)
Glutathione-dependent Thiol Reductase Associated with a Step in Lysine Biosynthesis (1/2)
cluster that relates ribosomal protein L28P to a set of uncharacterized proteins (5/8)
Bacterial Cell Division (9/25)
Bacterial RNA-metabolizing Zn-dependent hydrolases (3/12)
CBSS-138119.3.peg.2719 (2/4)
CBSS-159087.4.peg.2189 (1/4)
CBSS-160492.1.peg.550 (1/4)
CBSS-176279.3.peg.868 (2/5)
CBSS-176280.1.peg.1561 (1/14)
CBSS-176299.3.peg.235 (1/4)
CBSS-176299.4.peg.1292 (4/8)
CBSS-188.1.peg.6170 (1/4)
CBSS-196164.1.peg.461 (2/4)
CBSS-198094.1.peg.4426 (1/3)
CBSS-216592.1.peg.3534 (2/9)
CBSS-235.1.peg.567 (2/9)
CBSS-243265.1.peg.198 (2/2)
CBSS-246196.1.peg.364 (1/2)
CBSS-257314.1.peg.676 (3/12)
CBSS-257314.1.peg.752 (2/2)
CBSS-258594.1.peg.3339 (1/10)
CBSS-262316.1.peg.2929 (2/7)
CBSS-262719.3.peg.410 (2/5)
CBSS-265072.7.peg.546 (1/10)
CBSS-269482.1.peg.1294 (1/5)
CBSS-269799.3.peg.2220 (3/5)
CBSS-269801.1.peg.1715 (2/6)
CBSS-269801.1.peg.2186 (1/2)
CBSS-281090.3.peg.464 (1/2)
CBSS-291331.3.peg.3674 (1/12)
CBSS-296591.1.peg.2330 (4/12)
CBSS-306254.1.peg.1508 (1/2)
CBSS-314269.3.peg.1840 (5/6)
CBSS-316057.3.peg.1308 (1/14)
CBSS-316057.3.peg.563 (2/3)
CBSS-316273.3.peg.448 (1/5)
CBSS-316273.3.peg.922 (1/6)
CBSS-316407.3.peg.1371 (1/3)
CBSS-320372.3.peg.6046 (1/3)
CBSS-323850.3.peg.3269 (1/4)
CBSS-326442.4.peg.1852 (3/12)
CBSS-342610.3.peg.1536 (2/4)
CBSS-342610.3.peg.283 (1/2)
CBSS-344610.3.peg.2335 (1/5)
CBSS-345072.3.peg.1318 (2/8)
CBSS-350688.3.peg.1509 (4/11)
CBSS-393121.3.peg.1913 (1/2)
CBSS-393121.3.peg.2760 (2/9)
CBSS-393130.3.peg.129 (2/5)
CBSS-393130.3.peg.794 (1/3)
CBSS-393133.3.peg.2787 (1/2)
CBSS-49338.1.peg.459 (1/8)
CBSS-56780.10.peg.1536 (1/5)
CBSS-69014.3.peg.2094 (1/13)
CBSS-83332.1.peg.3803 (1/10)
CBSS-83333.1.peg.946 (1/5)
CBSS-84588.1.peg.1247 (2/7)
CBSS-87626.3.peg.3639 (2/4)
Cell Division Cluster (1/5)
Cell division-ribosomal stress proteins cluster (2/13)
Cluster Ytf and putative sugar transporter (4/9)
Cluster-based Subsystem Grouping Hypotheticals - perhaps Proteosome Related (3/9)
Conserved gene cluster associated with Met-tRNA formyltransferase (1/16)
EC49-61 (1/5)
KH domain RNA binding protein YlqC (1/3)
LMPTP YfkJ cluster (1/3)
Spore Coat (2/20)
-0.0015
-0.0010
-0.0005
0.0000
0.0005
Slope following perturbation

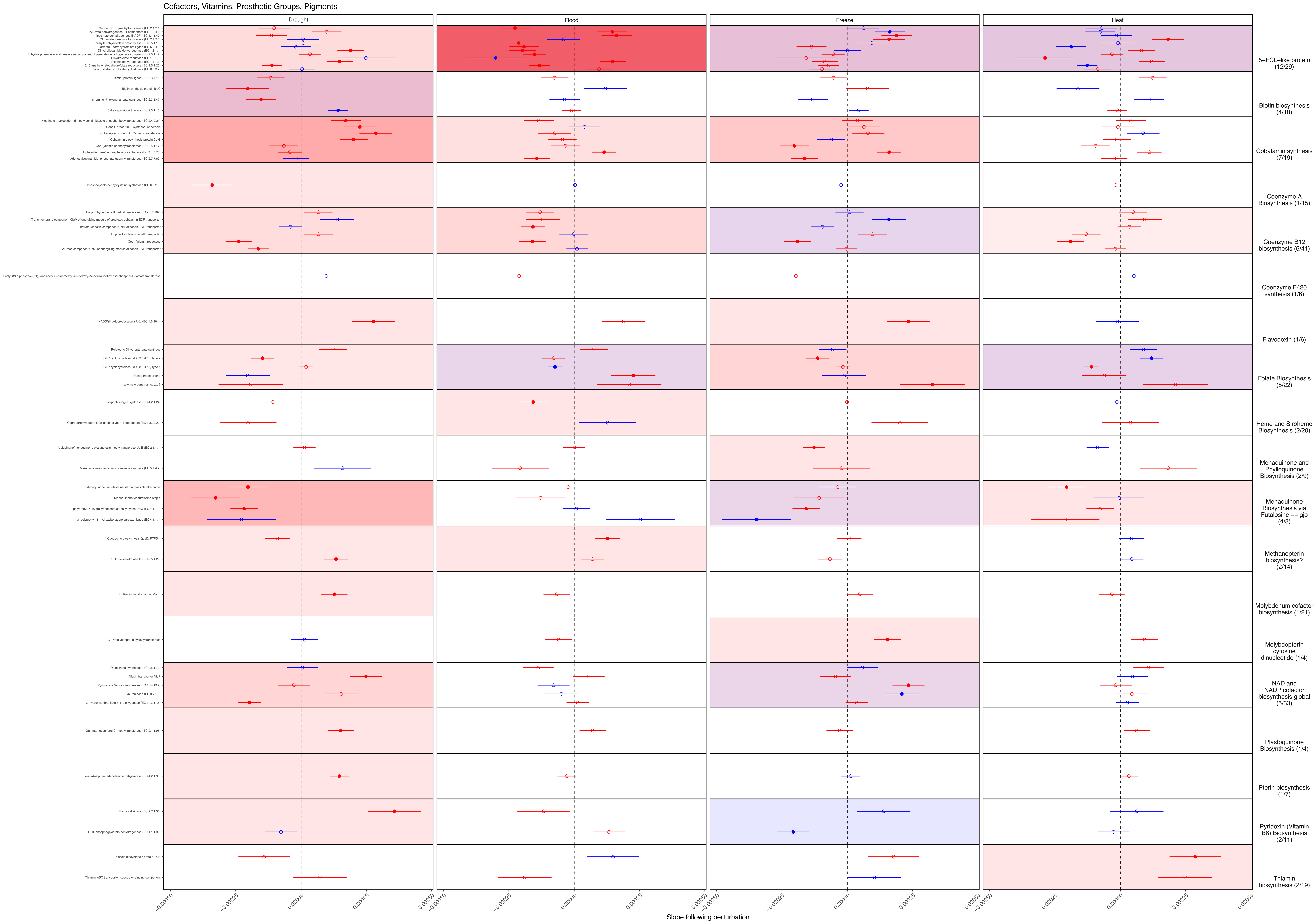

Cofactors, Vitamins, Prosthetic Groups, Pigments
Drought
Flood
Freeze
Heat
Serine hydroxymethyltransferase (EC 2.1.2.1)
Pyruvate dehydrogenase E1 component (EC 1.2.4.1)
Isocitrate dehydrogenase [NADP] (EC 1.1.1.42)
Glutamate formiminotransferase (EC 2.1.2.5)
Formyltetrahydrofolate deformylase (EC 3.5.1.10)
Formate−−tetrahydrofolate ligase (EC 6.3.4.3)
Dihydrolipoamide dehydrogenase (EC 1.8.1.4)
Dihydrolipoamide acetyltransferase component of pyruvate dehydrogenase complex (EC 2.3.1.12)
Dihydrofolate reductase (EC 1.5.1.3)
Alcohol dehydrogenase (EC 1.1.1.1)
5,10−methylenetetrahydrofolate reductase (EC 1.5.1.20)
5−formyltetrahydrofolate cyclo−ligase (EC 6.3.3.2)
Biotin−protein ligase (EC 6.3.4.15)
Biotin synthesis protein bioC
8−amino−7−oxononanoate synthase (EC 2.3.1.47)
3−ketoacyl−CoA thiolase (EC 2.3.1.16)
Nicotinate−nucleotide−−dimethylbenzimidazole phosphoribosyltransferase (EC 2.4.2.21)
Cobalt−precorrin−6 synthase, anaerobic
Cobalt−precorrin−3b C17−methyltransferase
Cobalamin biosynthesis protein CbiG
Cob(I)alamin adenosyltransferase (EC 2.5.1.17)
Alpha−ribazole−5'−phosphate phosphatase (EC 3.1.3.73)
Adenosylcobinamide−phosphate guanylyltransferase (EC 2.7.7.62)
Phosphopantothenoylcysteine synthetase (EC 6.3.2.5)
Uroporphyrinogen−III methyltransferase (EC 2.1.1.107)
Transmembrane component CbrV of energizing module of predicted cobalamin ECF transporter
Substrate−specific component CbiM of cobalt ECF transporter
HupE−UreJ family cobalt transporter
Cob(II)alamin reductase
ATPase component CbiO of energizing module of cobalt ECF transporter
Lactyl (2) diphospho−(5')guanosine:7,8−didemethyl−8−hydroxy−5−deazariboflavin 2−phospho−L−lactate transferase
NAD(P)H oxidoreductase YRKL (EC 1.6.99.−)
Related to Dihydropteroate synthase
GTP cyclohydrolase I (EC 3.5.4.16) type 2
GTP cyclohydrolase I (EC 3.5.4.16) type 1
Folate transporter 3
alternate gene name: yzbB
Porphobilinogen synthase (EC 4.2.1.24)
Coproporphyrinogen III oxidase, oxygen−independent (EC 1.3.99.22)
Ubiquinone/menaquinone biosynthesis methyltransferase UbiE (EC 2.1.1.−)
Menaquinone−specific isochorismate synthase (EC 5.4.4.2)
Menaquinone via futalosine step 4, possible alternative
Menaquinone via futalosine step 4
3−polyprenyl−4−hydroxybenzoate carboxy−lyase UbiX (EC 4.1.1.−)
3−polyprenyl−4−hydroxybenzoate carboxy−lyase (EC 4.1.1.−)
Queuosine biosynthesis QueD, PTPS−I
GTP cyclohydrolase III (EC 3.5.4.29)
DNA−binding domain of ModE
CTP:molybdopterin cytidylyltransferase
Quinolinate synthetase (EC 2.5.1.72)
Niacin transporter NiaP
Kynurenine 3−monooxygenase (EC 1.14.13.9)
Kynureninase (EC 3.7.1.3)
3−hydroxyanthranilate 3,4−dioxygenase (EC 1.13.11.6)
Gamma−tocopherol C−methyltransferase (EC 2.1.1.95)
Pterin−4−alpha−carbinolamine dehydratase (EC 4.2.1.96)
Pyridoxal kinase (EC 2.7.1.35)
D−3−phosphoglycerate dehydrogenase (EC 1.1.1.95)
Thiazole biosynthesis protein ThiH
Thiamin ABC transporter, substrate−binding component
5−FCL−like protein (12/29)
Biotin biosynthesis (4/18)
Cobalamin synthesis (7/19)
Coenzyme A Biosynthesis (1/15)
Coenzyme B12 biosynthesis (6/41)
Coenzyme F420 synthesis (1/6)
Flavodoxin (1/6)
Folate Biosynthesis (5/22)
Heme and Siroheme Biosynthesis (2/20)
Menaquinone and Phylloquinone Biosynthesis (2/9)
Menaquinone Biosynthesis via Futalosine — gjo (4/8)
Methanopterin biosynthesis2 (2/14)
Molybdenum cofactor biosynthesis (1/21)
Molybdopterin cytosine dinucleotide (1/4)
NAD and NADP cofactor biosynthesis global (5/33)
Plastoquinone Biosynthesis (1/4)
Pterin biosynthesis (1/7)
Pyridoxin (Vitamin B6) Biosynthesis (2/11)
Thiamin biosynthesis (2/19)
−0.00050
−0.00025
0.00000
0.00025
0.00050
Slope following perturbation

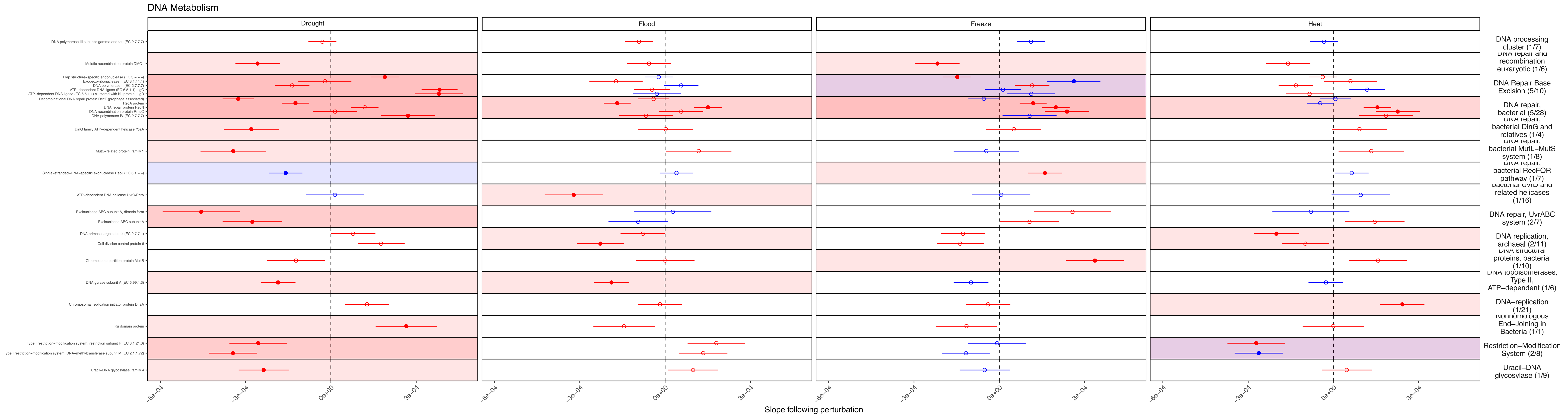
DNA Metabolism
Drought
Flood
Freeze
Heat
DNA polymerase III subunits gamma and tau (EC 2.7.7.7)
Meiotic recombination protein DMC1
Flap structure–specific endonuclease (EC 3.–.–.–)
Exodeoxyribonuclease I (EC 3.1.11.1)
DNA polymerase II (EC 2.7.7.7)
ATP–dependent DNA ligase (EC 6.5.1.1) LigC
ATP–dependent DNA ligase (EC 6.5.1.1) clustered with Ku protein, LigD
Recombinational DNA repair protein RecT (prophage associated)
RecA protein
DNA repair protein RecN
DNA recombination protein RmuC
DNA polymerase IV (EC 2.7.7.7)
DinG family ATP–dependent helicase YoaA
MutS–related protein, family 1
Single–stranded–DNA–specific exonuclease RecJ (EC 3.1.–.–)
ATP–dependent DNA helicase UvrD/PcrA
Excinuclease ABC subunit A, dimeric form
Excinuclease ABC subunit A
DNA primase large subunit (EC 2.7.7.–)
Cell division control protein 6
Chromosome partition protein MukB
DNA gyrase subunit A (EC 5.99.1.3)
Chromosomal replication initiator protein DnaA
Ku domain protein
Type I restriction–modification system, restriction subunit R (EC 3.1.21.3)
Type I restriction–modification system, DNA–methyltransferase subunit M (EC 2.1.1.72)
Uracil–DNA glycosylase, family 4
DNA processing cluster (1/7)
DNA repair and recombination eukaryotic (1/6)
DNA Repair Base Excision (5/10)
DNA repair, bacterial (5/28)
DNA repair, bacterial DinG and relatives (1/4)
DNA repair, bacterial MutL–MutS system (1/8)
DNA repair, bacterial RecFOR pathway (1/7)
bacterial UvrD and related helicases (1/16)
DNA repair, UvrABC system (2/7)
DNA replication, archaeal (2/11)
DNA structural proteins, bacterial (1/10)
DNA topoisomerases, Type II, ATP–dependent (1/6)
DNA–replication (1/21)
Nonhomologous End–Joining in Bacteria (1/1)
Restriction–Modification System (2/8)
Uracil–DNA glycosylase (1/9)
–6e–04
–3e–04
0e+00
3e–04
Slope following perturbation

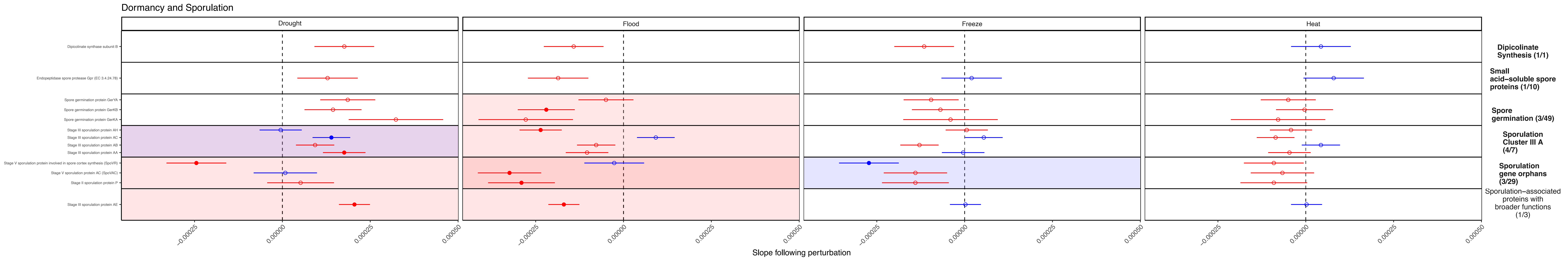
Dormancy and Sporulation
Drought
Flood
Freeze
Heat
Dipicolinate synthase subunit B
Endopeptidase spore protease Gpr (EC 3.4.24.78)
Spore germination protein GerYA
Spore germination protein GerKB
Spore germination protein GerKA
Stage III sporulation protein AH
Stage III sporulation protein AC
Stage III sporulation protein AB
Stage III sporulation protein AA
Stage V sporulation protein involved in spore cortex synthesis (SpoVR)
Stage V sporulation protein AC (SpoVAC)
Stage II sporulation protein P
Stage III sporulation protein AE
Dipicolinate Synthesis (1/1)
Small acid−soluble spore proteins (1/10)
Spore germination (3/49)
Sporulation Cluster III A (4/7)
Sporulation gene orphans (3/29)
Sporulation−associated proteins with broader functions (1/3)
−0.00025
0.00000
0.00025
0.00050
Slope following perturbation

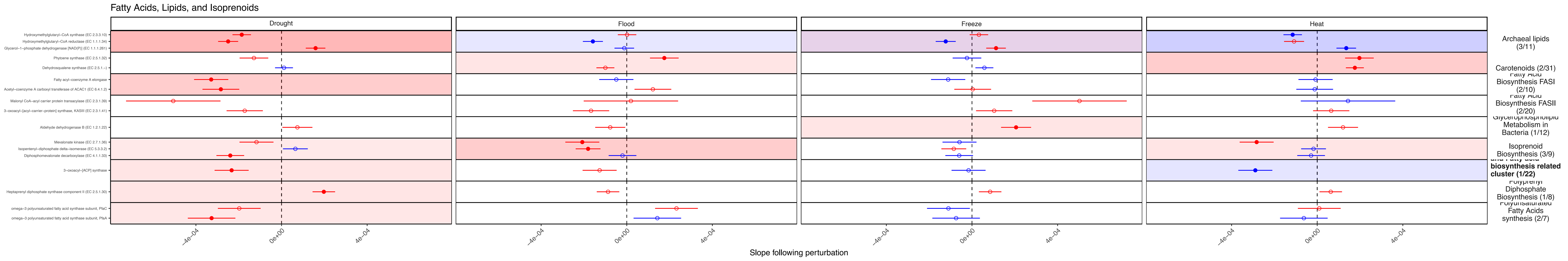

Fatty Acids, Lipids, and Isoprenoids
Drought
Flood
Freeze
Heat
Hydroxymethylglutaryl–CoA synthase (EC 2.3.3.10)
Hydroxymethylglutaryl–CoA reductase (EC 1.1.1.34)
Glycerol–1–phosphate dehydrogenase [NAD(P)] (EC 1.1.1.261)
Phytoene synthase (EC 2.5.1.32)
Dehydrosqualene synthase (EC 2.5.1.–)
Fatty acyl–coenzyme A elongase
Acetyl–coenzyme A carboxyl transferase of ACAC1 (EC 6.4.1.2)
Malonyl CoA–acyl carrier protein transacylase (EC 2.3.1.39)
3–oxoacyl–[acyl–carrier–protein] synthase, KASIII (EC 2.3.1.41)
Aldehyde dehydrogenase B (EC 1.2.1.22)
Mevalonate kinase (EC 2.7.1.36)
Isopentenyl–diphosphate delta–isomerase (EC 5.3.3.2)
Diphosphomevalonate decarboxylase (EC 4.1.1.33)
3–oxoacyl–[ACP] synthase
Heptaprenyl diphosphate synthase component II (EC 2.5.1.30)
omega–3 polyunsaturated fatty acid synthase subunit, PfaC
omega–3 polyunsaturated fatty acid synthase subunit, PfaA
Archaeal lipids (3/11)
Carotenoids (2/31)
Fatty Acid Biosynthesis FASI (2/10)
Fatty Acid Biosynthesis FASII (2/20)
Glycerophospholipid Metabolism in Bacteria (1/12)
Isoprenoid Biosynthesis (3/9)
and Fatty acid biosynthesis related cluster (1/22)
Polyprenyl Diphosphate Biosynthesis (1/8)
Polyunsaturated Fatty Acids synthesis (2/7)
–4e–04
0e+00
4e–04
Slope following perturbation

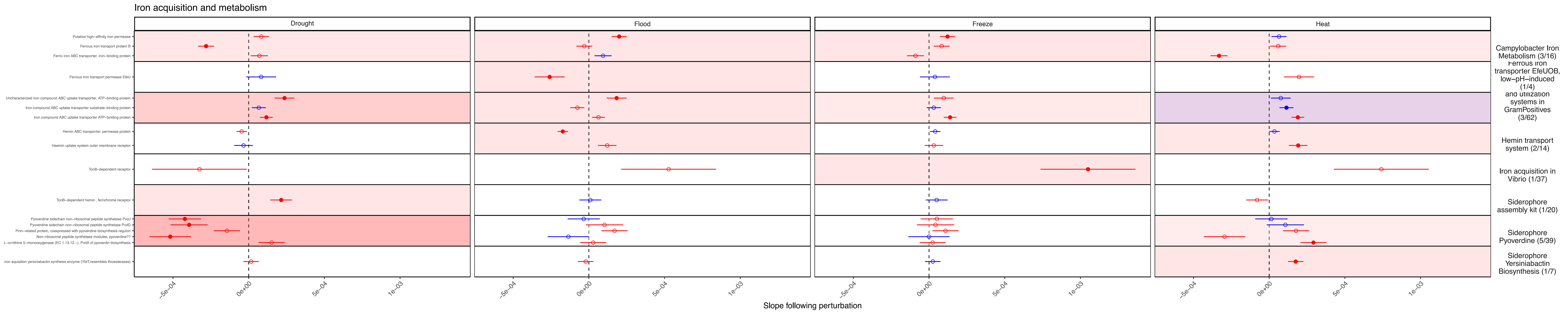
Iron acquisition and metabolism
Drought
Flood
Freeze
Heat
Putative high–affinity iron permease
Ferrous iron transport protein B
Ferric iron ABC transporter, iron–binding protein
Ferrous iron transport permease EfeU
Uncharacterized iron compound ABC uptake transporter, ATP–binding protein
Iron compound ABC uptake transporter substrate–binding protein
Iron compound ABC uptake transporter ATP–binding protein
Hemin ABC transporter, permease protein
Haemin uptake system outer membrane receptor
TonB–dependent receptor
TonB–dependent hemin , ferrichrome receptor
Pyoverdine sidechain non–ribosomal peptide synthetase PvdJ
Pyoverdine sidechain non–ribosomal peptide synthetase PvdD
Pirin–related protein, coexpressed with pyoverdine biosynthesis regulon
Non–ribosomal peptide synthetase modules, pyoverdine??
L–ornithine 5–monooxygenase (EC 1.13.12.–), PvdA of pyoverdin biosynthesis
iron aquisition yersiniabactin synthesis enzyme (YbtT,resembles thioesterases)
Campylobacter Iron Metabolism (3/16)
transporter EfeUOB, low–pH–induced (1/4)
systems in GramPositives (3/62)
Hemin transport system (2/14)
Iron acquisition in Vibrio (1/37)
Siderophore assembly kit (1/20)
Siderophore Pyoverdine (5/39)
Siderophore Yersiniabactin Biosynthesis (1/7)
–5e–04
0e+00
5e–04
1e–03
Slope following perturbation

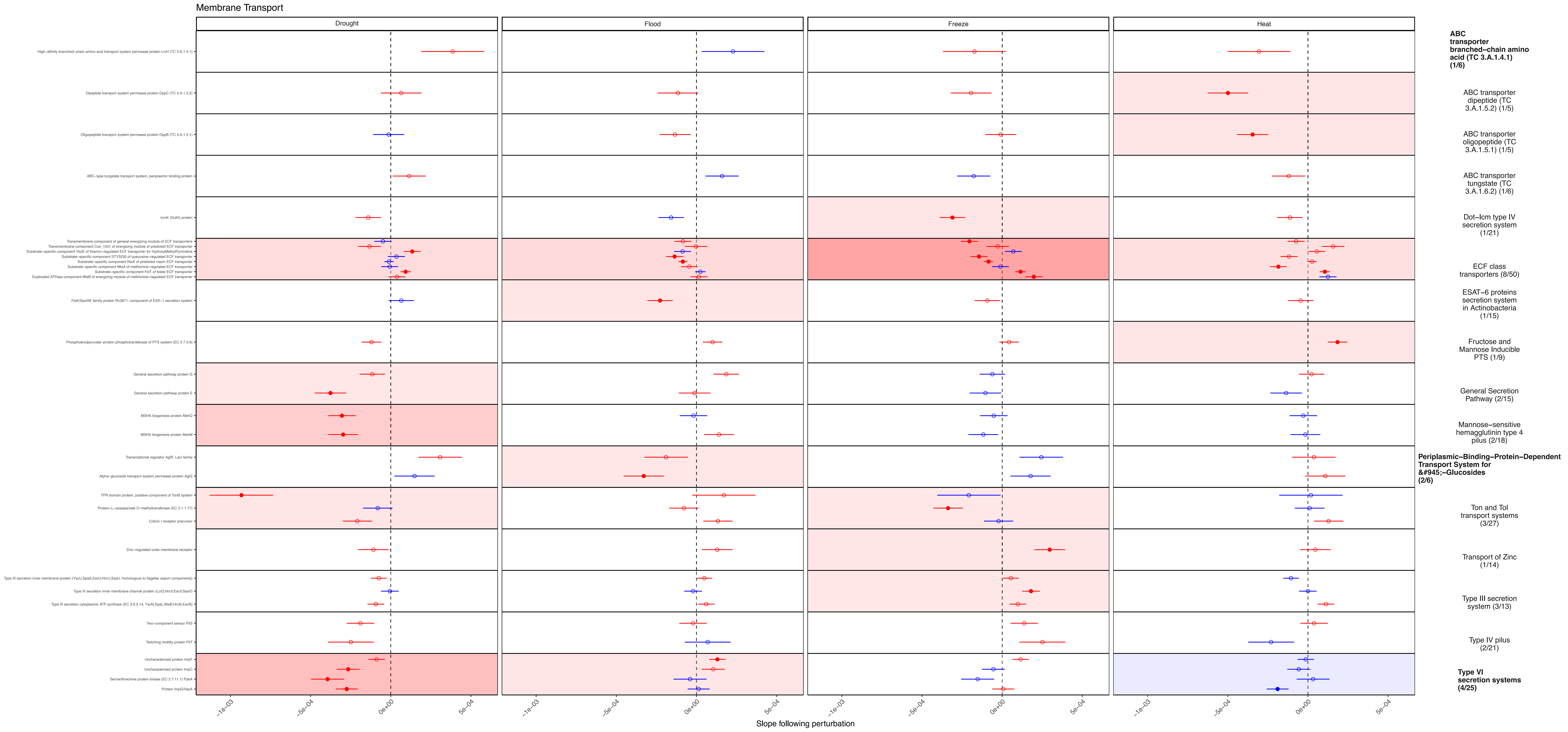

Membrane Transport
Drought
Flood
Freeze
Heat
High−affinity branched−chain amino acid transport system permease protein LivH (TC 3.A.1.4.1)
Dipeptide transport system permease protein DppC (TC 3.A.1.5.2)
Oligopeptide transport system permease protein OppB (TC 3.A.1.5.1)
ABC−type tungstate transport system, periplasmic binding protein
IcmK (DotH) protein
Transmembrane component of general energizing module of ECF transporters
Transmembrane component Cce_1531 of energizing module of predicted ECF transporter
Substrate−specific component YkoE of thiamin−regulated ECF transporter for HydroxyMethylPyrimidine
Substrate−specific component STY3230 of queuosine−regulated ECF transporter
Substrate−specific component NiaX of predicted niacin ECF transporter
Substrate−specific component MtsA of methionine−regulated ECF transporter
Substrate−specific component FolT of folate ECF transporter
Duplicated ATPase component MtsB of energizing module of methionine−regulated ECF transporter
FtsK/SpoIIIE family protein Rv3871, component of ESX−1 secretion system
Phosphoenolpyruvate−protein phosphotransferase of PTS system (EC 2.7.3.9)
General secretion pathway protein G
General secretion pathway protein E
MSHA biogenesis protein MshQ
MSHA biogenesis protein MshM
Transcriptional regulator AglR, LacI family
Alpha−glucoside transport system permease protein AglG
TPR domain protein, putative component of TonB system
Protein−L−isoaspartate O−methyltransferase (EC 2.1.1.77)
Colicin I receptor precursor
Zinc−regulated outer membrane receptor
Type III secretion inner membrane protein (YscU,SpaS,EscU,HrcU,SsaU, homologous to flagellar export components)
Type III secretion inner membrane channel protein (LcrD,HrcV,EscV,SsaV)
Type III secretion cytoplasmic ATP synthase (EC 3.6.3.14, YscN,SpaL,MxiB,HrcN,EscN)
Two−component sensor PilS
Twitching motility protein PilT
Uncharacterized protein ImpF
Uncharacterized protein ImpC
Serine/threonine protein kinase (EC 2.7.11.1) PpkA
Protein ImpG/VasA
ABC transporter branched−chain amino acid (TC 3.A.1.4.1) (1/6)
ABC transporter dipeptide (TC 3.A.1.5.2) (1/5)
ABC transporter oligopeptide (TC 3.A.1.5.1) (1/5)
ABC transporter tungstate (TC 3.A.1.6.2) (1/6)
Dot−Icm type IV secretion system (1/21)
ECF class transporters (8/50)
ESAT−6 proteins secretion system in Actinobacteria (1/15)
Fructose and Mannose Inducible PTS (1/9)
General Secretion Pathway (2/15)
Mannose−sensitive hemagglutinin type 4 pilus (2/18)
Periplasmic−Binding−Protein−Dependent Transport System for α−Glucosides (2/6)
Ton and Tol transport systems (3/27)
Transport of Zinc (1/14)
Type III secretion system (3/13)
Type IV pilus (2/21)
Type VI secretion systems (4/25)
−1e−03
−5e−04
0e+00
5e−04
Slope following perturbation

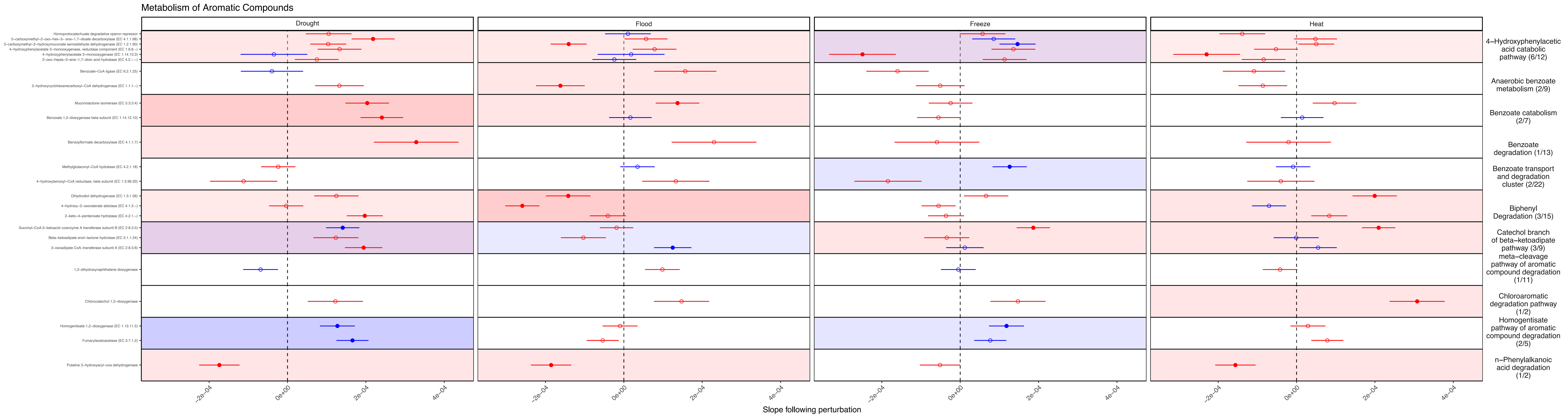

Metabolism of Aromatic Compounds
Drought
Flood
Freeze
Heat
Homoprotocatechuate degradative operon repressor
5–carboxymethyl–2–oxo–hex–3– ene–1,7–dioate decarboxylase (EC 4.1.1.68)
5–carboxymethyl–2–hydroxymuconate semialdehyde dehydrogenase (EC 1.2.1.60)
4–hydroxyphenylacetate 3–monooxygenase, reductase component (EC 1.6.8.–)
4–hydroxyphenylacetate 3–monooxygenase (EC 1.14.13.3)
2–oxo–hepta–3–ene–1,7–dioic acid hydratase (EC 4.2.–.–)
Benzoate–CoA ligase (EC 6.2.1.25)
2–hydroxycyclohexanecarboxyl–CoA dehydrogenase (EC 1.1.1.–)
Muconolactone isomerase (EC 5.3.3.4)
Benzoate 1,2–dioxygenase beta subunit (EC 1.14.12.10)
Benzoylformate decarboxylase (EC 4.1.1.7)
Methylglutaconyl–CoA hydratase (EC 4.2.1.18)
4–hydroxybenzoyl–CoA reductase, beta subunit (EC 1.3.99.20)
Dihydrodiol dehydrogenase (EC 1.3.1.56)
4–hydroxy–2–oxovalerate aldolase (EC 4.1.3.–)
2–keto–4–pentenoate hydratase (EC 4.2.1.–)
Succinyl–CoA:3–ketoacid–coenzyme A transferase subunit B (EC 2.8.3.5)
Beta–ketoadipate enol–lactone hydrolase (EC 3.1.1.24)
3–oxoadipate CoA–transferase subunit A (EC 2.8.3.6)
1,2–dihydroxynaphthalene dioxygenase
Chlorocatechol 1,2–dioxygenase
Homogentisate 1,2–dioxygenase (EC 1.13.11.5)
Fumarylacetoacetase (EC 3.7.1.2)
Putative 3–hydroxyacyl–coa dehydrogenase
4–Hydroxyphenylacetic acid catabolic pathway (6/12)
Anaerobic benzoate metabolism (2/9)
Benzoate catabolism (2/7)
Benzoate degradation (1/13)
Benzoate transport and degradation cluster (2/22)
Biphenyl Degradation (3/15)
Catechol branch of beta–ketoadipate pathway (3/9)
meta–cleavage pathway of aromatic compound degradation (1/11)
Chloroaromatic degradation pathway (1/2)
Homogentisate pathway of aromatic compound degradation (2/5)
n–Phenylalkanoic acid degradation (1/2)
−2e−04
0e+00
2e−04
4e−04
Slope following perturbation

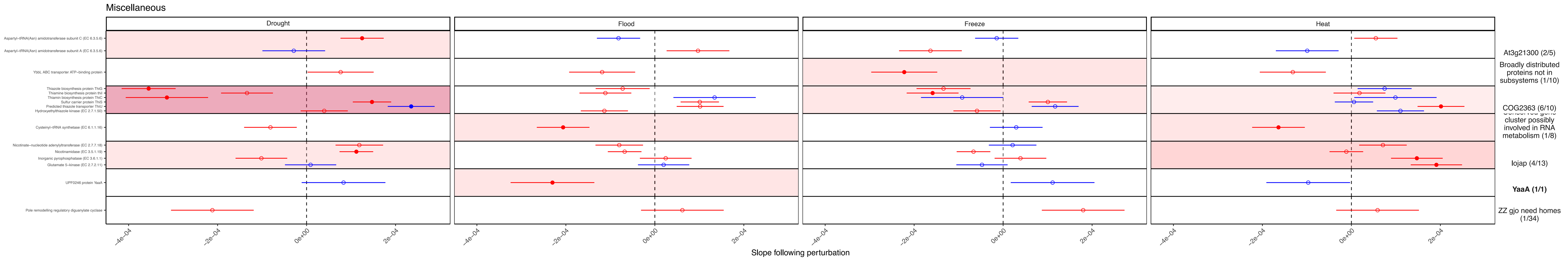

Miscellaneous
Drought
Flood
Freeze
Heat
Aspartyl–tRNA(Asn) amidotransferase subunit C (EC 6.3.5.6)
Aspartyl–tRNA(Asn) amidotransferase subunit A (EC 6.3.5.6)
YbbL ABC transporter ATP–binding protein
Thiazole biosynthesis protein ThiG
Thiamine biosynthesis protein thiI
Thiamin biosynthesis protein ThiC
Sulfur carrier protein ThiS
Predicted thiazole transporter ThiU
Hydroxyethylthiazole kinase (EC 2.7.1.50)
Cysteinyl–tRNA synthetase (EC 6.1.1.16)
Nicotinate–nucleotide adenylyltransferase (EC 2.7.7.18)
Nicotinamidase (EC 3.5.1.19)
Inorganic pyrophosphatase (EC 3.6.1.1)
Glutamate 5–kinase (EC 2.7.2.11)
UPF0246 protein YaaA
Pole remodelling regulatory diguanylate cyclase
At3g21300 (2/5)
Broadly distributed proteins not in subsystems (1/10)
COG2363 (6/10)
Conserved gene cluster possibly involved in RNA metabolism (1/8)
Iojap (4/13)
YaaA (1/1)
ZZ gjo need homes (1/34)
−4e−04
−2e−04
0e+00
2e−04
Slope following perturbation

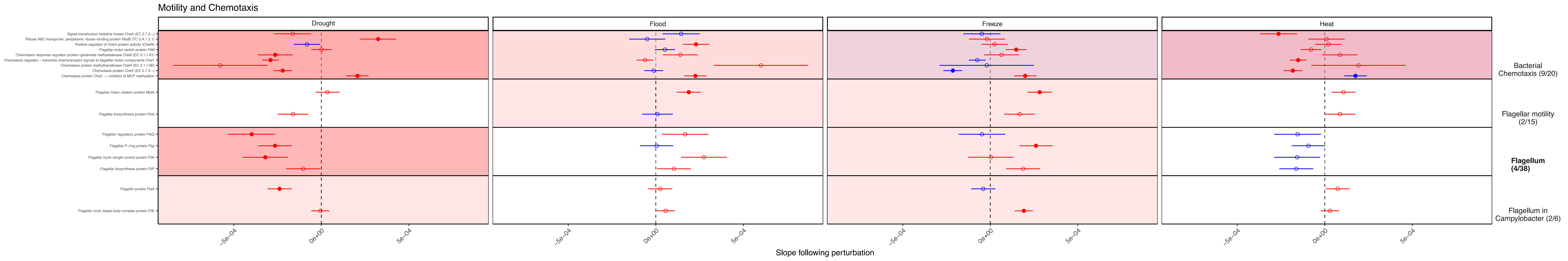

Motility and Chemotaxis
Drought
Flood
Freeze
Heat
Signal transduction histidine kinase CheA (EC 2.7.3.–)
Ribose ABC transporter, periplasmic ribose–binding protein RbsB (TC 3.A.1.2.1)
Positive regulator of CheA protein activity (CheW)
Flagellar motor switch protein FliM
Chemotaxis response regulator protein–glutamate methylesterase CheB (EC 3.1.1.61)
Chemotaxis regulator – transmits chemoreceptor signals to flagelllar motor components CheY
Chemotaxis protein methyltransferase CheR (EC 2.1.1.80)
Chemotaxis protein CheV (EC 2.7.3.–)
Chemotaxis protein CheC –– inhibitor of MCP methylation
Flagellar motor rotation protein MotA
Flagellar biosynthesis protein FlhA
Flagellar regulatory protein FleQ
Flagellar P–ring protein FlgI
Flagellar hook–length control protein FliK
Flagellar biosynthesis protein FliP
Flagellin protein FlaA
Flagellar hook–basal body complex protein FliE
Bacterial Chemotaxis (9/20)
Flagellar motility (2/15)
Flagellum (4/38)
Flagellum in Campylobacter (2/6)
−5e−04
0e+00
5e−04
Slope following perturbation

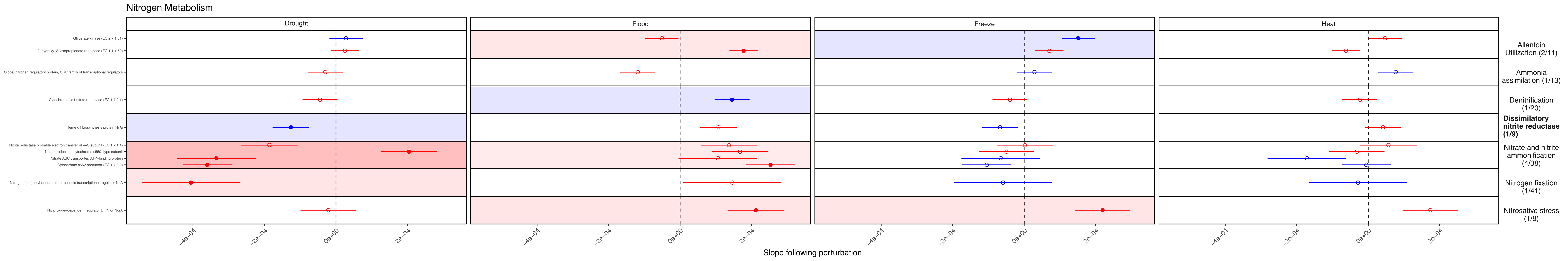

Nitrogen Metabolism
Drought
Flood
Freeze
Heat
Glycerate kinase (EC 2.7.1.31)
2–hydroxy–3–oxopropionate reductase (EC 1.1.1.60)
Global nitrogen regulatory protein, CRP family of transcriptional regulators
Cytochrome cd1 nitrite reductase (EC:1.7.2.1)
Heme d1 biosynthesis protein NirG
Nitrite reductase probable electron transfer 4Fe–S subunit (EC 1.7.1.4)
Nitrate reductase cytochrome c550–type subunit
Nitrate ABC transporter, ATP–binding protein
Cytochrome c552 precursor (EC 1.7.2.2)
Nitrogenase (molybdenum–iron)–specific transcriptional regulator NifA
Nitric oxide–dependent regulator DnrN or NorA
Allantoin Utilization (2/11)
Ammonia assimilation (1/13)
Denitrification (1/20)
Dissimilatory nitrite reductase (1/9)
Nitrate and nitrite ammonification (4/38)
Nitrogen fixation (1/41)
Nitrosative stress (1/8)
−4e−04
−2e−04
0e+00
2e−04
Slope following perturbation

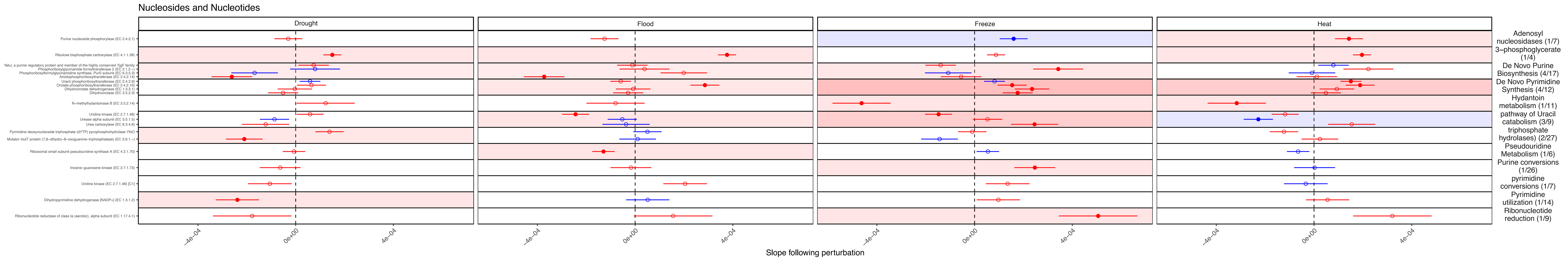
Nucleosides and Nucleotides
Drought
Flood
Freeze
Heat
Purine nucleoside phosphorylase (EC 2.4.2.1)
Ribulose bisphosphate carboxylase (EC 4.1.1.39)
YabJ, a purine regulatory protein and member of the highly conserved YjgF family
Phosphoribosylglycinamide formyltransferase 2 (EC 2.1.2.–)
Phosphoribosylformylglycinamidine synthase, PurS subunit (EC 6.3.5.3)
Amidophosphoribosyltransferase (EC 2.4.2.14)
Uracil phosphoribosyltransferase (EC 2.4.2.9)
Orotate phosphoribosyltransferase (EC 2.4.2.10)
Dihydroorotate dehydrogenase (EC 1.3.3.1)
Dihydroorotase (EC 3.5.2.3)
N–methylhydantoinase B (EC 3.5.2.14)
Uridine kinase (EC 2.7.1.48)
Urease alpha subunit (EC 3.5.1.5)
Urea carboxylase (EC 6.3.4.6)
Pyrimidine deoxynucleoside triphosphate (dYTP) pyrophosphohydrolase YfoO
Mutator mutT protein (7,8–dihydro–8–oxoguanine–triphosphatase) (EC 3.6.1.–)
Ribosomal small subunit pseudouridine synthase A (EC 4.2.1.70)
Inosine–guanosine kinase (EC 2.7.1.73)
Uridine kinase (EC 2.7.1.48) [C1]
Dihydropyrimidine dehydrogenase [NADP+] (EC 1.3.1.2)
Ribonucleotide reductase of class Ia (aerobic), alpha subunit (EC 1.17.4.1)
Adenosyl nucleosidases (1/7)
3–phosphoglycerate (1/4)
De Novo Purine Biosynthesis (4/17)
De Novo Pyrimidine Synthesis (4/12)
Hydantoin metabolism (1/11)
pathway of Uracil catabolism (3/9)
triphosphate hydrolases) (2/27)
Pseudouridine Metabolism (1/6)
Purine conversions (1/26)
pyrimidine conversions (1/7)
Pyrimidine utilization (1/14)
Ribonucleotide reduction (1/9)
−4e−04
0e+00
4e−04
Slope following perturbation

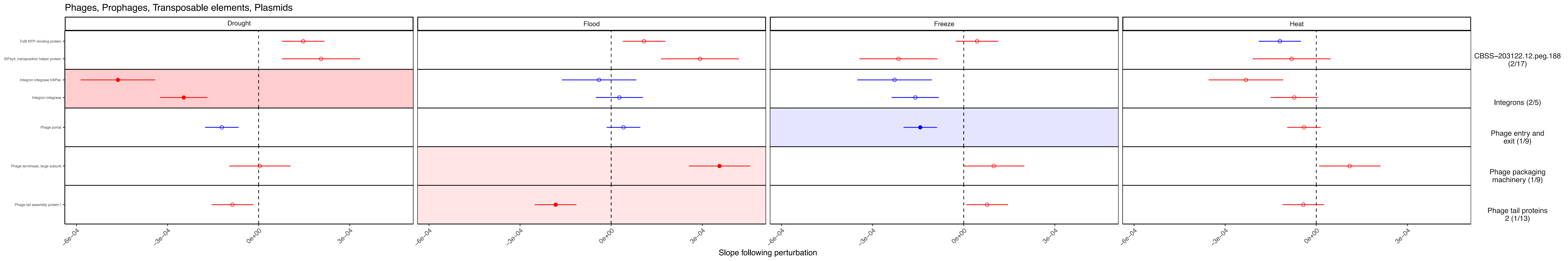
Phages, Prophages, Transposable elements, Plasmids
Drought
Flood
Freeze
Heat
TniB NTP−binding protein
ISPsy4, transposition helper protein
Integron integrase IntIPac
Integron integrase
Phage portal
Phage terminase, large subunit
Phage tail assembly protein I
CBSS−203122.12.peg.188 (2/17)
Integrons (2/5)
Phage entry and exit (1/9)
Phage packaging machinery (1/9)
Phage tail proteins 2 (1/13)
−6e−04
−3e−04
0e+00
3e−04
Slope following perturbation

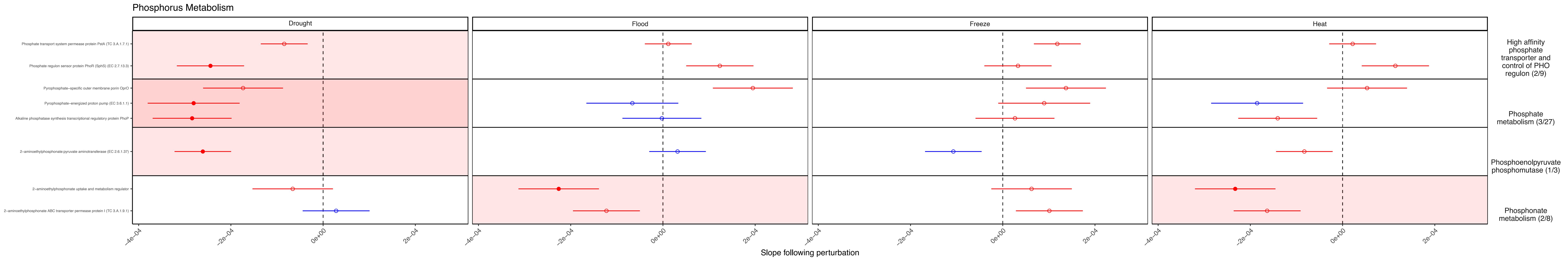
Phosphorus Metabolism
Drought
Flood
Freeze
Heat
Phosphate transport system permease protein PstA (TC 3.A.1.7.1)
Phosphate regulon sensor protein PhoR (SphS) (EC 2.7.13.3)
Pyrophosphate–specific outer membrane porin OprO
Pyrophosphate–energized proton pump (EC 3.6.1.1)
Alkaline phosphatase synthesis transcriptional regulatory protein PhoP
2–aminoethylphosphonate:pyruvate aminotransferase (EC 2.6.1.37)
2–aminoethylphosphonate uptake and metabolism regulator
2–aminoethylphosphonate ABC transporter permease protein I (TC 3.A.1.9.1)
High affinity phosphate transporter and control of PHO regulon (2/9)
Phosphate metabolism (3/27)
Phosphoenolpyruvate phosphomutase (1/3)
Phosphonate metabolism (2/8)
−4e−04
−2e−04
0e+00
2e−04
Slope following perturbation

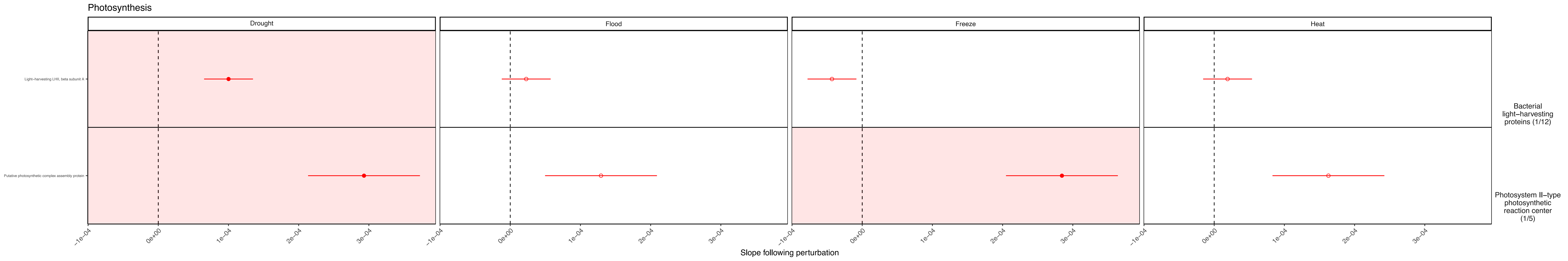

Photosynthesis
Drought
Flood
Freeze
Heat
Light−harvesting LHII, beta subunit A
Putative photosynthetic complex assembly protein
Bacterial light−harvesting proteins (1/12)
Photosystem II−type photosynthetic reaction center (1/5)
−1e−04
0e+00
1e−04
2e−04
3e−04
Slope following perturbation

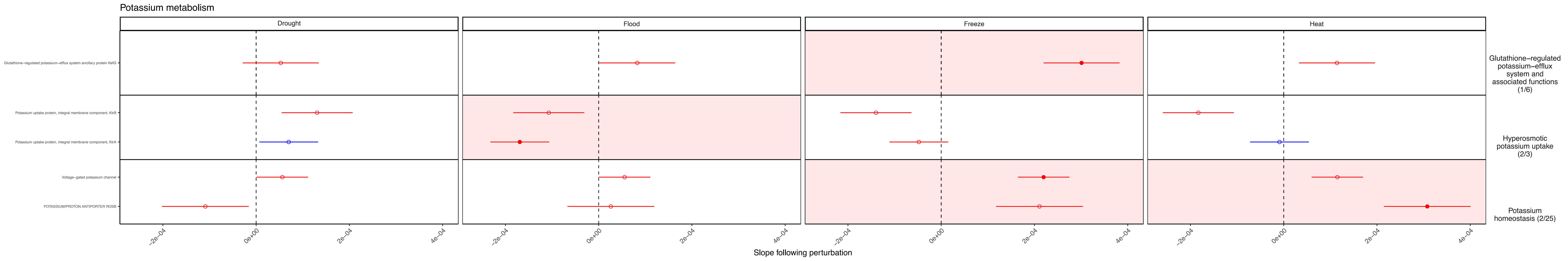
Potassium metabolism
Drought
Flood
Freeze
Heat
Glutathione−regulated potassium−efflux system ancillary protein KefG
Potassium uptake protein, integral membrane component, KtrB
Potassium uptake protein, integral membrane component, KtrA
Voltage−gated potassium channel
POTASSIUM/PROTON ANTIPORTER ROSB
Glutathione−regulated potassium−efflux system and associated functions (1/6)
Hyperosmotic potassium uptake (2/3)
Potassium homeostasis (2/25)
−2e−04
0e+00
2e−04
4e−04
Slope following perturbation

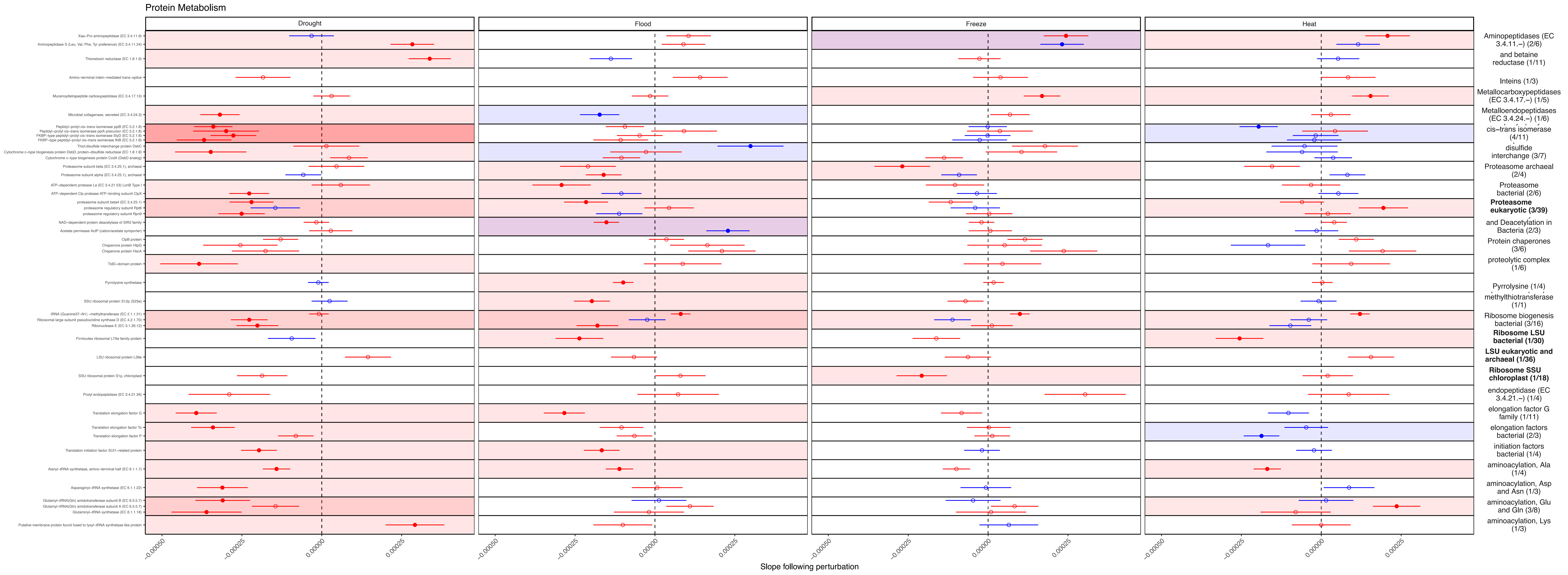
Protein Metabolism
Drought
Flood
Freeze
Heat
Xaa–Pro aminopeptidase (EC 3.4.11.9)
Aminopeptidase S (Leu, Val, Phe, Tyr preference) (EC 3.4.11.24)
Thioredoxin reductase (EC 1.8.1.9)
Amino–terminal intein–mediated trans–splice
Muramoyltetrapeptide carboxypeptidase (EC 3.4.17.13)
Microbial collagenase, secreted (EC 3.4.24.3)
Peptidyl–prolyl cis–trans isomerase ppiB (EC 5.2.1.8)
Peptidyl–prolyl cis–trans isomerase ppiA precursor (EC 5.2.1.8)
FKBP–type peptidyl–prolyl cis–trans isomerase SlyD (EC 5.2.1.8)
FKBP–type peptidyl–prolyl cis–trans isomerase fkIB (EC 5.2.1.8)
Thiol:disulfide interchange protein DsbC
Cytochrome c–type biogenesis protein DsbD, protein–disulfide reductase (EC 1.8.1.8)
Cytochrome c–type biogenesis protein CcdA (DsbD analog)
Proteasome subunit beta (EC 3.4.25.1), archaeal
Proteasome subunit alpha (EC 3.4.25.1), archaeal
ATP–dependent protease La (EC 3.4.21.53) LonB Type I
ATP–dependent Clp protease ATP–binding subunit ClpX
proteasome subunit beta4 (EC 3.4.25.1)
proteasome regulatory subunit Rpt6
proteasome regulatory subunit Rpn9
NAD–dependent protein deacetylase of SIR2 family
Acetate permease ActP (cation/acetate symporter)
ClpB protein
Chaperone protein HtpG
Chaperone protein HscA
TldD–domain protein
Pyrrolysine synthetase
SSU ribosomal protein S12p (S23e)
tRNA (Guanine37–N1) –methyltransferase (EC 2.1.1.31)
Ribosomal large subunit pseudouridine synthase D (EC 4.2.1.70)
Ribonuclease E (EC 3.1.26.12)
Firmicutes ribosomal L7Ae family protein
LSU ribosomal protein L39e
SSU ribosomal protein S1p, chloroplast
Prolyl endopeptidase (EC 3.4.21.26)
Translation elongation factor G
Translation elongation factor Tu
Translation elongation factor P
Translation initiation factor SUI1–related protein
Alanyl–tRNA synthetase, amino–terminal half (EC 6.1.1.7)
Asparaginyl–tRNA synthetase (EC 6.1.1.22)
Glutamyl–tRNA(Gln) amidotransferase subunit B (EC 6.3.5.7)
Glutamyl–tRNA(Gln) amidotransferase subunit A (EC 6.3.5.7)
Glutaminyl–tRNA synthetase (EC 6.1.1.18)
Putative membrane protein found fused to lysyl–tRNA synthetase like protein
Aminopeptidases (EC 3.4.11.–) (2/6)
and betaine reductase (1/11)
Inteins (1/3)
Metallocarboxypeptidases (EC 3.4.17.–) (1/5)
Metalloendopeptidases (EC 3.4.24.–) (1/6)
cis–trans isomerase (4/11)
disulfide interchange (3/7)
Proteasome archaeal (2/4)
Proteasome bacterial (2/6)
Proteasome eukaryotic (3/39)
and Deacetylation in Bacteria (2/3)
Protein chaperones (3/6)
proteolytic complex (1/6)
Pyrrolysine (1/4)
methylthiotransferase (1/1)
Ribosome biogenesis bacterial (3/16)
Ribosome LSU bacterial (1/30)
LSU eukaryotic and archaeal (1/36)
Ribosome SSU chloroplast (1/18)
endopeptidase (EC 3.4.21.–) (1/4)
elongation factor G family (1/11)
elongation factors bacterial (2/3)
initiation factors bacterial (1/4)
aminoacylation, Ala (1/4)
aminoacylation, Asp and Asn (1/3)
aminoacylation, Glu and Gln (3/8)
aminoacylation, Lys (1/3)
-0.00050
-0.00025
0.00000
0.00025
Slope following perturbation

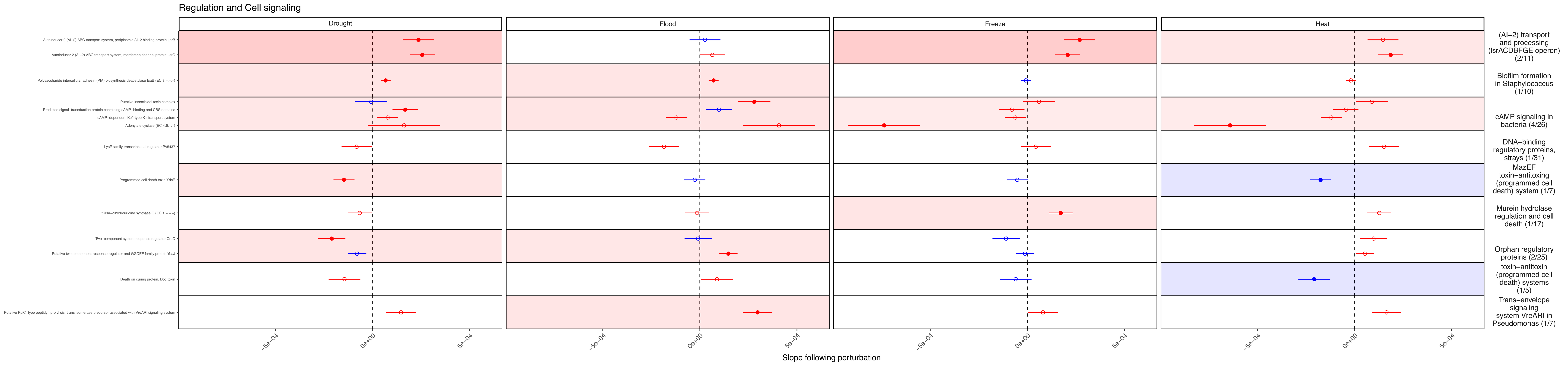
Regulation and Cell signaling
Drought
Flood
Freeze
Heat
Autoinducer 2 (AI–2) ABC transport system, periplasmic AI–2 binding protein LsrB
Autoinducer 2 (AI–2) ABC transport system, membrane channel protein LsrC
Polysaccharide intercellular adhesin (PIA) biosynthesis deacetylase IcaB (EC 3.–.–.–)
Putative insecticidal toxin complex
Predicted signal–transduction protein containing cAMP–binding and CBS domains
cAMP–dependent Kef–type K+ transport system
Adenylate cyclase (EC 4.6.1.1)
LysR family transcriptional regulator PA5437
Programmed cell death toxin YdcE
tRNA–dihydrouridine synthase C (EC 1.–.–.–)
Two–component system response regulator CreC
Putative two–component response regulator and GGDEF family protein YeaJ
Death on curing protein, Doc toxin
Putative PpiC–type peptidyl–prolyl cis–trans isomerase precursor associated with VreARI signaling system
(AI–2) transport and processing (lsrACDBFGE operon) (2/11)
Biofilm formation in Staphylococcus (1/10)
cAMP signaling in bacteria (4/26)
DNA–binding regulatory proteins, strays (1/31)
MazEF toxin–antitoxing (programmed cell death) system (1/7)
Murein hydrolase regulation and cell death (1/17)
Orphan regulatory proteins (2/25)
toxin–antitoxin (programmed cell death) systems (1/5)
Trans–envelope signaling system VreARI in Pseudomonas (1/7)
−5e−04
0e+00
5e−04
Slope following perturbation

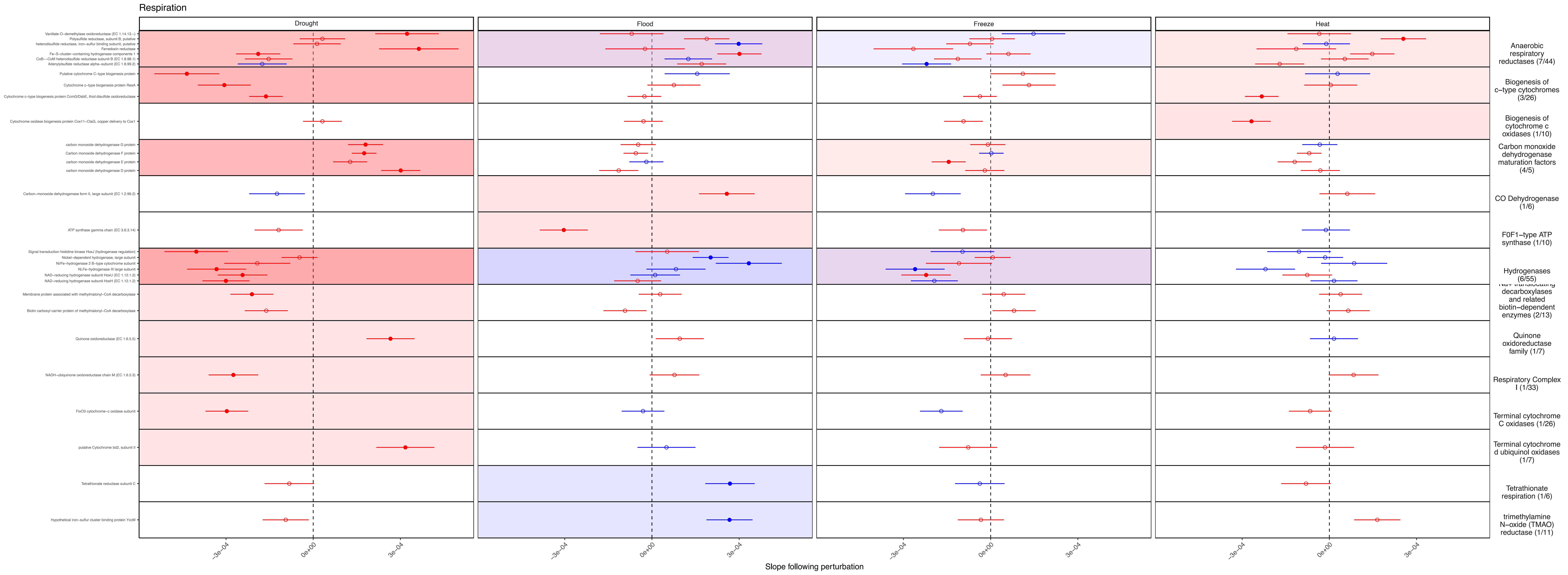

Respiration
Drought
Flood
Freeze
Heat
Vanillate O–demethylase oxidoreductase (EC 1.14.13.–)
Polysulfide reductase, subunit B, putative
heterodisulfide reductase, iron–sulfur binding subunit, putative
Ferredoxin reductase
Fe–S–cluster–containing hydrogenase components 1
CoB––CoM heterodisulfide reductase subunit B (EC 1.8.98.1)
Adenylylsulfate reductase alpha–subunit (EC 1.8.99.2)
Putative cytochrome C–type biogenesis protein
Cytochrome c–type biogenesis protein ResA
Cytochrome c–type biogenesis protein CcmG/DsbE, thiol:disulfide oxidoreductase
Cytochrome oxidase biogenesis protein Cox11–CtaG, copper delivery to Cox1
carbon monoxide dehydrogenase G protein
Carbon monoxide dehydrogenase F protein
carbon monoxide dehydrogenase E protein
carbon monoxide dehydrogenase D protein
Carbon–monoxide dehydrogenase form II, large subunit (EC 1.2.99.2)
ATP synthase gamma chain (EC 3.6.3.14)
Signal transduction histidine kinase HoxJ (hydrogenase regulation)
Nickel–dependent hydrogenase, large subunit
Ni/Fe–hydrogenase 2 B–type cytochrome subunit
Ni,Fe–hydrogenase III large subunit
NAD–reducing hydrogenase subunit HoxU (EC 1.12.1.2)
NAD–reducing hydrogenase subunit HoxH (EC 1.12.1.2)
Membrane protein associated with methylmalonyl–CoA decarboxylase
Biotin carboxyl carrier protein of methylmalonyl–CoA decarboxylase
Quinone oxidoreductase (EC 1.6.5.5)
NADH–ubiquinone oxidoreductase chain M (EC 1.6.5.3)
FixO3 cytochrome–c oxidase subunit
putative Cytochrome bd2, subunit II
Tetrathionate reductase subunit C
Hypothetical iron–sulfur cluster binding protein YccM
Anaerobic respiratory reductases (7/44)
Biogenesis of c–type cytochromes (3/26)
Biogenesis of cytochrome c oxidases (1/10)
Carbon monoxide dehydrogenase maturation factors (4/5)
CO Dehydrogenase (1/6)
F0F1–type ATP synthase (1/10)
Hydrogenases (6/55)
Na+ translocating decarboxylases and related biotin–dependent enzymes (2/13)
Quinone oxidoreductase family (1/7)
Respiratory Complex I (1/33)
Terminal cytochrome C oxidases (1/26)
Terminal cytochrome d ubiquinol oxidases (1/7)
Tetrathionate respiration (1/6)
trimethylamine N–oxide (TMAO) reductase (1/11)
−3e−04
0e+00
3e−04
Slope following perturbation

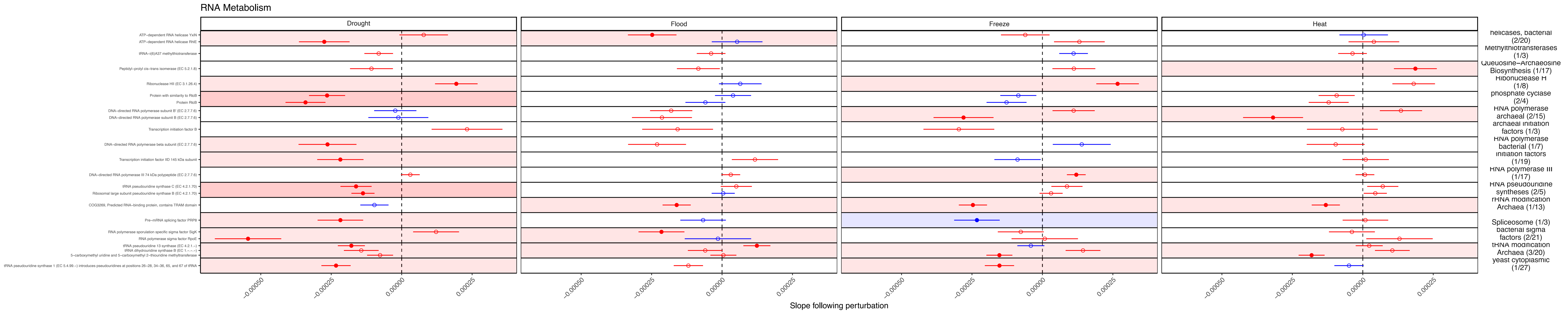

RNA Metabolism
Drought
Flood
Freeze
Heat
ATP–dependent RNA helicase YxiN
ATP–dependent RNA helicase RhlE
tRNA–i(6)A37 methylthiotransferase
Peptidyl–prolyl cis–trans isomerase (EC 5.2.1.8)
Ribonuclease HII (EC 3.1.26.4)
Protein with similarity to RtcB
Protein RtcB
DNA–directed RNA polymerase subunit B' (EC 2.7.7.6)
DNA–directed RNA polymerase subunit B (EC 2.7.7.6)
Transcription initiation factor B
DNA–directed RNA polymerase beta subunit (EC 2.7.7.6)
Transcription initiation factor IID 145 kDa subunit
DNA–directed RNA polymerase III 74 kDa polypeptide (EC 2.7.7.6)
tRNA pseudouridine synthase C (EC 4.2.1.70)
Ribosomal large subunit pseudouridine synthase B (EC 4.2.1.70)
COG3269, Predicted RNA–binding protein, contains TRAM domain
Pre–mRNA splicing factor PRP8
RNA polymerase sporulation specific sigma factor SigK
RNA polymerase sigma factor RpoE
tRNA pseudouridine 13 synthase (EC 4.2.1.–)
tRNA dihydrouridine synthase B (EC 1.–.–.–)
5–carboxymethyl uridine and 5–carboxymethyl 2–thiouridine methyltransferase
tRNA pseudouridine synthase 1 (EC 5.4.99.–) introduces pseudouridines at positions 26–28, 34–36, 65, and 67 of tRNA
helicases, bacterial (2/20)
Methylthiotransferases (1/3)
Queuosine–Archaeosine Biosynthesis (1/17)
Ribonuclease H (1/8)
phosphate cyclase (2/4)
RNA polymerase archaeal (2/15)
archaeal initiation factors (1/3)
RNA polymerase bacterial (1/7)
initiation factors (1/19)
RNA polymerase III (1/17)
RNA pseudouridine syntheses (2/5)
rRNA modification Archaea (1/13)
Spliceosome (1/3)
bacterial sigma factors (2/21)
tRNA modification Archaea (3/20)
yeast cytoplasmic (1/27)
−0.00050
−0.00025
0.00000
0.00025
Slope following perturbation

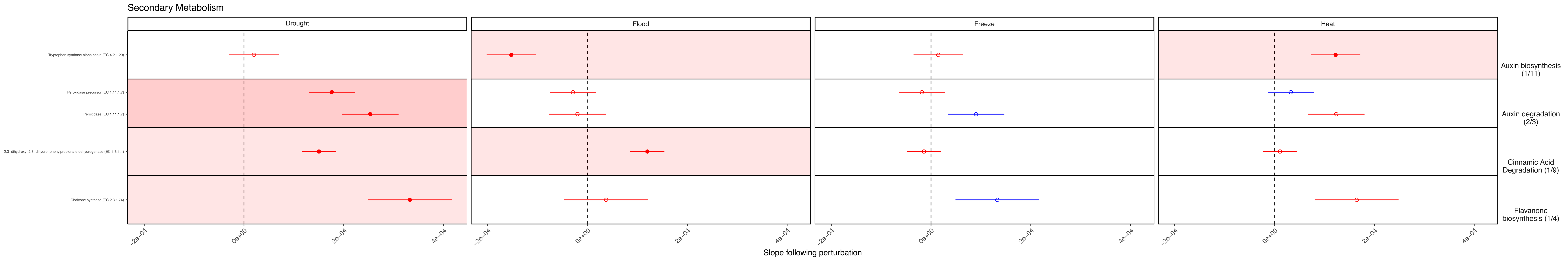
Secondary Metabolism
Drought
Flood
Freeze
Heat
Tryptophan synthase alpha chain (EC 4.2.1.20)
Peroxidase precursor (EC 1.11.1.7)
Peroxidase (EC 1.11.1.7)
2,3−dihydroxy−2,3−dihydro−phenylpropionate dehydrogenase (EC 1.3.1.−)
Chalcone synthase (EC 2.3.1.74)
Auxin biosynthesis (1/11)
Auxin degradation (2/3)
Cinnamic Acid Degradation (1/9)
Flavanone biosynthesis (1/4)
−2e−04
0e+00
2e−04
4e−04
Slope following perturbation

Stress Response

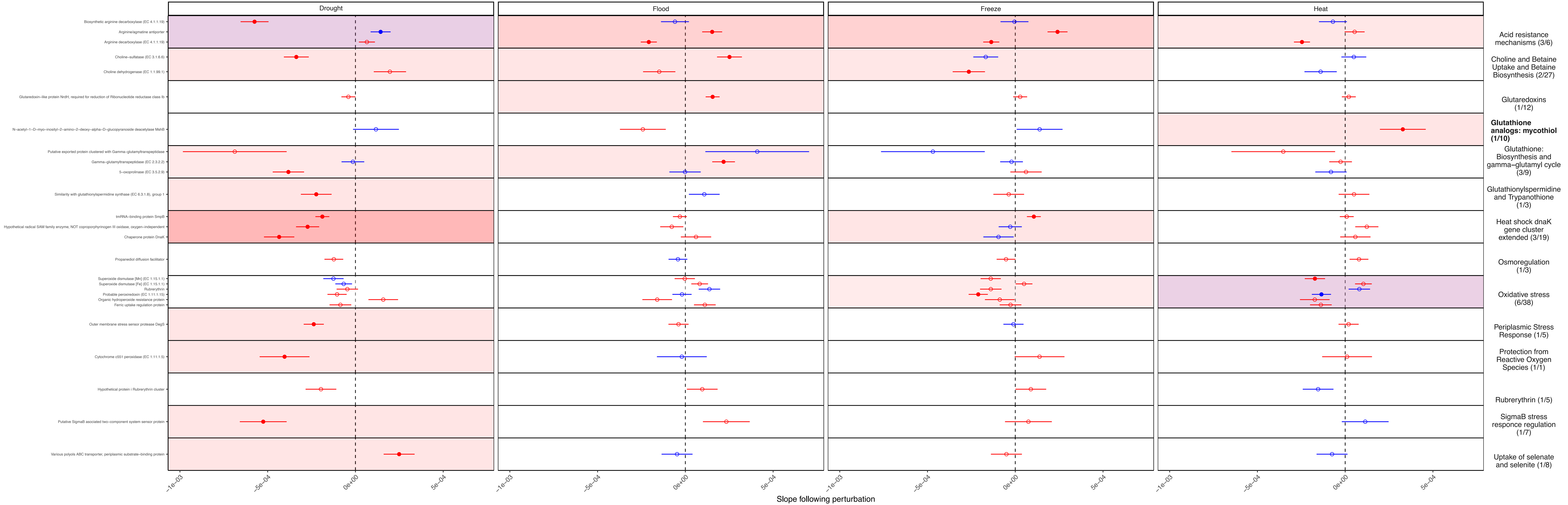

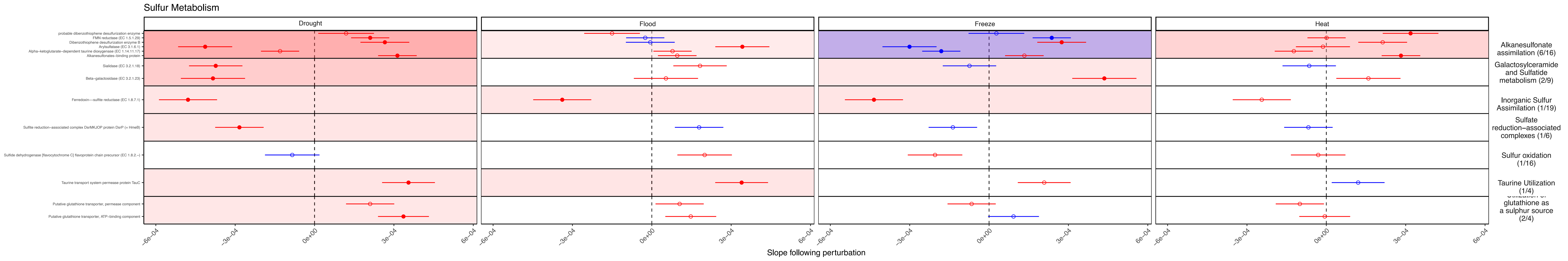

Sulfur Metabolism
Drought
Flood
Freeze
Heat
probable dibenzothiophene desulfurization enzyme
FMN reductase (EC 1.5.1.29)
Dibenzothiophene desulfurization enzyme B
Arylsulfatase (EC 3.1.6.1)
Alpha–ketoglutarate–dependent taurine dioxygenase (EC 1.14.11.17)
Alkanesulfonates–binding protein
Sialidase (EC 3.2.1.18)
Beta–galactosidase (EC 3.2.1.23)
Ferredoxin––sulfite reductase (EC 1.8.7.1)
Sulfite reduction–associated complex DsrMKJOP protein DsrP (= HmeB)
Sulfide dehydrogenase [flavocytochrome C] flavoprotein chain precursor (EC 1.8.2.–)
Taurine transport system permease protein TauC
Putative glutathione transporter, permease component
Putative glutathione transporter, ATP–binding component
Alkanesulfonate assimilation (6/16)
Galactosylceramide and Sulfatide metabolism (2/9)
Inorganic Sulfur Assimilation (1/19)
Sulfate reduction–associated complexes (1/6)
Sulfur oxidation (1/16)
Taurine Utilization (1/4)
Utilization of glutathione as a sulphur source (2/4)
−6e−04
−3e−04
0e+00
3e−04
6e−04
Slope following perturbation

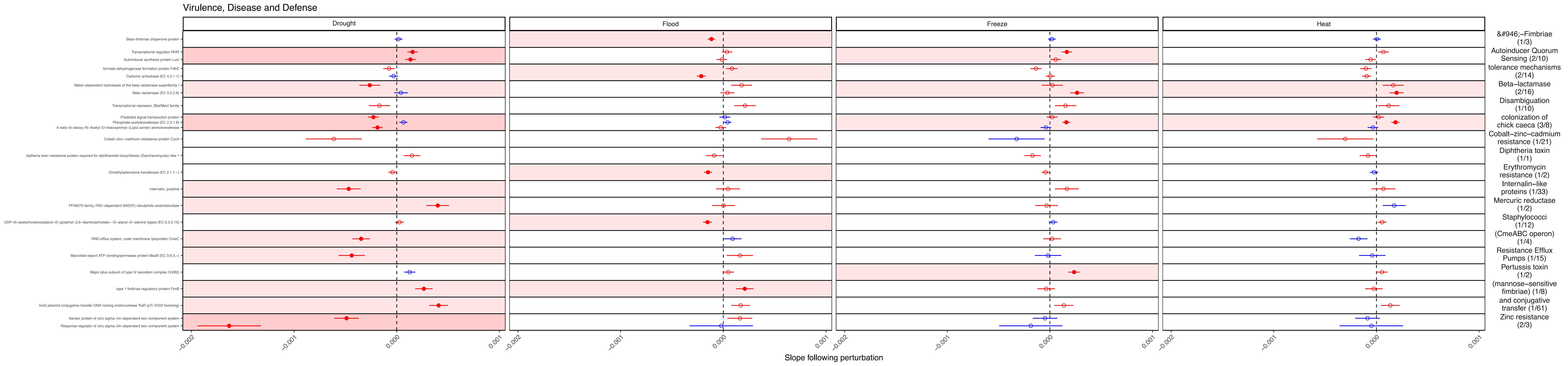

Virulence, Disease and Defense
Drought
Flood
Freeze
Heat
Beta–fimbriae chaperone protein
Transcriptional regulator RhlR
Autoinducer synthesis protein LuxI
formate dehydrogenase formation protein FdhE
Carbonic anhydrase (EC 4.2.1.1)
Metal–dependent hydrolases of the beta–lactamase superfamily I
Beta–lactamase (EC 3.5.2.6)
Transcriptional repressor, BlaI/MecI family
Predicted signal transduction protein
Phosphate acetyltransferase (EC 2.3.1.8)
4–keto–6–deoxy–N–Acetyl–D–hexosaminyl–(Lipid carrier) aminotransferase
Cobalt–zinc–cadmium resistance protein CzcA
diptheria toxin resistance protein required for diphthamide biosynthesis (Saccharomyces)–like 1
Dimethyladenosine transferase (EC 2.1.1.–)
internalin, putative
PF00070 family, FAD–dependent NAD(P)–disulphide oxidoreductase
UDP–N–acetylmuramoylalanyl–D–glutamyl–2,6–diaminopimelate––D–alanyl–D–alanine ligase (EC 6.3.2.10)
RND efflux system, outer membrane lipoprotein CmeC
Macrolide export ATP–binding/permease protein MacB (EC 3.6.3.–)
Major pilus subunit of type IV secretion complex (VirB2)
type 1 fimbriae regulatory protein FimB
IncQ plasmid conjugative transfer DNA nicking endonuclease TraR (pTi VirD2 homolog)
Sensor protein of zinc sigma–54–dependent two–component system
Response regulator of zinc sigma–54–dependent two–component system
β–Fimbriae (1/3)
Autoinducer Quorum Sensing (2/10)
tolerance mechanisms (2/14)
Beta–lactamase (2/16)
Disambiguation (1/10)
colonization of chick caeca (3/8)
Cobalt–zinc–cadmium resistance (1/21)
Diphtheria toxin (1/1)
Erythromycin resistance (1/2)
Internalin–like proteins (1/33)
Mercuric reductase (1/2)
Staphylococci (1/12)
(CmeABC operon) (1/4)
Resistance Efflux Pumps (1/15)
Pertussis toxin (1/2)
(mannose–sensitive fimbriae) (1/8)
and conjugative transfer (1/61)
Zinc resistance (2/3)
–0.002
–0.001
0.000
0.001
Slope following perturbation
